# Comparative Study of PPAR*γ* Targets in Human Extravillous and Villous Cytotrophoblasts

**DOI:** 10.1155/2020/9210748

**Published:** 2020-04-01

**Authors:** Fulin Liu, Christine Rouault, Mickael Guesnon, Wencan Zhu, Karine Clément, Séverine A. Degrelle, Thierry Fournier

**Affiliations:** ^1^Université de Paris, INSERM, UMR-S1139 “Pathophysiology & Pharmacotoxicology of the Human Placenta, Pre & Postnatal Microbiota” (3PHM), Paris F-75006, France; ^2^Fondation PremUp, Paris F-75006, France; ^3^Sorbonne Université, INSERM, “Nutrition et Obesités: Approches Systémiques Research Unit”, Paris F-75013, France; ^4^UMR MIA-PARIS, AgroParisTech-Université Paris-Saclay, Paris F-75005, France; ^5^Assistance Publique-Hôpitaux de Paris, Nutrition Department, Pitié-Salpêtrière Hospital, Paris F-75013, France; ^6^Inovarion, Paris F-75005, France

## Abstract

Trophoblasts, as the cells that make up the main part of the placenta, undergo cell differentiation processes such as invasion, migration, and fusion. Abnormalities in these processes can lead to a series of gestational diseases whose underlying mechanisms are still unclear. One protein that has proven to be essential in placentation is the peroxisome proliferator-activated receptor *γ* (PPAR*γ*), which is expressed in the nuclei of extravillous cytotrophoblasts (EVCTs) in the first trimester and villous cytotrophoblasts (VCTs) throughout pregnancy. Here, we aimed to explore the genome-wide effects of PPAR*γ* on EVCTs and VCTs via treatment with the PPAR*γ*-agonist rosiglitazone. EVCTs and VCTs were purified from human chorionic villi, cultured *in vitro*, and treated with rosiglitazone. The transcriptomes of both types of cells were then quantified using microarray profiling. Differentially expressed genes (DEGs) were filtered and submitted for gene ontology (GO) annotation and pathway analysis with ClueGO. The online tool STRING was used to predict PPAR*γ* and DEG protein interactions, while iRegulon was used to predict the binding sites for PPAR*γ* and DEG promoters. GO and pathway terms were compared between EVCTs and VCTs with clusterProfiler. Visualizations were prepared in Cytoscape. From our microarray data, 139 DEGs were detected in rosiglitazone-treated EVCTs (RT-EVCTs) and 197 DEGs in rosiglitazone-treated VCTs (RT-VCTs). Downstream annotation analysis revealed the similarities and differences between RT-EVCTs and RT-VCTs with respect to the biological processes, molecular functions, cellular components, and KEGG pathways affected by the treatment, as well as predicted binding sites for both protein-protein interactions and transcription factor-target gene interactions. These results provide a broad perspective of PPAR*γ*-activated processes in trophoblasts; further analysis of the transcriptomic signatures of RT-EVCTs and RT-VCTs should open new avenues for future research and contribute to the discovery of possible drug-targeted genes or pathways in the human placenta.

## 1. Introduction

The human placenta serves as a critical bridge between mother and fetus and thus plays a crucial role in maternal and fetal physiology. The placenta is composed mainly of trophoblast cells, which derive from the outer layer of the blastocyst. Certain trophoblasts can be further distinguished as villous cytotrophoblasts (VCTs), whose development progresses along with that of the placenta. In the process of embryo implantation and placenta formation, VCTs that invade the maternal uterus are known as extravillous cytotrophoblasts (EVCTs); these anchor the chorionic villi. Other VCTs differentiate and fuse to form the syncytiotrophoblast layer, which has critical functions in gas and nutrient exchange between the fetus and the mother. Defects in EVCT invasion and VCT differentiation and fusion contribute to a series of gestational diseases, such as fetus-related miscarriage [1], preterm birth [2], and preeclampsia [3]. The causes of and mechanisms behind these diseases have been the focus of much research, but as yet remain unclear.

As a member of the ligand-dependent nuclear receptor superfamily, PPAR*γ* regulates many downstream target genes involved in lipid metabolism, cell differentiation, and tumorigenesis. PPAR*γ* functions by forming a heterodimer with the nuclear receptor retinoid X receptor *α* (RXR*α*) and then binding to the PPAR response element (PPRE) of target genes [4]. It has been reported that a lack of PPAR*γ* leads to defects in trophoblast differentiation and abnormal vasculogenesis in mice [5, 6], and PPAR*γ*^−/−^ embryonic lethality can be rescued via PPAR*γ* transfection in the trophoblast [7]. To be more specific, previous research showed that the activation of PPAR*γ* inhibited the invasion of first-trimester EVCTs, which implicates PPAR*γ* in the regulation of invasion of the decidua [8, 9]. Furthermore, the activation of PPAR*γ* can also induce the differentiation of VCTs isolated from term placenta [10]. Taken together, the current literature on the effects of PPAR*γ* on the regulation of EVCTs and VCTs suggests that it plays a critical role in trophoblast invasion and differentiation but also that these effects differ dramatically among different subtypes of trophoblast. PPAR*γ* thus appears to play a crucial but poorly understood role in placental development.

To explore the role of PPAR*γ* in biological processes, the PPAR*γ*-agonist rosiglitazone has been widely applied to various tissues. In human placenta, rosiglitazone has been used for the study of placental metabolism [11, 12], inflammation [13, 14], antioxidant response [15, 16], and preeclampsia [17]. *In vitro* treatment with rosiglitazone has been shown to reverse inflammation of the placenta that is mediated by the PPAR*γ*-NF-*κ*B pathway [13]. Similarly, rosiglitazone can improve the survival rate of trophoblasts under oxidative stress via its effects on the PPAR*γ* pathway [15]. Other investigations into the activity of this drug have identified new potential target genes of PPAR*γ* [17, 18]. Furthermore, the activation of PPAR*γ* in both first-trimester EVCTs and term VCTs, as described in the research cited above on the influence of PPAR*γ* in trophoblast invasion and differentiation, was also accomplished by this PPAR*γ*-agonist. Taken together, these studies show the enormous potential and benefit of rosiglitazone use in studies of the placenta.

**Figure 1:**
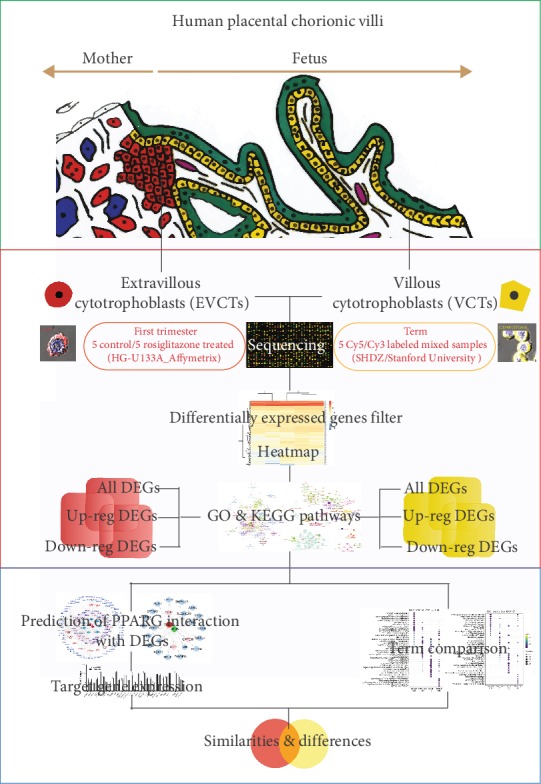
Summary of procedures. Extravillous cytotrophoblasts (EVCTs, HLAG+ cells) ^[^22^]^ and villous cytotrophoblasts (VCTs, CD49f+ cells) were isolated from human first trimester and term placental chorionic villi, respectively, treated with rosiglitazone, and analyzed using microarrays. Differentially expressed genes (DEGs) were filtered for quality control and submitted for annotation. Terms associated with DEGs and predictions of PPAR*γ*-target genes were compared between the rosiglitazone-treated EVCTs and VCTs. PPAR*γ*: peroxisome proliferator-activated receptor *γ*. The top graphic was modified from Handschuh et al. (2007).

In the human placenta, PPAR*γ* is exclusively located in the nuclei of EVCTs during the first trimester and of VCTs throughout pregnancy [19–21]. To date, there is a lack of systematic research on the effects of PPAR*γ* in these tissues and during these developmental periods. Therefore, our purpose here was to investigate the performance of PPAR*γ*-activated trophoblasts by analyzing the transcriptomic signatures of rosiglitazone-treated EVCTs (RT-EVCTs) and VCTs (RT-VCTs). In this study, we isolated EVCTs and VCTs from first trimester and term human chorionic villi, respectively; cultured these cells with rosiglitazone; and quantified the transcriptome of each type of cell using microarray analysis, as shown in Figure 1. Our results provide abundant information on the biological processes and pathways affected by PPAR*γ*, as well as on the specific genes and pathways targeted, and constitute an invaluable knowledge base for future research.

## 2. Materials and Methods

### 2.1. Ethics Statement

Placenta samples in this study were collected with patients' written informed consent, in compliance with the Declaration of Helsinki. Placenta tissues were collected from women with normal pregnancies during the 8-9^th^ gestational weeks and at term (39 gestational weeks). Our ethics committee (CCPRB Paris Cochin no. 18-05) approved the collection of placentas from legal and voluntary terminations of pregnancy in the first trimester as well as of the normal term placentas.

### 2.2. Cell Isolation and Culture

As previously described [22], five effective first-trimester placentas were obtained for EVCT isolation. Villous tissues were rinsed and minced in Ca^2+^-, Mg^2+^-free Hanks' balanced salt solution for membrane removal. Mononucleated VCTs were isolated using digestion with trypsin-DNase and fractionation on a discontinuous Percoll gradient according to the protocol of Kliman et al. [23] and Alsat et al. [24]. In brief, villous tissues were digested in Hanks' balanced salt solution, containing 5 IU/mL of DNase I, 4.2 mM MgSO_4_, 0.25% (wt/vol) trypsin powder (Difco), 100 IU/mL penicillin, 25 mM HEPES, and 100 *μ*g/mL streptomycin (Biochemical Industry), and monitored under invert microscopy. The initial digested solution (consisting mostly of red blood cells) was discarded while the subsequent digested solution (clearly consisting of EVCTs) was retained. A discontinuous Percoll gradient (5–70% in 5% steps) was used to stratify the digested solutions; the middle layer (which included EVCTs) was retained for further analysis. The purified EVCTs were diluted with Dulbecco's modified Eagle's medium (DMEM), with 2 mM glutamine, 100 IU/mL penicillin, 100 mg/mL streptomycin, and 10% decomplemented fetal calf serum (FCS), to a final density of 0.9 × 10^6^ cells/mL in 60 mm diameter plastic tissue culture dishes (Techno Plastic Products, Switzerland). In preparation for culturing, culture plates (Techno Plastic Products, Switzerland) were coated with Matrigel™ (7 *μ*g/cm^2^; Collaborative Biomedical Products, Le Pont de Claix, France), then seeded with EVCTs at a density of 5 × 10^4^ cells/cm^2^. To maintain continuous culture conditions, DMEM-F12 medium was used that contained 10% heat-inactivated fetal calf serum (FCS), Glutamax, 100 *μ*g/mL streptomycin, and 100 IU/mL penicillin (Invitrogen). Plates were incubated for 2 h at 37°C and 5% CO_2_; then, nonadherent EVCTs were rinsed off. At this point, fresh medium with or without 1 *μ*M rosiglitazone (Cayman) dissolved at 1 mM in ethanol (treatment) or 0.1% ethanol (vehicle) was added for another 24 h of incubation.

VCTs were isolated from five term placentas using the following procedures. Placentas were oriented with the maternal side facing upwards, and tissues were sampled at a depth of 1.5 cm, half the distance from the edge to the centre. Villous tissues were rinsed, minced, digested, and purified using the steps described above. Culture dishes containing 0.9 × 10^6^ cells/mL were placed in a humidified incubator at 37°C under 5% CO_2_ for 3 h. Nonadherent VCTs were rinsed off, fresh medium with or without 1 *μ*M rosiglitazone (Cayman) dissolved at 1 mM in ethanol (treatment) or 0.1% ethanol (vehicle) was added, and dishes were incubated for another 24 h.

### 2.3. Microarray Experiments

After 24 h of incubation, RT-EVCTs and control EVCTs were harvested for microarray experiments. Cell RNA was extracted using TRIzol® reagents (Invitrogen) and purified using RNeasy® Mini Kits (Qiagen). RNA integrity and purity were examined with a 2100 Bioanalyzer with the RNA 6000 LabChip kit (Agilent Technologies). The U133A 2.0 GeneChip (Affymetrix, Inc.) was used for gene expression detection according to the manufacturer's manual. From the 22,000 probe sets on the gene chip, 14,500 genes were detected.

RT-VCTs and control VCTs were likewise harvested after 24 h of incubation for microarray experiments; RNA extraction, purification, and quality control were performed as described. The SHDZ gene chip (Stanford University) was used for gene detection as described in [25]: for each sample, the MessageAmp RNA kit (Ambion) was used, with 1 *μ*g total RNA, for RNA amplification, and 3 *μ*g amplified RNA were then labeled with Cy-dye using the 26 CyScribe first-strand cDNA labeling kit (Amersham Biosciences). Amplified RNA from rosiglitazone-treated VCTs was labeled with Cy5, and amplified RNA from control VCTs was labeled with Cy3. A Microcon YM 30 column (Millipore) was used to purify and concentrate the labeled mixture (Cy5 and Cy3) after additional modifications with human cot-1, yeast tRNA, and poly A. The probes were denatured and the mixture was hybridized at 65°C overnight in a sealed humidified hybridization chamber, then rinsed with 1XSSC, 2XSSC, 0.03% SDS, and 0.2% SDS solutions for 2 min each. Arrays were scanned with a GenePrix 4000A microarray scanner (Axon Instruments).

### 2.4. Data Processing

Since gene expression in EVCTs and VCTs was detected using different microarray platforms, different procedures were followed for data processing. For EVCT gene expression, which was quantified using the GeneChip (U133A 2.0, Affymetrix) application, data processing used the following filter thresholds: (i) percentage of missing data was no more than 50%, (ii) threshold to identify up- and downregulated genes for statistical comparison was set to a fold change of 1.5, (iii) maximum false discovery rate (FDR) was set to 5%, and (iv) fold change of one gene was equal to mean of treated groups minus mean of control groups and then divided by the minimum value from all (fold change = (mean (treated)–mean (control))/minimum (treated, control)) [22]. For VCT gene expression, which was measured using the SHDZ GeneChip/Stanford University (GPL21609) application, data processing used the following filter thresholds: (i) background-corrected data were log2-transformed and subjected to the Loess normalization method [11], (ii) differentially expressed genes (DEGs) were determined via the significance analysis of microarrays (SAM) method [26], and (iii) the maximum false discovery rate (FDR) was set to 1%, without a fold change threshold imposed [27].

### 2.5. GO and Pathway Enrichment Analyses

ClueGO is a Cytoscape plug-in application for the functional classification of genes [28]. Our analysis used Cytoscape version 3.7.1 (The Cytoscape Consortium, New York, NY) and ClueGO version 2.5.4 (released 28 Feb 2019), with the simultaneous update of gene ontology (GO) terms. Using ClueGO, we recovered the GO terms associated with the dataset of all DEGs as well as of up- or downregulated DEGs only; this same application was also used for KEGG and Reactome pathway analysis. GO terms were compared between EVCTs and VCTs using the R package clusterProfiler (version 3.9, synced to latest GO terms and pathways) [29]. For term comparison in clusterProfiler, 10 category terms for each group were selected for inclusion in charts. Instead, ClueGO analyses were based on approximately 30 terms per group in order to generate more detailed visualizations. *P* values lower than 0.05 identified significant enrichment.

### 2.6. Protein-Protein Interaction (PPI) Network

The STRING database (https://string-db.org) was used to analyze the interactions of DEG-encoded proteins and construct a PPI network. For this, the significant confidence score was set to greater than 0.4. Cytoscape was used to visualize and organize the PPI network. Proteins interacting with PPAR*γ* or RXR*α* were indicated by different colors, and shapes were used to represent different groups. Binding site interactions between transcription factors and target genes were predicted by the Cytoscape plug-in iRegulon (based on the TRANSFAC database; version 1.3). Putative regulatory regions were defined as 10 kb around transcription starting sites. The FDR was set to 0.1% to verify the interaction. The resulting chart was modified in Cytoscape using red to indicate upregulated genes and blue to indicate downregulated genes.

## 3. Results

### 3.1. Gene Expression Profiling of RT-EVCTs and RT-VCTs

**Figure 2:**
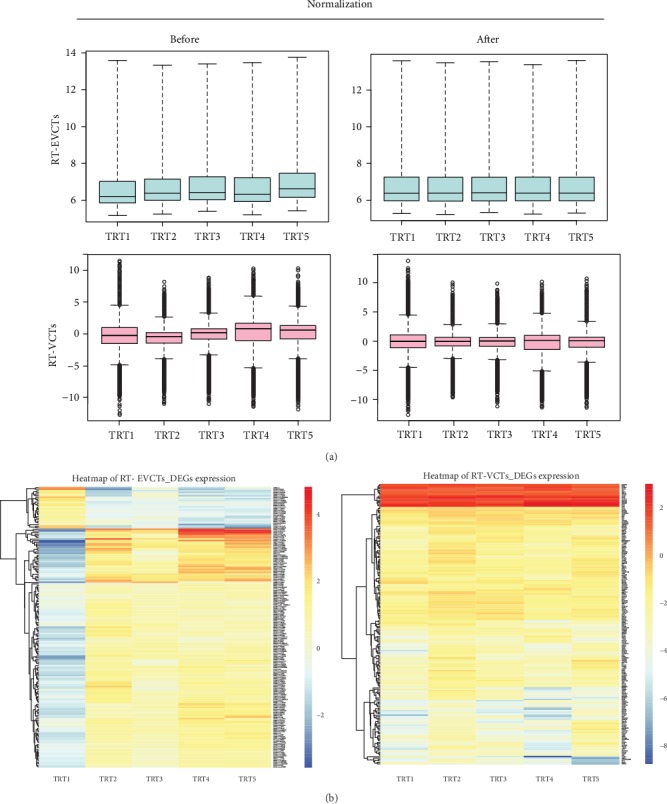
Microarray data normalization and DEG heatmap of RT-EVCTs and RT-VCTs. (a) RT-EVCT gene expression microarray was performed with the Affymetrix GeneChip while the RT-VCT microarray used the SHDZ/Stanford University chip. DEGs were detected based on the thresholds of 1.5-fold change and 5% FDR for the RT-EVCT microarray matrix; a threshold of 1% FDR was applied for the RT-VCT microarray matrix. The Loess normalization method was used to normalize both datasets. Box plots represent microarray data before and after normalization, with blue indicating data from RT-EVCTs and pink data from RT-VCTs. (b) Heatmaps of five independent samples of RT-EVCTs and RT-VCTs. Upregulated DEGs are represented in red and downregulated DEGs in blue. DEGs: differentially expressed genes; RT-EVCTs: rosiglitazone-treated extravillous cytotrophoblasts; RT-VCTs: rosiglitazone-treated villous cytotrophoblasts; FDR: false discovery rate; TRT: treated.

Microarrays were used to characterize gene expression in EVCTs and VCTs with or without rosiglitazone treatment. Our microarray data have been deposited in the Gene Expression Omnibus public repository (https://www.ncbi.nlm.nih.gov/geo/; EVCT microarray data under accession number GSE28426, VCT microarray data under accession number GSE137434). Gene expression profiles of the rosiglitazone-treated (TRT) samples of EVCTs and VCTs were normalized (Figure 2(a)). Four of the five independent RT-EVCT samples yielded consistent results, with one sample appearing slightly different; instead, all five independent RT-VCT samples yielded similar results. Next, DEGs were detected based on thresholds for both fold change in expression levels and FDR. In RT-EVCTs, a total of 139 genes were identified as DEGs (*P* < 0.05), of which 114 genes were upregulated (red) and 25 genes were downregulated (blue). In RT-VCTs, a total of 197 genes were identified as DEGs (*P* < 0.05), of which 181 genes were upregulated (red) and 16 genes were downregulated (blue) (Figure 2(b)).

### 3.2. Gene Ontology and Pathway Terms of all DEGs from RT-EVCTs and RT-VCTs

**Figure 3:**
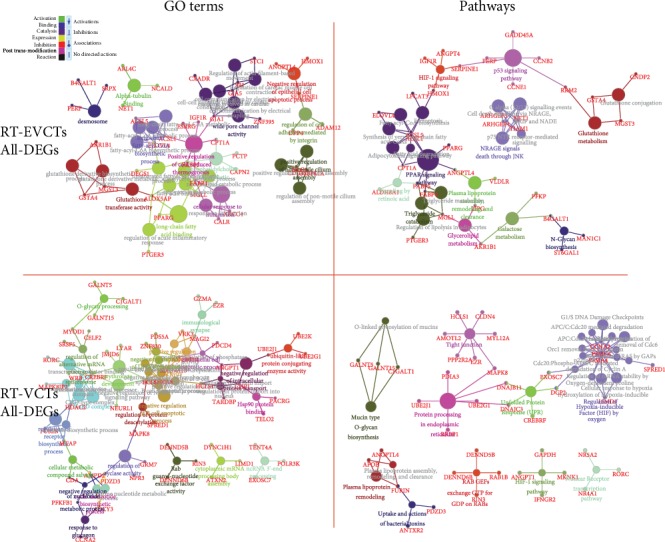
GO and pathway terms associated with all DEGs in RT-EVCTs and RT-VCTs. All DEGs were submitted separately according to their cell type of origin to ClueGO with the default parameters. GO and pathway enrichment were set up for analysis. DEGs were classified by three ways: by GO biological process, GO molecular function, and GO cellular component. The KEGG and Reactome database was consulted to determine pathway enrichment. An exhaustive list of all terms (including those not shown above) can be found in supplementary materials (Tables [Supplementary-material supplementary-material-1]). DEGs: differentially expressed genes; RT-EVCTs: rosiglitazone-treated extravillous cytotrophoblasts; RT-VCTs: rosiglitazone-treated villous cytotrophoblasts; GO: gene ontology; KEGG: Kyoto encyclopedia of genes and genomes.

The entire set of DEGs from RT-EVCTs and RT-VCTs was separated by cell type of origin and submitted independently to ClueGO with the default parameters. GO and pathway enrichment were set up for analysis. DEGs were classified by three ways: by GO biological process, GO molecular function, and GO cellular component. Enriched pathways were identified through a search of the KEGG and Reactome databases. The results are visualized in Figure 3.

Among the DEGs identified in RT-EVCTs, the main GO biological processes represented were “negative regulation of epithelial cell apoptotic process,” “long-chain fatty-acyl-CoA biosynthetic process,” and “phosphatidylcholine biosynthetic process.” For the same group of DEGs, the GO molecular functions were mainly classified as “alpha-tubulin binding,” “wide pore channel activity,” “positive regulation of cold-induced thermogenesis,” “glutathione transferase activity,” “long-chain fatty acid binding,” “regulation of cell adhesion mediated by integrin,” and “positive regulation of non-motile cilium assembly.” Finally, the GO cellular component that was most associated with these DEGs was “desmosome.” In the pathway enrichment analysis of RT-EVCTs, DEGs were mainly associated with the terms “HIF-1 signaling pathway,” “p53 signaling pathway,” “glutathione metabolism,” “NRAGE signals death through JNK,” “PPAR signaling pathway,” “plasma lipoprotein assembly,” and “remodeling and clearance”.

In the analysis of GO terms associated with the RT-VCT dataset, DEGs were mainly involved in the following biological processes: “regulation of receptor biosynthetic process,” “negative regulation of nucleotide metabolic process,” “cyclic nucleotide biosynthetic process,” and “negative regulation of B cell apoptotic process.” The molecular functions of this same group of DEGs were mainly linked to “negative regulation of DNA replication,” “regulation of protein deacetylation,” “ubiquitin-like protein conjugating enzyme activity,” “Hsp90 protein binding,” “negative regulation of intracellular protein transport,” and “positive regulation of phosphoprotein.” With respect to GO cellular components, DEGs were mainly associated with the terms “NuRD complex,” “cellular metabolic compound salvage,” and “immunological synapse.” Finally, the pathway enrichment analysis of RT-VCTs revealed that DEGs were mainly involved in “tight junction,” “regulation of HIF by oxygen,” “unfolded protein response,” “HIF-1 signaling pathway,” “nuclear receptor transcription pathway,” and “plasma lipoprotein remodeling”.

### 3.3. GO and Pathway Terms Associated with Upregulated DEGs in RT-EVCTs and RT-VCTs

**Figure 4:**
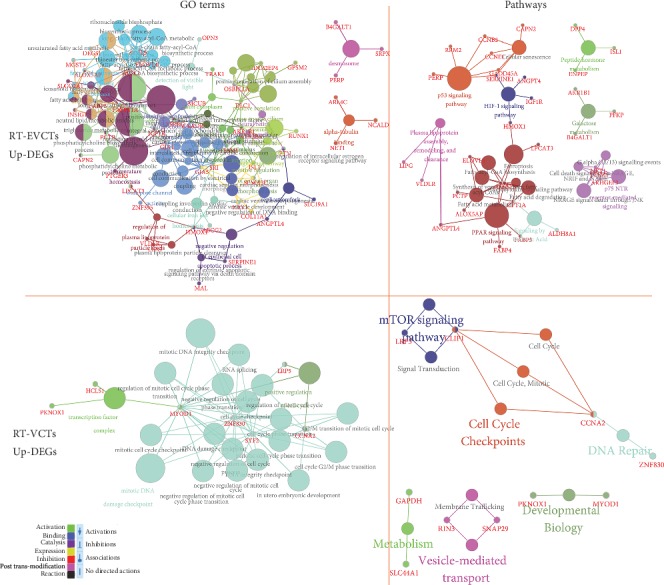
GO and pathway terms associated with DEGs that were upregulated in RT-EVCTs and RT-VCTs. These DEGs were submitted separately to ClueGO by their cell type of origin with the default parameters. GO and pathway enrichment were set up for analysis. Upregulated DEGs were classified by three ways: by their GO biological process, GO molecular function, and GO cellular component. The KEGG and Reactome databases were consulted to determine pathway enrichment. All additional enrichment terms (not shown above) can be found in supplementary materials (Tables [Supplementary-material supplementary-material-1]). DEGs: differentially expressed genes; RT-EVCTs: rosiglitazone-treated extravillous cytotrophoblasts; RT-VCTs: rosiglitazone-treated villous cytotrophoblasts; GO: gene ontology; KEGG: Kyoto encyclopedia of genes and genomes.

DEGs that were upregulated in RT-EVCTs and RT-VCTs were submitted separately to ClueGO following the same procedure as described above. The results are visualized in Figure 4. In RT-EVCTs, upregulated DEGs were mainly associated with the GO biological processes “fatty acid derivative biosynthetic process” and “negative regulation of epithelial cell apoptotic process,” and the GO molecular functions “positive regulation of insulin secretion,” “temperature homeostasis,” “wide pore channel activity,” “nuclear receptor activity,” and “regulation of plasma lipoprotein particles levels.” The main GO cellular components implicated in the activity of these DEGs were “desmosome” and “intrinsic component of mitochondrial membrane.” Finally, the pathway enrichment analysis indicated that upregulated DEGs in RT-EVCTs were mainly involved in the “p53 signaling pathway,” “HIF-1 signaling pathway,” “peptide hormone metabolism,” “PPAR signaling pathway,” “p57 NTR receptor-mediated signaling,” and “signaling by retinoic acid”.

Instead, from the DEGs that were upregulated in RT-VCTs, no significant GO biological process was identified. In the classification of GO molecular functions, these DEGs were mainly linked with “positive regulation of cell cycle” and “mitotic DNA damage checkpoint,” and the most significant GO cellular component was “transcription factor complex.” In the pathway enrichment analysis, upregulated DEGs in RT-VCTs were mainly associated with the terms “mTOR signaling pathway,” “cell cycle checkpoints,” “DNA repair,” “developmental biology,” “metabolism,” and “vesicle-mediated transport”.

### 3.4. GO and Pathway Terms Associated with Downregulated DEGs in RT-EVCTs and RT-VCTs

**Figure 5:**
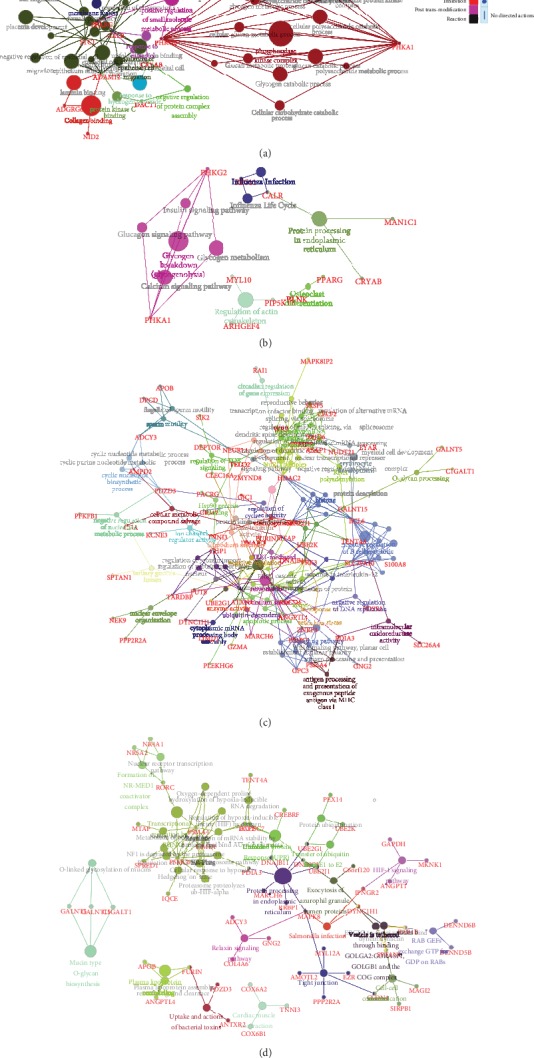
GO and pathway terms associated with DEGs that were downregulated in RT-EVCTs and RT-VCTs, respectively, compared to controls. Downregulated DEGs of RT-EVCTs (a, b) and RT-VCTs (c, d) were submitted to ClueGO separately, with the default parameters. GO and pathway enrichment were set up for analysis. DEGs were classified by three ways: by GO biological process, GO molecular function, and GO cellular component (a, c). Pathway enrichment was determined via comparison with the KEGG database (b, d). (a) The GO terms most associated with DEGs that were downregulated in RT-EVCTs. (b) The pathways that were most enriched among the downregulated DEGs in RT-EVCTs. (c) The GO terms most associated with DEGs that were downregulated in RT-VCTs. (d) The pathways that were most enriched among the downregulated DEGs in RT-VCTs. An exhaustive list of all associated terms (including those not pictured above) can be found in supplementary materials (Tables [Supplementary-material supplementary-material-1]). DEGs: differentially expressed genes; RT-EVCTs: rosiglitazone-treated extravillous cytotrophoblasts; RT-VCTs: rosiglitazone-treated villous cytotrophoblasts; GO: gene ontology; KEGG: Kyoto encyclopedia of genes and genomes.

DEGs that were downregulated in RT-EVCTs and RT-VCTs with respect to controls were submitted to ClueGO using the same procedure as described above. Results are visualized in Figure 5. In RT-EVCTs, downregulated DEGs were mainly associated with the GO biological process “positive regulation of small molecular metabolic process;” the GO molecular functions “membrane fusion,” “regulation of epithelial cell migration,” “response to estriol,” and “protein kinase binding;” and the GO cellular component “phosphorylase kinase complex.” From the analysis of pathway enrichment based on the KEGG and Reactome database, downregulated DEGs in RT-EVCTs appeared to be mainly associated with pathways linked with “glycogen breakdown,” “influenza infection,” “protein processing in endoplasmic reticulum,” and “regulation of actin cytoskeleton”.

Instead, DEGs that were downregulated in RT-VCTs were mainly involved in the GO biological processes “cyclic nucleotide biosynthetic process,” “negative regulation of nucleotide metabolic process,” “ncRNA 3'-end processing,” and “O-glycan processing;” the GO molecular functions “nuclear receptor activity,” “histone deacetylation,” “regulation of TOR signaling,” “Hsp90 protein binding,” “ion channel regulator activity,” “nuclear envelope organization,” and “peptidyl-threonine modification;” and the GO cellular component “organellar ribosome.” In the pathway enrichment analysis of RT-VCTs, downregulated DEGs were mainly associated with the “HIF-1 signaling pathway,” “transfer of ubiquitin from E1 to E3,” “cell-cell communication,” “transcription regulation of RUNX3,” and “formation of NR-MED1 coactivator complex”.

### 3.5. Comparison of GO Terms Associated with Tissue-Specific or Tissue-Generalist DEGs

Next, we wanted to determine the extent to which the cellular processes affected by rosiglitazone treatment were specific to either EVCTs or VCTs, and which instead were present in both tissue types. To do this, we characterized the up- and downregulated DEGs of RT-EVCTs and RT-VCTs separately using clusterProfiler, using information from the GO and KEGG databases, as well as the Disease Ontology (DO) and Disease Gene Network (DisGeNET) databases. Terms appearing in at least three columns were thought important in both, while terms appearing only in the RT-EVCT or RT-VCT dataset were labelled tissue-specific; significance was determined by *P* values less than 0.05.

**Figure 6:**
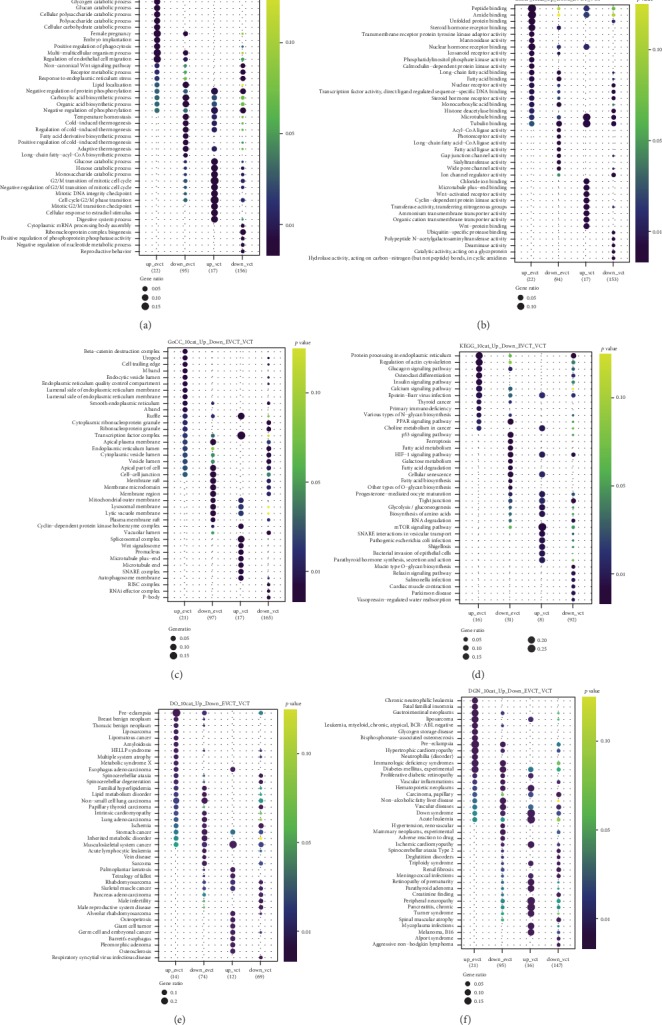
Comparison of enriched GO terms between RT-EVCTs and RT-VCTs. Up and downregulated DEGs of RT-EVCTs and RT-VCTs were submitted separately to analysis in clusterProfiler, for a total of four groups. GO and pathway enrichment were set up for analysis. DEGs were classified by their associated (a) GO biological process, (b) GO molecular function, and (c) GO cellular component. DEGs were further compared with the (d) KEGG database to characterize pathway enrichment, (e) the Disease Ontology (DO) gene set, and (f) the Disease Gene Network (DisGeNET) database. For the purpose of visualization, the top ten categories of enriched terms were included for each gene set. A *P* value less than 0.05 determined significance. DEGs: differentially expressed genes; RT-EVCTs: rosiglitazone-treated extravillous cytotrophoblasts; RT-VCTs: rosiglitazone-treated cytotrophoblasts; GO: gene ontology; KEGG: Kyoto encyclopedia of genes and genomes.

In both RT-EVCTs and RT-VCTs, the GO biological processes “regulation of endothelial cell migration,” “non-canonical Wnt signaling pathway,” “receptor metabolic process,” “negative regulation of protein phosphorylation,” and “metabolism process” appeared to play important roles. Instead, processes specific to RT-EVCTs included “glycogen catabolic process,” “cellular carbohydrate catabolic process,” “embryo implantation,” “fatty acid derivative biosynthetic process,” and “long-chain fatty-acyl-CoA biosynthetic process,” while those specific to RT-VCTs were “cytoplasmic mRNA processing body assembly,” “ribonucleoprotein complex biogenesis,” “positive regulation of phosphoprotein phosphatase activity,” and “negative regulation of nucleotide metabolic process” (Figure 6(a)).

The GO molecular functions “nuclear hormone receptor binding,” “long-chain fatty acid binding,” “fatty acid binding,” “nuclear activity,” and “transcription factor activity” seemed to be important in both RT-EVCTs and RT-VCTs. Functions specific to RT-EVCTs included “steroid hormone receptor binding,” “eicosanoid receptor activity,” “phosphatidylinositol phosphate kinase activity,” and “fatty acid ligase activity,” while those specific to RT-VCTs were “Wnt-activated receptor activity,” “cyclin-dependent protein kinase activity,” “transferase activity,” and “ubiquitin-specific protease activity” (Figure 6(b)).

Both tissue types shared the significant GO cellular components “smooth endoplasmic reticulum,” “ruffle,” “transcription factor complex,” “apical plasma membrane,” “lumen,” and “cell-cell junction.” Instead, the component terms “beta-catenin destruction complex,” “M band,” “integral component of lumenal side of endoplasmic reticulum membrane,” and “A band” were found only in RT-EVCTs, while “spliceosomal complex,” “Wnt signalosome,” “pronucleus,” “microtubule end,” and “autophagosome membrane” appeared to be specific to RT-VCTs (Figure 6(c)).

Through a search of the KEGG database, the following pathways appeared to be important in both tissue types: “protein processing in endoplasmic reticulum,” “glucagon signaling pathway,” “Epstein-Barr virus infection,” “PPAR signaling pathway,” “HIF-1 signaling pathway,” “progesterone-mediated oocyte maturation,” and “mTOR signaling pathway.” Pathway terms specific to RT-EVCTs included “primary immunodeficiency” and “fatty acid metabolism,” while those specific to RT-VCTs were linked with “bacterial invasion of epithelial cells” and “parathyroid hormone synthesis, secretion, and action” (Figure 6(d)).

From the Disease Ontology database, the terms “preeclampsia,” “HELLP syndrome,” “spinocerebellar ataxia,” “familial hyperlipidemia,” “lipid metabolism disorder,” and “musculoskeletal system cancer” were important in both EVCTs and VCTs. Terms specific to RT-EVCTs included “breast benign neoplasm,” “thoracic benign neoplasm,” “lipomatous cancer,” “amyloidosis,” and “vein disease,” while those specific to RT-VCTs were “alveolar rhabdomyosarcoma,” “osteopetrosis,” “giant cell tumor,” and “germ cell and embryonal cancer” (Figure 6(e)).

From a search of the DisGeNET database, the terms “preeclampsia,” “hypertrophic cardiomyopathy,” “immunologic deficiency syndromes,” “diabetes mellitus,” “vascular inflammations,” “hematopoietic neoplasms,” “non-alcoholic fatty liver disease,” “vascular disease,” “ischemic cardiomyopathy,” and “triploidy syndrome” were significant for both tissue types. Instead, “chronic neutrophilic leukemia,” “glycogen storage disease,” and “myeloid, chronic, atypical, and BCR-ABL negative leukemia” were specific to RT-EVCTs, and “alport syndrome” and “aggressive non-Hodgkin lymphoma” were specific to RT-VCTs (Figure 6(f)).

### 3.6. PPAR*γ* Interactions with DEGs of RT-EVCTs and RT-VCTs

**Figure 7:**
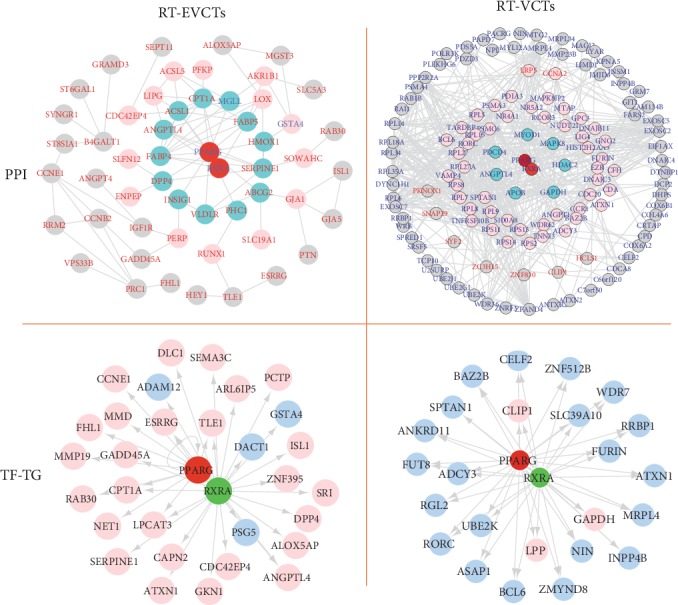
Interactions of the PPAR*γ* and RXR*α* complex with DEGs of RT-EVCTs and RT-VCTs. Predictions were made of protein-protein interactions between PPAR*γ* and DEG-encoded proteins, as well as of the transcription factor-target gene (TF-TG) interactions of PPAR*γ* with DEG promoters. The PPAR*γ* and RXR*α* (heterodimeric nuclear receptor partner of PPAR*γ*) complex, together with the DEGs recovered in this study, were submitted to the STRING online tool. Visualizations were modified in Cytoscope to depict hierarchical interactions and gene expression. For hierarchical protein-protein interactions, red text represents upregulated genes, blue text represents downregulated genes, blue circles represent direct interactions with the PPAR*γ* complex, red circles represent second-level interactions, and grey circles represent plus-level interactions. TF-TG interactions of the PPAR*γ* complex with DEG promoters were predicted by iRegulon based on the TRANSFAC database. Red circles represent upregulated DEGs and blue circles represent downregulated DEGs. PPAR*γ*: peroxisome proliferator-activated receptor-*γ*; RXR*α*: retinoid x receptor-*α*; RT-EVCTs: rosiglitazone-treated extravillous cytotrophoblasts; RT-VCTs: rosiglitazone-treated villous cytotrophoblasts; DEGs: differentially expressed genes; PPI: protein-protein-interaction; TF-TG: transcription factor-target gene.

Since the gene expression changes we observed here were caused by the activation of PPAR*γ* by rosiglitazone, we next attempted to predict (i) the protein-protein interactions (PPI) of PPAR*γ* with DEG-encoded proteins and (ii) the transcription factor-target gene (TF-TG) interactions of PPAR*γ* with DEG promoters. In RT-EVCTs (Figure 7(a)), the following proteins appeared to interact directly with the PPAR*γ* complex: MGLL, FABP5, HMOX1, SERPINE1, ABCG2, PHC1, VLDLR, INSIG1, DPP4, ANGPTL4, FAPB4, ACSL1, and CPT1A. Instead, ACSL5, PFKP, AKR1B1, LOX, GXTA4, SOWAHC, GJA1, SLC19A1, RUNX1, PERP, ENPEP, SLFN12, CDC42EP, and LIPG participated in secondary interactions. In RT-VCTs (Figure 7(b)), the PPAR*γ* complex interacted directly with MYOD1, MAPK8, HDAC2, GAPDH, APOB, ANGPTL4, and PDCD4 and secondarily with PDIA3, MAPK8IP2, NR4A1, GNG2, and CCR1. Our analysis of TF-TG interactions in RT-EVCTs (Figure 7(c)) predicted that the target genes of the PPAR*γ* complex were the upregulated DEGs DLC1, SEMA3C, ARL6IP5, PCTP, ISL1, ZNF395, SR1, DPP4, ALOX5AP, ANGPL4, CDC42EP4, GKN1, ATXN1, CAPN2, LPCAT3, SERPINE1, NET1, LPCAT3, CPT1A, RAB30, GADD45A, MMP19, FHL1, MMD, CCNE1, and ESRRG, as well as the downregulated DEGs ADAM12, GSTA4, PSG5, and DACT1. The same analysis of RT-VCTs (Figure 7(d)) predicted that the target genes of the PPAR*γ* complex were the upregulated DEGs CLIP1, GAPDH, and LPP, as well as the downregulated DEGs CELF2, ZNF512B, SLC39A10, WDR7, FURIN, RRBP1, ATXN1, MRPL4, INNPP4B, ZMYND8, BCL6, ASAP1, UBE2K, RORC, RGL2, ADCY3, FUT8, ANKRD11, SPTAN1, and BAZ2B.

### 3.7. Expression of Genes Targeted by PPAR*γ* in RT-EVCTs and RT-VCTs

**Figure 8:**
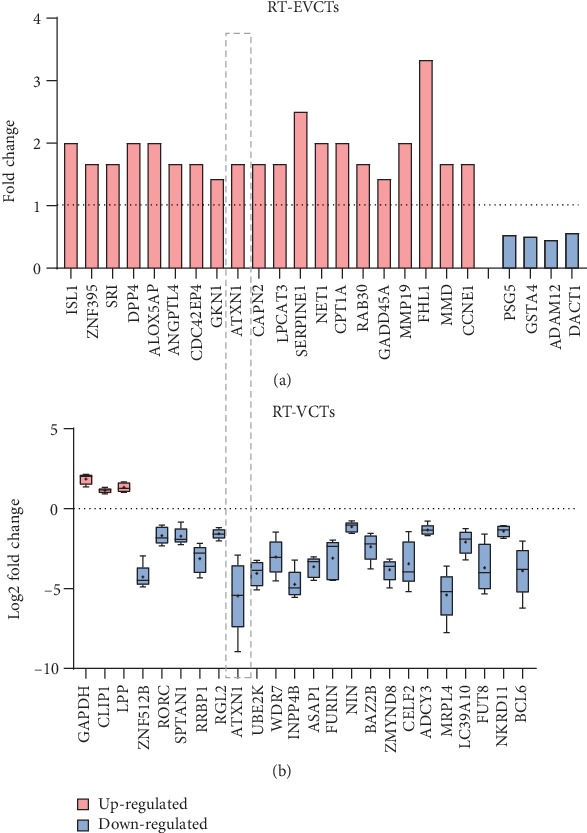
Expression of genes targeted by PPAR*γ* and RXR*α* in RT-EVCTs and RT-VCTs. Gene symbols were retrieved from the normalized gene expression matrix, together with the log2 fold change values in each sample. With these values, boxplots were graphed for (a) RT-EVCTs and (b) RT-VCTs, with upregulation represented in red and downregulation in blue. The grey dashed box indicates the only gene found in both tissue types. PPAR*γ*: peroxisome proliferator-activated receptor-*γ*; RXR*α*: retinoid x receptor-*α*; RT-EVCTs: rosiglitazone-treated extravillous cytotrophoblasts; RT-VCTs: rosiglitazone-treated cytotrophoblasts.

We next filtered our datasets to examine only the DEGs targeted directly by the PPAR*γ* complex, based on the TF-TG predictions described above. The filtered RT-EVCT database contained 26 upregulated and 4 downregulated DEGs (Figure 8(a)), while the filtered RT-VCT database contained 3 upregulated and 21 downregulated DEGs (Figure 8(b)). Only one target gene, ATXN1, was present in both datasets; it was upregulated in RT-EVCTs and downregulated in RT-VCTs (Figure 8).

## 4. Discussion

The human placenta is a critical bridge between mother and fetus, facilitating nutrient exchange and various endocrine and immunological processes. As the cells that form the main part of the placenta, trophoblasts undergo extensive cell differentiation, including invasion, migration, and fusion. Abnormalities in these physiological processes can lead to a series of gestational diseases such as preeclampsia or intrauterine growth restriction. Specifically, both of these disorders appear to be associated with irregularities in the invasion of EVCTs into the maternal uterus, a biological process that is tightly controlled both spatially and temporally [30, 31]. However, the underlying mechanism linking EVCT invasion to gestational dysfunction has yet to be fully investigated. Our team has previously shown the critical influence of activated PPAR*γ* on trophoblasts via treatment of the natural ligands of PPAR*γ* or its specific agonist rosiglitazone [9, 20, 22, 32]. Rosiglitazone is the first synthetic chemical compound to be developed that demonstrates high selectivity for PPAR*γ* (*K*d approximately 40 nM); concentrations of up to 100 *μ*M of this compound have been reported to activate only PPAR*γ* (including the PPAR*α*/*β*/*δ* complex [33]). Moreover, our previous research revealed that a concentration of only 1 *μ*M rosiglitazone led to significant alterations in trophoblast differentiation, with more than 50% inhibition of EVCT invasion [9]. In this study, we treated EVCTs and VCTs with 1 *μ*M rosiglitazone in order to more fully understand the effects of PPAR*γ* on gene expression in these tissues.

Our microarray results for EVCTs were published previously with the aim of identifying significant DEGs for further study [22]. However, this work provided little information about the relative enrichment of pathways and processes among these DEGs and did not include any comparisons with RT-VCTs. To more broadly determine the key genes, biological processes, and pathways affected by activated PPAR*γ* in trophoblasts, in this study, we also analyzed gene expression changes in VCTs using microarray profiling, and, through various approaches, identified the enriched processes that were linked with these DEGs in RT-EVCTs and RT-VCTs. We were thus able to compare the similarities and differences between EVCTs and VCTs affected by activated PPAR*γ*. In total, there were 139 DEGs in RT-EVCTs and 197 DEGs in RT-VCTs, and these were associated with enrichment in more than 200 GO and pathway terms (Tables [Supplementary-material supplementary-material-1]). Of these terms, the most significant and relevant are depicted in the figures. The majority of the terms recovered in our analysis were consistent with reports from the existing literature. For example, the terms “long-chain fatty-acyl-CoA biosynthetic process,” “regulation of plasma lipoprotein particle levels,” “plasma lipoprotein remodeling,” and “PPAR signaling pathway” are all associated with “fatty acid transport,” which in the placenta is known to demonstrate sex-specific differences due to the PPAR*γ*-dependent response of genes involved in lipogenesis [34]. Signaling molecules and dynamic regulation of the cytoskeleton are required in trophoblast invasion [35–37], which are related to such terms as “regulation of epithelial cell migration,” “regulation of actin cytoskeleton,” and “tight junction.” Among the specific pathways highlighted, the HIF-1 signaling pathway is known to participate in PPAR*γ*-mediated placental angiogenesis [16]; the P53 signaling pathway mediates trophoblast apoptosis via ligand-specific activation of PPAR*γ* [10]; the JNK signaling pathway plays an essential role in blood-placental barrier formation [38, 39], as well as in EVCT migration and endothelial-like tube formation [40]; and the mTOR signaling pathway regulates adipogenic proteins in the placenta, with mTOR acting as a decidual nutrient sensor in histotrophic nutrition, which is crucial to embryo viability as well as early placental and fetal development [41]. Furthermore, our results were also consistent with the posttranscriptional modifications involved in placentation, with the terms “positive regulation of phosphoprotein,” “regulation of protein deacetylation,” “histone deacetylation,” and “ubiquitin-like protein conjugating enzyme activity” all known from previous reports. Indeed, different subtypes of trophoblast vary in phosphorylation status depending on the stage of placental development and differentiation. For example, EVCTs require Smad2/3 phosphorylation for differentiation while the absence of pSmad2C is necessary for VCTs [42]. Downregulation of histone deacetylase-9 can repress trophoblast migration and invasion [43], and likewise, inhibition of histone acetylation in human endometrial stromal cells limits trophoblast invasion [44]. Ubiquitination of amino acid transporters expressed specifically in the plasma membrane of the trophoblast can decrease amino acid uptake, leading to abnormal development of the placenta and restricted fetal growth [45, 46]. In addition, PPAR*γ* can be phosphorylated through activation of the downstream ERKs 1/2 or p38/c-JNK pathways [47, 48]. Rosiglitazone blocks the acetylation of lysine residues of PPAR*γ* at positions K268ac and K293ac [49]. Atypical polyubiquitination of PPAR*γ* reduces proteasomal degradation and guarantees the stabilization of PPAR*γ* [50, 51].

A major aim of this study was to compare patterns of enrichment between RT-EVCTs and RT-VCTs. During EVCT invasion, noninvasive EVCTs undergo an epithelial-mesenchymal transition to acquire the invasive phenotype [52]. Invasive EVCTs then migrate away from the placenta up to the first third of the endometrium and colonize the maternal spiral arteries. We found a comparison of these two types of trophoblasts to be particularly compelling, given the number of studies that have focused on their differences and similarities. For example, the transformation of noninvasive EVCTs into invasive EVCTs involves expression differences in adhesion molecules, which manifest themselves when EVCTs escape from the anchoring column and invade into the endometrium (decidua, spiral arteries, and myometrium) [53]. Other studies have examined differences between EVCTs and VCTs with respect to hCG secretion for the normal maintenance of pregnancy [54] and placental cytokine secretion [55]. These biological processes are apparent in the terms recovered here that were associated with “regulation of endothelial cell migration,” “embryo implantation,” “steroid hormone receptor binding,” “secretion and action,” and “preeclampsia.” The main point is that these biological processes have all been reported to be regulated by PPAR*γ*. For example, the activation of PPAR*γ* has been found to prevent the TGF-*β*-induced epithelial-mesenchymal transition via inhibition of transcription of the E-cadherin and N-cadherin promoters [56]. Furthermore, PPAR*γ* was reported to modulate basal levels of the hCG*α* and hCG*β* subunits, resulting in differences in expression between EVCTs and VCTs [57]. Additional evidence has been obtained from studies with rosiglitazone; for example, treatment with 1 *μ*M of the PPAR*γ* agonist was found to decrease and increase, respectively, the number of transcripts of TGF*β*2 and IL1*β* [32]. Such regulatory changes might be represented here by the terms “regulation of endothelial cell migration,” “Wnt signaling pathway,” “negative regulation of protein phosphorylation,” “transcription factor activity,” “PPAR signaling pathway,” and “HIF-1 signaling pathway”.

In general, our datasets revealed an abundance of biological processes or pathways affected by PPAR*γ*, many of which are consistent with previous reports. This concordance should increase confidence in our results and indicate avenues for further study. However, because this study relied on DNA microarray technology, it was inherently limited by the probe set used; it is possible that some unknown genes may not have been detected effectively and certain biological processes or pathways may have been missed. Further exploration with the application of advanced technology such as RNAseq would be helpful to identify and fill in any missing gaps in our dataset.

Finally, in order to facilitate study of the mechanisms behind the molecular interactions, we attempted to predict the protein-protein interactions between the DEGs recovered here and PPAR*γ* and RXR*α*. As was recently reviewed, the transcription exerted by the PPAR*γ* and RXR*α* complex can be modified by different types of cofactors, such as the transcriptional corepressors SMRT (silencing mediator of retinoid and thyroid hormone receptors) and NCoR (nuclear receptor corepressor), which block transactivation or the transcriptional coactivators CREB-binding protein (CBP), histone acetyltransferase p300 (p300), and PPAR-binding protein (PBP), which have the opposite effect [58]. We propose that the interaction with PPAR*γ* might affect the transcription complex formed by PPAR*γ* and RXR*α*, their cofactors or transcription partners, which could then lead to alterations in the regulation of different transcription circuits. Our results provided evidence for direct protein-protein and protein-promoter interaction with the PPAR*γ* complex. Among the proteins that appear to interact directly with PPAR*γ*, several have been experimentally verified, including ANGPTL4 [59–61], ABCG2 [62], APOB [63], CCNE1 [64], CPT1B [65], FABP4 [66–69], HMOX1 [70], and SERPINE1 [71, 72]. Many of the TF-TG interactions, which were predicted using the position weight matrix algorithm, have not been previously reported and await further verification. Our interaction matrix (Figure 7) also revealed more extensive upstream-to-downstream signaling pathways, such as the PPAR*γ*-MAPK-MMP signaling pathway. Commonly, phosphorylated PPAR*γ* stimulates the MAPK-activated pathway, leading to the activation of extracellular signal-regulated kinases (ERKs) that then induce the upregulation of matrix metalloproteinase (MMP) [73–75]. Here, only a single DEG, ATXN1, was found in both types of rosiglitazone-treated trophoblast, but with opposing responses in RT-EVCTs and RT-VCTs: this gene was upregulated in RT-EVCTs and downregulated in RT-VCTs. It has been reported that the ATXN1 protein family can regulate remodeling of the extracellular matrix [76], which indicates a potential involvement in trophoblast differentiation. However, further research is needed to determine if this gene is solely responsible for the different responses of the two distinct cell types to PPAR*γ* activation. In addition to the direct target genes predicted here, the genes in secondary relationships should be paid equal attention in terms of potential regulation by other target genes. For example, our previous research has shown the key role of LOX1, through secondary interactions, in cytotrophoblast invasion [22].

## 5. Conclusions

To our knowledge, our results reveal for the first time the widespread effects of PPAR*γ* activation in EVCTs and VCTs, highlighting extensive changes in gene expression and the biological processes and pathways affected. This study provides a broad perspective of PPAR*γ*-influenced biological processes in trophoblasts and facilitates further study, particularly into potential drug-targeted genes or pathways in the human placenta.

## Data Availability

Our microarray data have been deposited in the Gene Expression Omnibus public repository (https://www.ncbi.nlm.nih.gov/geo/); EVCT microarray data under accession number GSE28426, VCT microarray data under accession number GSE137434).

## Supplementary Materials

Supplementary Materials Differentially expressed genes (DEGs) were selected from the EVCT microarray data under accession number GSE28426 and VCT microarray data under accession number GSE137434. These genes were subsequently enriched according to the gene ontology (GO) terms. The terms of the enriched results were shown in detail in Tables S1-S8. Table S1-S4 shows the terms of GO biological process, GO cellular component, GO molecular function, and pathways of DEGs from the EVCT microarray data, respectively, while Table S5-S8 shows the terms of GO biological process, GO cellular component, GO molecular function, and pathways of DEGs from the VCT microarray data, respectively. Table S1_EVCT_DEGs_Go_Biological Process. Table S2_EVCT_DEGs_Go_Cellular Component. Table S3_EVCT_DEGs_Go_Molecular Function. Table S4_EVCT_DEGs_Pathways. Table S5_VCT_DEGs_Go_Biological Process. Table S6_VCT_DEGs_Go_Cellular Component. Table S7_VCT_DEGs_Go_Molecular Function. Table S8_VCT_DEGs_Pathways.
