# Supplementary material for: Comparative Study of PPARγ Targets in Human Extravillous and Villous Cytotrophoblasts
Source: PPAR Res. 2020 Apr 1;2020:9210748. doi: 10.1155/2020/9210748 (PMC7152979; doi:10.1155/2020/9210748)
Supplement: Supplementary Materials — Differentially expressed genes (DEGs) were selected from the EVCT microarray data under accession number GSE28426 and VCT microarray data under accession number GSE137434. These genes were subsequently enriched according to the gene ontology (GO) terms. The terms of the enriched results were shown in detail in Tables S1-S8. Table S1-S4 shows the terms of GO biological process, GO cellular component, GO molecular function, and pathways of DEGs from the EVCT microarray data, respectively, while Table S5-S8 shows the terms of GO biological process, GO cellular component, GO molecular function, and pathways of DEGs from the VCT microarray data, respectively. Table S1_EVCT_DEGs_Go_Biological Process. Table S2_EVCT_DEGs_Go_Cellular Component. Table S3_EVCT_DEGs_Go_Molecular Function. Table S4_EVCT_DEGs_Pathways. Table S5_VCT_DEGs_Go_Biological Process. Table S6_VCT_DEGs_Go_Cellular Component. Table S7_VCT_DEGs_Go_Molecular Function. Table S8_VCT_DEGs_Pathways. [file 9210748.f1.docx]

Supplementary Materials:

Differentially expressed genes (DEGs) were selected from the EVCT microarray data under accession number GSE28426 and VCT microarray data under accession number GSE137434. These genes were subsequently enriched according to the Gene Ontology (GO) terms. The terms of the enriched results were shown in detail in the Tables S1-S8. Table S1-S4 shows the terms of GO biological process, GO cellular component, GO molecular function, and pathways of DEGs from the EVCT microarray data, respectively, while Table S5-S8 shows the terms of GO biological process, GO cellular component, GO molecular function, and pathways of DEGs from the VCT microarray data, respectively.

Table S1_EVCT_DEGs_Go_Biological Process

| #term ID | term description | observed gene count | background gene count | false discovery rate | matching proteins in your network (IDs) | matching proteins in your network (labels) |
| --- | --- | --- | --- | --- | --- | --- |
| GO:0072359 | circulatory system development | 22 | 807 | 0.00013 | ENSP00000216117,ENSP00000223095,ENSP00000230658,ENSP00000231004,ENSP00000265162,ENSP00000265361,ENSP00000265729,ENSP00000276297,ENSP00000282561,ENSP00000284878,ENSP00000287820,ENSP00000301455,ENSP00000313437,ENSP00000320866,ENSP00000338272,ENSP00000341170,ENSP00000356581,ENSP00000359114,ENSP00000369055,ENSP00000371347,ENSP00000419692,ENSP00000484552 | ANGPT4,ANGPTL4,B4GALT1,CALR,COL11A1,CXADR,DLC1,ENPEP,GJA1,GJA5,GPR126,HEY1,HMOX1,ISL1,LOX,MMP19,PPARG,PTN,RXRA,SEMA3C,SERPINE1,SRI |
| GO:0007507 | heart development | 15 | 485 | 0.0022 | ENSP00000230658,ENSP00000231004,ENSP00000265361,ENSP00000265729,ENSP00000276297,ENSP00000282561,ENSP00000284878,ENSP00000287820,ENSP00000320866,ENSP00000338272,ENSP00000341170,ENSP00000356581,ENSP00000359114,ENSP00000419692,ENSP00000484552 | CALR,COL11A1,CXADR,DLC1,GJA1,GJA5,GPR126,HEY1,ISL1,LOX,PPARG,PTN,RXRA,SEMA3C,SRI |
| GO:0009653 | anatomical structure morphogenesis | 33 | 1992 | 0.0022 | ENSP00000216117,ENSP00000223095,ENSP00000230658,ENSP00000231004,ENSP00000265162,ENSP00000265361,ENSP00000276297,ENSP00000282561,ENSP00000286827,ENSP00000290271,ENSP00000295006,ENSP00000301455,ENSP00000313437,ENSP00000328998,ENSP00000337439,ENSP00000338272,ENSP00000341170,ENSP00000344741,ENSP00000356581,ENSP00000356634,ENSP00000357668,ENSP00000359114,ENSP00000362195,ENSP00000365682,ENSP00000369055,ENSP00000371347,ENSP00000371532,ENSP00000377710,ENSP00000397157,ENSP00000403954,ENSP00000419692,ENSP00000433560,ENSP00000484552 | ADAM12,ANGPT4,ANGPTL4,B4GALT1,CAPN2,COL11A1,CRYAB,CSF3R,DACT1,DLC1,ENPEP,FHL1,GJA1,GJA5,GPR126,HEY1,HMOX1,INSIG1,ISL1,LOX,MMP19,PAPPA2,PERP,PTN,RXRA,SEMA3C,SERPINE1,STC1,TENM1,TIAM1,TLE1,TRAK1,VLDLR |
| GO:0006869 | lipid transport | 11 | 272 | 0.0029 | ENSP00000237612,ENSP00000256104,ENSP00000261292,ENSP00000265641,ENSP00000268896,ENSP00000287820,ENSP00000311291,ENSP00000320291,ENSP00000371532,ENSP00000419692,ENSP00000422607 | ABCG2,ACSL1,CPT1A,FABP4,LIPG,OSBPL1A,PCTP,PPARG,RXRA,SLCO2A1,VLDLR |
| GO:0006082 | organic acid metabolic process | 20 | 959 | 0.0053 | ENSP00000169298,ENSP00000223026,ENSP00000228936,ENSP00000237612,ENSP00000265052,ENSP00000265605,ENSP00000265641,ENSP00000287820,ENSP00000308895,ENSP00000316476,ENSP00000317721,ENSP00000320291,ENSP00000348429,ENSP00000355877,ENSP00000356864,ENSP00000358831,ENSP00000369055,ENSP00000370517,ENSP00000422607,ENSP00000479870 | ABCG2,ACSL1,ACSL5,ALDH8A1,ALOX5AP,ART4,B4GALT1,CPT1A,DEGS1,ELOVL4,HYAL4,MARC1,MGLL,MGST3,OSBPL1A,PFKP,PIPOX,PPARG,SLC19A1,ST6GAL1 |
| GO:0006641 | triglyceride metabolic process | 6 | 69 | 0.0053 | ENSP00000256104,ENSP00000265052,ENSP00000265641,ENSP00000297258,ENSP00000344741,ENSP00000422607 | ACSL1,CPT1A,FABP4,FABP5,INSIG1,MGLL |
| GO:0009628 | response to abiotic stimulus | 21 | 1052 | 0.0053 | ENSP00000216117,ENSP00000257696,ENSP00000282561,ENSP00000285930,ENSP00000287820,ENSP00000290271,ENSP00000295006,ENSP00000301455,ENSP00000341170,ENSP00000347134,ENSP00000350310,ENSP00000353731,ENSP00000355512,ENSP00000356634,ENSP00000358831,ENSP00000359114,ENSP00000360025,ENSP00000371347,ENSP00000379353,ENSP00000421491,ENSP00000433560 | AKR1B1,ANGPT4,ANGPTL4,ATP2B4,CAPN2,COL11A1,CRYAB,DPP4,ELOVL4,GADD45A,GJA1,HILPDA,HMOX1,NET1,OPN3,PAPPA2,PPARG,PTN,ST8SIA1,STC1,XRCC4 |
| GO:0022603 | regulation of anatomical structure morphogenesis | 20 | 961 | 0.0053 | ENSP00000216117,ENSP00000223095,ENSP00000230658,ENSP00000265361,ENSP00000276297,ENSP00000286827,ENSP00000287820,ENSP00000300305,ENSP00000301455,ENSP00000320866,ENSP00000328998,ENSP00000337439,ENSP00000338258,ENSP00000338272,ENSP00000341170,ENSP00000347134,ENSP00000357668,ENSP00000369055,ENSP00000371347,ENSP00000419692 | ADAM12,ANGPT4,ANGPTL4,B4GALT1,CALR,CDC42EP4,DACT1,DLC1,HEY1,HMOX1,ISL1,NET1,PPARG,PTN,RUNX1,RXRA,SEMA3C,SERPINE1,TIAM1,TRAK1 |
| GO:0048518 | positive regulation of biological process | 61 | 5459 | 0.0053 | ENSP00000056233,ENSP00000216117,ENSP00000223095,ENSP00000224337,ENSP00000230658,ENSP00000256104,ENSP00000257696,ENSP00000261292,ENSP00000262065,ENSP00000262643,ENSP00000265361,ENSP00000265641,ENSP00000265729,ENSP00000266395,ENSP00000268035,ENSP00000273258,ENSP00000276297,ENSP00000282561,ENSP00000285930,ENSP00000286827,ENSP00000287820,ENSP00000290271,ENSP00000297258,ENSP00000300305,ENSP00000301455,ENSP00000310880,ENSP00000316845,ENSP00000320866,ENSP00000328998,ENSP00000337439,ENSP00000338258,ENSP00000338272,ENSP00000341071,ENSP00000341170,ENSP00000344741,ENSP00000347134,ENSP00000349003,ENSP00000350310,ENSP00000353731,ENSP00000355904,ENSP00000357668,ENSP00000360025,ENSP00000365682,ENSP00000367172,ENSP00000367794,ENSP00000369055,ENSP00000371347,ENSP00000371532,ENSP00000377710,ENSP00000377793,ENSP00000379353,ENSP00000384006,ENSP00000385510,ENSP00000397157,ENSP00000403954,ENSP00000419692,ENSP00000422607,ENSP00000427114,ENSP00000455607,ENSP00000480966,ENSP00000484552 | ACSL1,ADAM12,AKR1B1,ANGPT4,ANGPTL4,ARHGEF3,ARHGEF4,ARL6IP5,ATP2B4,B4GALT1,BLNK,CALR,CARF,CCNE1,CDC42EP4,CPT1A,DACT1,DLC1,DPP4,ESRRG,FABP4,FABP5,FHL1,FYB,GADD45A,GJA1,GJA5,GKN1,GPSM2,HEY1,HILPDA,HMOX1,IGF1R,INSIG1,ISL1,LIPG,MAL,MMD,NET1,NFE2L3,P2RY6,PDE6H,PERP,PHKG2,PPARG,PRC1,PTGER3,PTN,RUNX1,RXRA,SEMA3C,SERPINE1,SRI,SRPX,ST8SIA1,STC1,TENM1,TIAM1,TLE1,TRAK1,VLDLR |
| GO:0048646 | anatomical structure formation involved in morphogenesis | 18 | 831 | 0.0053 | ENSP00000216117,ENSP00000223095,ENSP00000230658,ENSP00000265162,ENSP00000265361,ENSP00000276297,ENSP00000295006,ENSP00000301455,ENSP00000313437,ENSP00000338272,ENSP00000356581,ENSP00000357668,ENSP00000359114,ENSP00000362195,ENSP00000369055,ENSP00000371347,ENSP00000397157,ENSP00000484552 | ADAM12,ANGPT4,ANGPTL4,B4GALT1,CAPN2,COL11A1,CSF3R,DLC1,ENPEP,GJA5,GPR126,HEY1,HMOX1,ISL1,MMP19,PERP,SEMA3C,SERPINE1 |
| GO:0050896 | response to stimulus | 78 | 7824 | 0.0053 | ENSP00000169298,ENSP00000216117,ENSP00000223095,ENSP00000224337,ENSP00000227752,ENSP00000230658,ENSP00000231004,ENSP00000237612,ENSP00000256104,ENSP00000257696,ENSP00000261292,ENSP00000262065,ENSP00000262643,ENSP00000265052,ENSP00000265361,ENSP00000265641,ENSP00000265729,ENSP00000266395,ENSP00000268035,ENSP00000273258,ENSP00000276297,ENSP00000282561,ENSP00000284878,ENSP00000285930,ENSP00000286827,ENSP00000287820,ENSP00000290271,ENSP00000295006,ENSP00000297258,ENSP00000301455,ENSP00000308895,ENSP00000313437,ENSP00000316476,ENSP00000316845,ENSP00000320866,ENSP00000332287,ENSP00000337439,ENSP00000338258,ENSP00000338272,ENSP00000341071,ENSP00000341170,ENSP00000344741,ENSP00000347134,ENSP00000349003,ENSP00000350310,ENSP00000353731,ENSP00000355512,ENSP00000355877,ENSP00000355904,ENSP00000356581,ENSP00000356634,ENSP00000356864,ENSP00000358831,ENSP00000359114,ENSP00000360002,ENSP00000360025,ENSP00000362195,ENSP00000365682,ENSP00000367794,ENSP00000369055,ENSP00000370517,ENSP00000371347,ENSP00000371532,ENSP00000379256,ENSP00000379353,ENSP00000384006,ENSP00000385510,ENSP00000397157,ENSP00000403954,ENSP00000419692,ENSP00000421491,ENSP00000422607,ENSP00000427114,ENSP00000433560,ENSP00000455607,ENSP00000478942,ENSP00000479870,ENSP00000480966 | ABCG2,ACSL1,AKR1B1,ALOX5AP,ANGPT4,ANGPTL4,ARHGEF3,ARHGEF4,ARL6IP5,ATP2B4,B4GALT1,BLNK,CALR,CAPN2,CARF,CCNE1,CDC42EP4,COL11A1,CPT1A,CRYAB,CSF3R,CXADR,DACT1,DEGS1,DLC1,DPP4,ELOVL4,ESRRG,FABP4,FABP5,FYB,GADD45A,GJA1,GPR126,GPSM2,GSTA4,HEY1,HILPDA,HMOX1,IGF1R,IL10RA,INSIG1,ISL1,LIPG,LOX,MARC1,MGLL,MGST3,MMD,MMP19,NCALD,NET1,OPN3,P2RY6,PAPPA2,PDE6H,PERP,PFKP,PHKG2,PPARG,PTGER3,PTN,RXRA,SEMA3C,SERPINE1,SLC19A1,SRI,SRPX,ST6GAL1,ST8SIA1,STC1,SYNGR1,TENM1,TIAM1,TLE1,UPK1A,VLDLR,XRCC4 |
| GO:0043436 | oxoacid metabolic process | 19 | 943 | 0.0066 | ENSP00000169298,ENSP00000223026,ENSP00000228936,ENSP00000265052,ENSP00000265605,ENSP00000265641,ENSP00000287820,ENSP00000308895,ENSP00000316476,ENSP00000317721,ENSP00000320291,ENSP00000348429,ENSP00000355877,ENSP00000356864,ENSP00000358831,ENSP00000369055,ENSP00000370517,ENSP00000422607,ENSP00000479870 | ACSL1,ACSL5,ALDH8A1,ALOX5AP,ART4,B4GALT1,CPT1A,DEGS1,ELOVL4,HYAL4,MARC1,MGLL,MGST3,OSBPL1A,PFKP,PIPOX,PPARG,SLC19A1,ST6GAL1 |
| GO:0009887 | animal organ morphogenesis | 18 | 865 | 0.0067 | ENSP00000223095,ENSP00000230658,ENSP00000265361,ENSP00000276297,ENSP00000282561,ENSP00000290271,ENSP00000338272,ENSP00000341170,ENSP00000344741,ENSP00000356581,ENSP00000356634,ENSP00000359114,ENSP00000362195,ENSP00000365682,ENSP00000377710,ENSP00000397157,ENSP00000419692,ENSP00000484552 | COL11A1,CSF3R,DLC1,FHL1,GJA1,GJA5,GPR126,HEY1,INSIG1,ISL1,PAPPA2,PERP,PTN,RXRA,SEMA3C,SERPINE1,STC1,TLE1 |
| GO:0003206 | cardiac chamber morphogenesis | 7 | 128 | 0.007 | ENSP00000230658,ENSP00000265361,ENSP00000338272,ENSP00000356581,ENSP00000359114,ENSP00000419692,ENSP00000484552 | COL11A1,GJA5,GPR126,HEY1,ISL1,RXRA,SEMA3C |
| GO:0014070 | response to organic cyclic compound | 18 | 873 | 0.007 | ENSP00000230658,ENSP00000231004,ENSP00000262643,ENSP00000265641,ENSP00000282561,ENSP00000286827,ENSP00000287820,ENSP00000290271,ENSP00000313437,ENSP00000320866,ENSP00000341170,ENSP00000344741,ENSP00000350310,ENSP00000355904,ENSP00000419692,ENSP00000422607,ENSP00000433560,ENSP00000480966 | ACSL1,ATP2B4,CALR,CCNE1,CPT1A,CRYAB,ESRRG,GJA1,INSIG1,ISL1,LOX,MMP19,P2RY6,PPARG,PTN,RXRA,STC1,TIAM1 |
| GO:0003007 | heart morphogenesis | 9 | 235 | 0.0073 | ENSP00000230658,ENSP00000265361,ENSP00000276297,ENSP00000282561,ENSP00000338272,ENSP00000356581,ENSP00000359114,ENSP00000419692,ENSP00000484552 | COL11A1,DLC1,GJA1,GJA5,GPR126,HEY1,ISL1,RXRA,SEMA3C |
| GO:0033993 | response to lipid | 17 | 825 | 0.0089 | ENSP00000223095,ENSP00000227752,ENSP00000230658,ENSP00000231004,ENSP00000262643,ENSP00000265641,ENSP00000282561,ENSP00000287820,ENSP00000290271,ENSP00000320866,ENSP00000341170,ENSP00000344741,ENSP00000355904,ENSP00000419692,ENSP00000422607,ENSP00000433560,ENSP00000480966 | ACSL1,CALR,CCNE1,CPT1A,CRYAB,ESRRG,GJA1,IL10RA,INSIG1,ISL1,LOX,P2RY6,PPARG,PTN,RXRA,SERPINE1,STC1 |
| GO:0051259 | protein complex oligomerization | 13 | 512 | 0.0089 | ENSP00000216117,ENSP00000264893,ENSP00000265641,ENSP00000268035,ENSP00000282561,ENSP00000301455,ENSP00000366694,ENSP00000369055,ENSP00000419692,ENSP00000433560,ENSP00000478942,ENSP00000479870,ENSP00000484552 | ALOX5AP,ANGPTL4,B4GALT1,CPT1A,CRYAB,GJA1,GJA5,HMOX1,IGF1R,KCTD12,RXRA,SEPT11,UPK1A |
| GO:1901342 | regulation of vasculature development | 10 | 305 | 0.0089 | ENSP00000216117,ENSP00000223095,ENSP00000230658,ENSP00000287820,ENSP00000300305,ENSP00000301455,ENSP00000338272,ENSP00000341170,ENSP00000357668,ENSP00000371347 | ADAM12,ANGPT4,ANGPTL4,HEY1,HMOX1,ISL1,PPARG,PTN,RUNX1,SERPINE1 |
| GO:0006629 | lipid metabolic process | 21 | 1192 | 0.0093 | ENSP00000256104,ENSP00000261292,ENSP00000261407,ENSP00000265052,ENSP00000265382,ENSP00000265605,ENSP00000265641,ENSP00000268896,ENSP00000285930,ENSP00000287820,ENSP00000297258,ENSP00000316476,ENSP00000320291,ENSP00000344741,ENSP00000348429,ENSP00000356864,ENSP00000358831,ENSP00000371532,ENSP00000379353,ENSP00000419692,ENSP00000422607 | ACSL1,ACSL5,AKR1B1,ALDH8A1,CPT1A,DEGS1,ELOVL4,FABP4,FABP5,INSIG1,LIPG,LPCAT3,MGLL,MGST3,OSBPL1A,PCTP,PIP5K1B,PPARG,RXRA,ST8SIA1,VLDLR |
| GO:0044281 | small molecule metabolic process | 27 | 1779 | 0.0093 | ENSP00000169298,ENSP00000223026,ENSP00000228936,ENSP00000237612,ENSP00000265052,ENSP00000265605,ENSP00000265641,ENSP00000285930,ENSP00000287820,ENSP00000297258,ENSP00000308895,ENSP00000316476,ENSP00000317721,ENSP00000320291,ENSP00000344741,ENSP00000348429,ENSP00000353770,ENSP00000355877,ENSP00000356864,ENSP00000358831,ENSP00000369055,ENSP00000370517,ENSP00000370543,ENSP00000371532,ENSP00000419692,ENSP00000422607,ENSP00000479870 | ABCG2,ACSL1,ACSL5,AKR1B1,ALDH8A1,ALOX5AP,ART4,B4GALT1,CPT1A,DEGS1,ELOVL4,FABP5,HYAL4,INSIG1,MARC1,MGLL,MGST3,OSBPL1A,PFKP,PIPOX,PPARG,RRM2,RXRA,SLC19A1,SLC5A3,ST6GAL1,VLDLR |
| GO:0048514 | blood vessel morphogenesis | 11 | 381 | 0.0095 | ENSP00000216117,ENSP00000223095,ENSP00000231004,ENSP00000265162,ENSP00000282561,ENSP00000301455,ENSP00000313437,ENSP00000338272,ENSP00000369055,ENSP00000371347,ENSP00000484552 | ANGPT4,ANGPTL4,B4GALT1,ENPEP,GJA1,GJA5,HEY1,HMOX1,LOX,MMP19,SERPINE1 |
| GO:0010646 | regulation of cell communication | 41 | 3327 | 0.0098 | ENSP00000216117,ENSP00000223095,ENSP00000224337,ENSP00000230658,ENSP00000265052,ENSP00000265382,ENSP00000265641,ENSP00000265729,ENSP00000266395,ENSP00000268035,ENSP00000273258,ENSP00000276297,ENSP00000282561,ENSP00000284878,ENSP00000285930,ENSP00000286827,ENSP00000287820,ENSP00000290271,ENSP00000297258,ENSP00000300305,ENSP00000310880,ENSP00000316845,ENSP00000320866,ENSP00000332287,ENSP00000337439,ENSP00000338272,ENSP00000341071,ENSP00000341170,ENSP00000344741,ENSP00000347134,ENSP00000348429,ENSP00000350310,ENSP00000353731,ENSP00000360025,ENSP00000365682,ENSP00000367794,ENSP00000371347,ENSP00000403954,ENSP00000418944,ENSP00000480966,ENSP00000484552 | ACSL5,AKR1B1,ANGPT4,ARHGEF3,ARHGEF4,ARL6IP5,ATP2B4,BLNK,CALR,CPT1A,CXADR,DACT1,DLC1,DPP4,FABP5,GADD45A,GJA1,GJA5,HEY1,HMOX1,IGF1R,INSIG1,ISL1,MAL,MGLL,NET1,P2RY6,PDE6H,PEG10,PIP5K1B,PPARG,PTN,RUNX1,SERPINE1,SRI,SRPX,STC1,SYNGR1,TENM1,TIAM1,TLE1 |
| GO:0001101 | response to acid chemical | 10 | 323 | 0.0102 | ENSP00000265641,ENSP00000282561,ENSP00000285930,ENSP00000287820,ENSP00000295006,ENSP00000341170,ENSP00000350310,ENSP00000419692,ENSP00000422607,ENSP00000480966 | ACSL1,AKR1B1,ATP2B4,CAPN2,CPT1A,GJA1,P2RY6,PPARG,PTN,RXRA |
| GO:0042981 | regulation of apoptotic process | 24 | 1501 | 0.0102 | ENSP00000216117,ENSP00000223095,ENSP00000230658,ENSP00000268035,ENSP00000273258,ENSP00000276297,ENSP00000285930,ENSP00000286827,ENSP00000287820,ENSP00000301455,ENSP00000310880,ENSP00000316845,ENSP00000320866,ENSP00000341071,ENSP00000341170,ENSP00000347134,ENSP00000348429,ENSP00000360025,ENSP00000365682,ENSP00000367794,ENSP00000369055,ENSP00000371347,ENSP00000397157,ENSP00000433560 | ACSL5,AKR1B1,ANGPT4,ANGPTL4,ARHGEF3,ARHGEF4,ARL6IP5,B4GALT1,CALR,CRYAB,DLC1,GADD45A,HMOX1,IGF1R,ISL1,MAL,NET1,PERP,PPARG,PTN,SERPINE1,SRPX,TIAM1,TLE1 |
| GO:0070482 | response to oxygen levels | 10 | 321 | 0.0102 | ENSP00000216117,ENSP00000257696,ENSP00000287820,ENSP00000290271,ENSP00000295006,ENSP00000301455,ENSP00000341170,ENSP00000353731,ENSP00000371347,ENSP00000433560 | ANGPT4,ANGPTL4,CAPN2,CRYAB,DPP4,HILPDA,HMOX1,PPARG,PTN,STC1 |
| GO:0001568 | blood vessel development | 12 | 464 | 0.0103 | ENSP00000216117,ENSP00000223095,ENSP00000231004,ENSP00000265162,ENSP00000265361,ENSP00000282561,ENSP00000301455,ENSP00000313437,ENSP00000338272,ENSP00000369055,ENSP00000371347,ENSP00000484552 | ANGPT4,ANGPTL4,B4GALT1,ENPEP,GJA1,GJA5,HEY1,HMOX1,LOX,MMP19,SEMA3C,SERPINE1 |
| GO:0023051 | regulation of signaling | 41 | 3360 | 0.0103 | ENSP00000216117,ENSP00000223095,ENSP00000224337,ENSP00000230658,ENSP00000265052,ENSP00000265382,ENSP00000265641,ENSP00000265729,ENSP00000266395,ENSP00000268035,ENSP00000273258,ENSP00000276297,ENSP00000282561,ENSP00000284878,ENSP00000285930,ENSP00000286827,ENSP00000287820,ENSP00000290271,ENSP00000297258,ENSP00000300305,ENSP00000310880,ENSP00000316845,ENSP00000320866,ENSP00000332287,ENSP00000337439,ENSP00000338272,ENSP00000341071,ENSP00000341170,ENSP00000344741,ENSP00000347134,ENSP00000348429,ENSP00000350310,ENSP00000353731,ENSP00000360025,ENSP00000365682,ENSP00000367794,ENSP00000371347,ENSP00000403954,ENSP00000418944,ENSP00000480966,ENSP00000484552 | ACSL5,AKR1B1,ANGPT4,ARHGEF3,ARHGEF4,ARL6IP5,ATP2B4,BLNK,CALR,CPT1A,CXADR,DACT1,DLC1,DPP4,FABP5,GADD45A,GJA1,GJA5,HEY1,HMOX1,IGF1R,INSIG1,ISL1,MAL,MGLL,NET1,P2RY6,PDE6H,PEG10,PIP5K1B,PPARG,PTN,RUNX1,SERPINE1,SRI,SRPX,STC1,SYNGR1,TENM1,TIAM1,TLE1 |
| GO:0070887 | cellular response to chemical stimulus | 35 | 2672 | 0.0103 | ENSP00000216117,ENSP00000223095,ENSP00000227752,ENSP00000230658,ENSP00000256104,ENSP00000257696,ENSP00000262643,ENSP00000265641,ENSP00000268035,ENSP00000273258,ENSP00000282561,ENSP00000284878,ENSP00000285930,ENSP00000287820,ENSP00000290271,ENSP00000295006,ENSP00000320866,ENSP00000332287,ENSP00000338258,ENSP00000341170,ENSP00000344741,ENSP00000347134,ENSP00000350310,ENSP00000355904,ENSP00000356864,ENSP00000360002,ENSP00000362195,ENSP00000370517,ENSP00000371347,ENSP00000384006,ENSP00000419692,ENSP00000421491,ENSP00000422607,ENSP00000479870,ENSP00000480966 | ACSL1,AKR1B1,ALOX5AP,ANGPT4,ARL6IP5,ATP2B4,CALR,CAPN2,CARF,CCNE1,CDC42EP4,CPT1A,CSF3R,CXADR,ESRRG,FABP4,GJA1,GSTA4,HILPDA,HMOX1,IGF1R,IL10RA,INSIG1,ISL1,MGST3,NET1,P2RY6,PFKP,PPARG,PTN,RXRA,SERPINE1,STC1,SYNGR1,XRCC4 |
| GO:0035295 | tube development | 16 | 793 | 0.0112 | ENSP00000216117,ENSP00000223095,ENSP00000231004,ENSP00000265162,ENSP00000265361,ENSP00000276297,ENSP00000282561,ENSP00000285930,ENSP00000301455,ENSP00000313437,ENSP00000337439,ENSP00000338272,ENSP00000341170,ENSP00000369055,ENSP00000371347,ENSP00000484552 | AKR1B1,ANGPT4,ANGPTL4,B4GALT1,DACT1,DLC1,ENPEP,GJA1,GJA5,HEY1,HMOX1,LOX,MMP19,PTN,SEMA3C,SERPINE1 |
| GO:0015908 | fatty acid transport | 5 | 69 | 0.0131 | ENSP00000256104,ENSP00000265641,ENSP00000287820,ENSP00000311291,ENSP00000422607 | ACSL1,CPT1A,FABP4,PPARG,SLCO2A1 |
| GO:0045765 | regulation of angiogenesis | 9 | 277 | 0.0131 | ENSP00000216117,ENSP00000223095,ENSP00000230658,ENSP00000287820,ENSP00000300305,ENSP00000301455,ENSP00000341170,ENSP00000357668,ENSP00000371347 | ADAM12,ANGPT4,ANGPTL4,HMOX1,ISL1,PPARG,PTN,RUNX1,SERPINE1 |
| GO:0045766 | positive regulation of angiogenesis | 7 | 162 | 0.0131 | ENSP00000216117,ENSP00000223095,ENSP00000230658,ENSP00000300305,ENSP00000301455,ENSP00000357668,ENSP00000371347 | ADAM12,ANGPT4,ANGPTL4,HMOX1,ISL1,RUNX1,SERPINE1 |
| GO:0071396 | cellular response to lipid | 12 | 486 | 0.0131 | ENSP00000223095,ENSP00000230658,ENSP00000262643,ENSP00000265641,ENSP00000287820,ENSP00000290271,ENSP00000320866,ENSP00000341170,ENSP00000344741,ENSP00000355904,ENSP00000419692,ENSP00000480966 | CALR,CCNE1,CPT1A,ESRRG,INSIG1,ISL1,P2RY6,PPARG,PTN,RXRA,SERPINE1,STC1 |
| GO:0001666 | response to hypoxia | 9 | 288 | 0.0134 | ENSP00000216117,ENSP00000257696,ENSP00000290271,ENSP00000295006,ENSP00000301455,ENSP00000341170,ENSP00000353731,ENSP00000371347,ENSP00000433560 | ANGPT4,ANGPTL4,CAPN2,CRYAB,DPP4,HILPDA,HMOX1,PTN,STC1 |
| GO:0003208 | cardiac ventricle morphogenesis | 5 | 72 | 0.0134 | ENSP00000230658,ENSP00000265361,ENSP00000338272,ENSP00000359114,ENSP00000419692 | COL11A1,HEY1,ISL1,RXRA,SEMA3C |
| GO:0008610 | lipid biosynthetic process | 13 | 575 | 0.0134 | ENSP00000261407,ENSP00000265052,ENSP00000265382,ENSP00000265605,ENSP00000268896,ENSP00000285930,ENSP00000297258,ENSP00000316476,ENSP00000320291,ENSP00000344741,ENSP00000358831,ENSP00000379353,ENSP00000422607 | ACSL1,AKR1B1,ALDH8A1,DEGS1,ELOVL4,FABP5,INSIG1,LPCAT3,MGLL,OSBPL1A,PCTP,PIP5K1B,ST8SIA1 |
| GO:0032879 | regulation of localization | 33 | 2524 | 0.0134 | ENSP00000216117,ENSP00000223095,ENSP00000230658,ENSP00000257696,ENSP00000261292,ENSP00000262065,ENSP00000262643,ENSP00000265361,ENSP00000265641,ENSP00000265729,ENSP00000268035,ENSP00000273258,ENSP00000276297,ENSP00000282561,ENSP00000286827,ENSP00000287820,ENSP00000290271,ENSP00000297258,ENSP00000320866,ENSP00000327611,ENSP00000341170,ENSP00000344741,ENSP00000349003,ENSP00000350310,ENSP00000353731,ENSP00000369055,ENSP00000371347,ENSP00000377710,ENSP00000385510,ENSP00000403954,ENSP00000433560,ENSP00000480966,ENSP00000484552 | ANGPT4,ARL6IP5,ATP2B4,B4GALT1,CALR,CCNE1,CPT1A,CRYAB,DLC1,DPP4,FABP5,FHL1,GJA1,GJA5,GPSM2,HILPDA,HMOX1,IGF1R,INSIG1,ISL1,KCNK12,LIPG,MMD,P2RY6,PPARG,PTGER3,PTN,SEMA3C,SERPINE1,SRI,STC1,TENM1,TIAM1 |
| GO:0048878 | chemical homeostasis | 18 | 995 | 0.0134 | ENSP00000216117,ENSP00000237612,ENSP00000256104,ENSP00000261292,ENSP00000265729,ENSP00000282561,ENSP00000285930,ENSP00000287820,ENSP00000290271,ENSP00000297205,ENSP00000297258,ENSP00000301455,ENSP00000320866,ENSP00000344741,ENSP00000350310,ENSP00000377710,ENSP00000378145,ENSP00000484552 | ABCG2,AKR1B1,ANGPTL4,ATP2B4,CALR,CCDC109B,FABP4,FABP5,FHL1,GJA1,GJA5,HMOX1,INSIG1,LIPG,PPARG,SRI,STC1,STEAP1 |
| GO:0065008 | regulation of biological quality | 42 | 3559 | 0.0134 | ENSP00000216117,ENSP00000223095,ENSP00000227752,ENSP00000230658,ENSP00000237612,ENSP00000256104,ENSP00000257696,ENSP00000261292,ENSP00000262643,ENSP00000265162,ENSP00000265605,ENSP00000265641,ENSP00000265729,ENSP00000273258,ENSP00000276297,ENSP00000282561,ENSP00000284878,ENSP00000285930,ENSP00000286827,ENSP00000287820,ENSP00000288207,ENSP00000290271,ENSP00000297205,ENSP00000297258,ENSP00000301455,ENSP00000320866,ENSP00000327611,ENSP00000332287,ENSP00000337439,ENSP00000338258,ENSP00000341170,ENSP00000344741,ENSP00000349003,ENSP00000350310,ENSP00000353731,ENSP00000377710,ENSP00000378145,ENSP00000379256,ENSP00000385510,ENSP00000403954,ENSP00000433560,ENSP00000484552 | ABCG2,AKR1B1,ALDH8A1,ANGPTL4,ARL6IP5,ATP2B4,CALR,CCDC109B,CCNB2,CCNE1,CDC42EP4,CPT1A,CRYAB,CXADR,DACT1,DLC1,DPP4,ENPEP,FABP4,FABP5,FHL1,GJA1,GJA5,GPSM2,HILPDA,HMOX1,IL10RA,INSIG1,ISL1,KCNK12,LIPG,NCALD,PPARG,PTGER3,PTN,SERPINE1,SRI,STC1,STEAP1,SYNGR1,TENM1,TIAM1 |
| GO:0071285 | cellular response to lithium ion | 3 | 14 | 0.0134 | ENSP00000256104,ENSP00000320866,ENSP00000421491 | CALR,FABP4,XRCC4 |
| GO:0001525 | angiogenesis | 9 | 297 | 0.0156 | ENSP00000216117,ENSP00000223095,ENSP00000265162,ENSP00000301455,ENSP00000313437,ENSP00000338272,ENSP00000369055,ENSP00000371347,ENSP00000484552 | ANGPT4,ANGPTL4,B4GALT1,ENPEP,GJA5,HEY1,HMOX1,MMP19,SERPINE1 |
| GO:0003231 | cardiac ventricle development | 6 | 125 | 0.0164 | ENSP00000230658,ENSP00000265361,ENSP00000338272,ENSP00000359114,ENSP00000419692,ENSP00000484552 | COL11A1,GJA5,HEY1,ISL1,RXRA,SEMA3C |
| GO:0003294 | atrial ventricular junction remodeling | 2 | 2 | 0.0164 | ENSP00000282561,ENSP00000484552 | GJA1,GJA5 |
| GO:0009725 | response to hormone | 16 | 854 | 0.0164 | ENSP00000230658,ENSP00000231004,ENSP00000262643,ENSP00000268035,ENSP00000282561,ENSP00000285930,ENSP00000287820,ENSP00000290271,ENSP00000313437,ENSP00000320866,ENSP00000341170,ENSP00000355904,ENSP00000419692,ENSP00000422607,ENSP00000433560,ENSP00000480966 | ACSL1,AKR1B1,CALR,CCNE1,CRYAB,ESRRG,GJA1,IGF1R,ISL1,LOX,MMP19,P2RY6,PPARG,PTN,RXRA,STC1 |
| GO:0010652 | positive regulation of cell communication by chemical coupling | 2 | 2 | 0.0164 | ENSP00000282561,ENSP00000484552 | GJA1,GJA5 |
| GO:0019752 | carboxylic acid metabolic process | 16 | 854 | 0.0164 | ENSP00000169298,ENSP00000228936,ENSP00000265052,ENSP00000265605,ENSP00000265641,ENSP00000287820,ENSP00000308895,ENSP00000316476,ENSP00000317721,ENSP00000320291,ENSP00000348429,ENSP00000356864,ENSP00000358831,ENSP00000370517,ENSP00000422607,ENSP00000479870 | ACSL1,ACSL5,ALDH8A1,ALOX5AP,ART4,CPT1A,DEGS1,ELOVL4,MGLL,MGST3,OSBPL1A,PFKP,PIPOX,PPARG,SLC19A1,ST6GAL1 |
| GO:0030336 | negative regulation of cell migration | 8 | 237 | 0.0164 | ENSP00000216117,ENSP00000223095,ENSP00000276297,ENSP00000287820,ENSP00000290271,ENSP00000320866,ENSP00000341170,ENSP00000371347 | ANGPT4,CALR,DLC1,HMOX1,PPARG,PTN,SERPINE1,STC1 |
| GO:0043065 | positive regulation of apoptotic process | 13 | 604 | 0.0164 | ENSP00000216117,ENSP00000273258,ENSP00000276297,ENSP00000286827,ENSP00000287820,ENSP00000310880,ENSP00000316845,ENSP00000341071,ENSP00000341170,ENSP00000347134,ENSP00000360025,ENSP00000367794,ENSP00000369055 | ARHGEF3,ARHGEF4,ARL6IP5,B4GALT1,DLC1,GADD45A,HMOX1,MAL,NET1,PPARG,PTN,SRPX,TIAM1 |
| GO:0048522 | positive regulation of cellular process | 52 | 4898 | 0.0164 | ENSP00000056233,ENSP00000216117,ENSP00000223095,ENSP00000224337,ENSP00000230658,ENSP00000257696,ENSP00000262065,ENSP00000262643,ENSP00000265361,ENSP00000265641,ENSP00000265729,ENSP00000266395,ENSP00000268035,ENSP00000273258,ENSP00000276297,ENSP00000282561,ENSP00000285930,ENSP00000286827,ENSP00000287820,ENSP00000297258,ENSP00000300305,ENSP00000310880,ENSP00000316845,ENSP00000320866,ENSP00000328998,ENSP00000337439,ENSP00000338258,ENSP00000338272,ENSP00000341071,ENSP00000341170,ENSP00000344741,ENSP00000347134,ENSP00000350310,ENSP00000353731,ENSP00000355904,ENSP00000360025,ENSP00000367172,ENSP00000367794,ENSP00000369055,ENSP00000371347,ENSP00000371532,ENSP00000377793,ENSP00000379353,ENSP00000384006,ENSP00000385510,ENSP00000397157,ENSP00000403954,ENSP00000419692,ENSP00000422607,ENSP00000455607,ENSP00000480966,ENSP00000484552 | ACSL1,AKR1B1,ANGPT4,ARHGEF3,ARHGEF4,ARL6IP5,ATP2B4,B4GALT1,BLNK,CALR,CARF,CCNE1,CDC42EP4,CPT1A,DACT1,DLC1,DPP4,ESRRG,FABP5,GADD45A,GJA1,GJA5,GKN1,GPSM2,HEY1,HILPDA,HMOX1,IGF1R,INSIG1,ISL1,MAL,MMD,NET1,NFE2L3,P2RY6,PDE6H,PERP,PHKG2,PPARG,PRC1,PTN,RUNX1,RXRA,SEMA3C,SERPINE1,SRI,SRPX,ST8SIA1,TENM1,TIAM1,TRAK1,VLDLR |
| GO:0051716 | cellular response to stimulus | 62 | 6212 | 0.0164 | ENSP00000216117,ENSP00000223095,ENSP00000224337,ENSP00000227752,ENSP00000230658,ENSP00000256104,ENSP00000257696,ENSP00000262065,ENSP00000262643,ENSP00000265361,ENSP00000265641,ENSP00000265729,ENSP00000268035,ENSP00000273258,ENSP00000276297,ENSP00000282561,ENSP00000284878,ENSP00000285930,ENSP00000286827,ENSP00000287820,ENSP00000290271,ENSP00000295006,ENSP00000316845,ENSP00000320866,ENSP00000332287,ENSP00000337439,ENSP00000338258,ENSP00000338272,ENSP00000341071,ENSP00000341170,ENSP00000344741,ENSP00000347134,ENSP00000349003,ENSP00000350310,ENSP00000355512,ENSP00000355904,ENSP00000356581,ENSP00000356864,ENSP00000358831,ENSP00000360002,ENSP00000360025,ENSP00000362195,ENSP00000365682,ENSP00000367794,ENSP00000370517,ENSP00000371347,ENSP00000371532,ENSP00000379256,ENSP00000379353,ENSP00000384006,ENSP00000385510,ENSP00000397157,ENSP00000403954,ENSP00000419692,ENSP00000421491,ENSP00000422607,ENSP00000427114,ENSP00000433560,ENSP00000455607,ENSP00000478942,ENSP00000479870,ENSP00000480966 | ACSL1,AKR1B1,ALOX5AP,ANGPT4,ARHGEF3,ARHGEF4,ARL6IP5,ATP2B4,BLNK,CALR,CAPN2,CARF,CCNE1,CDC42EP4,CPT1A,CRYAB,CSF3R,CXADR,DACT1,DLC1,ELOVL4,ESRRG,FABP4,FYB,GADD45A,GJA1,GPR126,GPSM2,GSTA4,HEY1,HILPDA,HMOX1,IGF1R,IL10RA,INSIG1,ISL1,MGST3,MMD,NCALD,NET1,OPN3,P2RY6,PERP,PFKP,PHKG2,PPARG,PTGER3,PTN,RXRA,SEMA3C,SERPINE1,SRI,SRPX,ST8SIA1,STC1,SYNGR1,TENM1,TIAM1,TLE1,UPK1A,VLDLR,XRCC4 |
| GO:0015718 | monocarboxylic acid transport | 6 | 131 | 0.0168 | ENSP00000256104,ENSP00000265641,ENSP00000287820,ENSP00000311291,ENSP00000419692,ENSP00000422607 | ACSL1,CPT1A,FABP4,PPARG,RXRA,SLCO2A1 |
| GO:0005975 | carbohydrate metabolic process | 11 | 457 | 0.0169 | ENSP00000169298,ENSP00000223026,ENSP00000265641,ENSP00000285930,ENSP00000297258,ENSP00000362643,ENSP00000369055,ENSP00000370517,ENSP00000370543,ENSP00000379353,ENSP00000455607 | AKR1B1,B4GALT1,CPT1A,FABP5,HYAL4,PFKP,PHKA1,PHKG2,SLC5A3,ST6GAL1,ST8SIA1 |
| GO:0035239 | tube morphogenesis | 13 | 615 | 0.0169 | ENSP00000216117,ENSP00000223095,ENSP00000231004,ENSP00000265162,ENSP00000276297,ENSP00000282561,ENSP00000301455,ENSP00000313437,ENSP00000337439,ENSP00000338272,ENSP00000369055,ENSP00000371347,ENSP00000484552 | ANGPT4,ANGPTL4,B4GALT1,DACT1,DLC1,ENPEP,GJA1,GJA5,HEY1,HMOX1,LOX,MMP19,SERPINE1 |
| GO:0061061 | muscle structure development | 11 | 457 | 0.0169 | ENSP00000230658,ENSP00000265729,ENSP00000284878,ENSP00000295006,ENSP00000320866,ENSP00000338272,ENSP00000357668,ENSP00000359114,ENSP00000377710,ENSP00000419692,ENSP00000433560 | ADAM12,CALR,CAPN2,COL11A1,CRYAB,CXADR,FHL1,HEY1,ISL1,RXRA,SRI |
| GO:0003158 | endothelium development | 5 | 86 | 0.0177 | ENSP00000282561,ENSP00000290271,ENSP00000338272,ENSP00000341170,ENSP00000484552 | GJA1,GJA5,HEY1,PTN,STC1 |
| GO:0046942 | carboxylic acid transport | 8 | 252 | 0.0183 | ENSP00000256104,ENSP00000265641,ENSP00000273258,ENSP00000287820,ENSP00000308895,ENSP00000311291,ENSP00000419692,ENSP00000422607 | ACSL1,ARL6IP5,CPT1A,FABP4,PPARG,RXRA,SLC19A1,SLCO2A1 |
| GO:0010643 | cell communication by chemical coupling | 2 | 3 | 0.0193 | ENSP00000282561,ENSP00000484552 | GJA1,GJA5 |
| GO:0048545 | response to steroid hormone | 9 | 324 | 0.0193 | ENSP00000230658,ENSP00000231004,ENSP00000262643,ENSP00000287820,ENSP00000290271,ENSP00000320866,ENSP00000341170,ENSP00000355904,ENSP00000419692 | CALR,CCNE1,ESRRG,ISL1,LOX,PPARG,PTN,RXRA,STC1 |
| GO:0098904 | regulation of AV node cell action potential | 2 | 3 | 0.0193 | ENSP00000284878,ENSP00000484552 | CXADR,GJA5 |
| GO:0071383 | cellular response to steroid hormone stimulus | 7 | 197 | 0.0204 | ENSP00000230658,ENSP00000262643,ENSP00000287820,ENSP00000290271,ENSP00000320866,ENSP00000355904,ENSP00000419692 | CALR,CCNE1,ESRRG,ISL1,PPARG,RXRA,STC1 |
| GO:0006811 | ion transport | 20 | 1292 | 0.0226 | ENSP00000237612,ENSP00000256104,ENSP00000265641,ENSP00000265729,ENSP00000268896,ENSP00000273258,ENSP00000282561,ENSP00000287820,ENSP00000297205,ENSP00000308895,ENSP00000311291,ENSP00000327611,ENSP00000350310,ENSP00000370543,ENSP00000378145,ENSP00000379256,ENSP00000419692,ENSP00000422607,ENSP00000480966,ENSP00000484552 | ABCG2,ACSL1,ARL6IP5,ATP2B4,CCDC109B,CPT1A,FABP4,GJA1,GJA5,KCNK12,NCALD,P2RY6,PCTP,PPARG,RXRA,SLC19A1,SLC5A3,SLCO2A1,SRI,STEAP1 |
| GO:0044262 | cellular carbohydrate metabolic process | 6 | 144 | 0.0226 | ENSP00000285930,ENSP00000362643,ENSP00000369055,ENSP00000370517,ENSP00000370543,ENSP00000455607 | AKR1B1,B4GALT1,PFKP,PHKA1,PHKG2,SLC5A3 |
| GO:0048513 | animal organ development | 35 | 2926 | 0.0228 | ENSP00000216117,ENSP00000223095,ENSP00000224337,ENSP00000230658,ENSP00000231004,ENSP00000244769,ENSP00000265162,ENSP00000265361,ENSP00000265729,ENSP00000276297,ENSP00000282561,ENSP00000284878,ENSP00000285930,ENSP00000287820,ENSP00000288207,ENSP00000290271,ENSP00000300305,ENSP00000313437,ENSP00000320866,ENSP00000338272,ENSP00000341170,ENSP00000344741,ENSP00000350310,ENSP00000356581,ENSP00000356634,ENSP00000359114,ENSP00000362195,ENSP00000365682,ENSP00000369055,ENSP00000371532,ENSP00000377710,ENSP00000397157,ENSP00000419692,ENSP00000433560,ENSP00000484552 | AKR1B1,ATP2B4,ATXN1,B4GALT1,BLNK,CALR,CCNB2,COL11A1,CRYAB,CSF3R,CXADR,DLC1,ENPEP,FHL1,GJA1,GJA5,GPR126,HEY1,HMOX1,INSIG1,ISL1,LOX,MMP19,PAPPA2,PERP,PPARG,PTN,RUNX1,RXRA,SEMA3C,SERPINE1,SRI,STC1,TLE1,VLDLR |
| GO:1903779 | regulation of cardiac conduction | 4 | 53 | 0.0237 | ENSP00000265729,ENSP00000284878,ENSP00000350310,ENSP00000484552 | ATP2B4,CXADR,GJA5,SRI |
| GO:0015711 | organic anion transport | 10 | 414 | 0.0247 | ENSP00000256104,ENSP00000265641,ENSP00000268896,ENSP00000273258,ENSP00000282561,ENSP00000287820,ENSP00000308895,ENSP00000311291,ENSP00000419692,ENSP00000422607 | ACSL1,ARL6IP5,CPT1A,FABP4,GJA1,PCTP,PPARG,RXRA,SLC19A1,SLCO2A1 |
| GO:0015909 | long-chain fatty acid transport | 4 | 54 | 0.0247 | ENSP00000256104,ENSP00000265641,ENSP00000287820,ENSP00000422607 | ACSL1,CPT1A,FABP4,PPARG |
| GO:0042221 | response to chemical | 45 | 4153 | 0.0247 | ENSP00000216117,ENSP00000223095,ENSP00000227752,ENSP00000230658,ENSP00000231004,ENSP00000237612,ENSP00000256104,ENSP00000257696,ENSP00000261292,ENSP00000262643,ENSP00000265361,ENSP00000265641,ENSP00000268035,ENSP00000273258,ENSP00000282561,ENSP00000284878,ENSP00000285930,ENSP00000286827,ENSP00000287820,ENSP00000290271,ENSP00000295006,ENSP00000308895,ENSP00000313437,ENSP00000320866,ENSP00000332287,ENSP00000338258,ENSP00000341170,ENSP00000344741,ENSP00000347134,ENSP00000350310,ENSP00000355877,ENSP00000355904,ENSP00000356864,ENSP00000360002,ENSP00000362195,ENSP00000370517,ENSP00000371347,ENSP00000371532,ENSP00000384006,ENSP00000419692,ENSP00000421491,ENSP00000422607,ENSP00000433560,ENSP00000479870,ENSP00000480966 | ABCG2,ACSL1,AKR1B1,ALOX5AP,ANGPT4,ARL6IP5,ATP2B4,CALR,CAPN2,CARF,CCNE1,CDC42EP4,CPT1A,CRYAB,CSF3R,CXADR,ESRRG,FABP4,GJA1,GSTA4,HILPDA,HMOX1,IGF1R,IL10RA,INSIG1,ISL1,LIPG,LOX,MARC1,MGST3,MMP19,NET1,P2RY6,PFKP,PPARG,PTN,RXRA,SEMA3C,SERPINE1,SLC19A1,STC1,SYNGR1,TIAM1,VLDLR,XRCC4 |
| GO:2000027 | regulation of animal organ morphogenesis | 7 | 207 | 0.0247 | ENSP00000230658,ENSP00000265361,ENSP00000286827,ENSP00000337439,ENSP00000341170,ENSP00000369055,ENSP00000419692 | B4GALT1,DACT1,ISL1,PTN,RXRA,SEMA3C,TIAM1 |
| GO:0009987 | cellular process | 116 | 14652 | 0.0258 | ENSP00000056233,ENSP00000169298,ENSP00000216117,ENSP00000216286,ENSP00000223026,ENSP00000223095,ENSP00000224337,ENSP00000227752,ENSP00000228936,ENSP00000230658,ENSP00000231004,ENSP00000237612,ENSP00000244769,ENSP00000256104,ENSP00000257696,ENSP00000261292,ENSP00000261407,ENSP00000262065,ENSP00000262643,ENSP00000264893,ENSP00000265052,ENSP00000265162,ENSP00000265361,ENSP00000265382,ENSP00000265605,ENSP00000265641,ENSP00000265729,ENSP00000268035,ENSP00000268896,ENSP00000270625,ENSP00000273258,ENSP00000276297,ENSP00000282561,ENSP00000284878,ENSP00000285930,ENSP00000286827,ENSP00000287820,ENSP00000288207,ENSP00000290271,ENSP00000295006,ENSP00000297258,ENSP00000300305,ENSP00000301455,ENSP00000308895,ENSP00000310880,ENSP00000313437,ENSP00000316476,ENSP00000316845,ENSP00000317721,ENSP00000320291,ENSP00000320866,ENSP00000325548,ENSP00000327611,ENSP00000327650,ENSP00000328998,ENSP00000332287,ENSP00000337439,ENSP00000338258,ENSP00000338272,ENSP00000340494,ENSP00000341071,ENSP00000341170,ENSP00000344741,ENSP00000347134,ENSP00000348429,ENSP00000349003,ENSP00000350310,ENSP00000353731,ENSP00000353770,ENSP00000355512,ENSP00000355877,ENSP00000355904,ENSP00000356581,ENSP00000356634,ENSP00000356864,ENSP00000357668,ENSP00000358831,ENSP00000359114,ENSP00000359890,ENSP00000360002,ENSP00000360025,ENSP00000362195,ENSP00000362643,ENSP00000363452,ENSP00000365682,ENSP00000366694,ENSP00000367794,ENSP00000369055,ENSP00000370517,ENSP00000370543,ENSP00000371347,ENSP00000371532,ENSP00000377710,ENSP00000377793,ENSP00000378145,ENSP00000379256,ENSP00000379353,ENSP00000379808,ENSP00000384006,ENSP00000385510,ENSP00000397157,ENSP00000403954,ENSP00000413625,ENSP00000418944,ENSP00000419692,ENSP00000421491,ENSP00000422607,ENSP00000427114,ENSP00000433560,ENSP00000435189,ENSP00000440674,ENSP00000455607,ENSP00000478942,ENSP00000479870,ENSP00000480966,ENSP00000484552 | ABCG2,ACSL1,ACSL5,ADAM12,AKR1B1,ALDH8A1,ALOX5AP,ANGPT4,ANGPTL4,ARHGEF3,ARHGEF4,ARL6IP5,ART4,ATF7IP2,ATP2B4,ATXN1,B4GALT1,BLNK,CALR,CAPN2,CARF,CCDC109B,CCNB2,CCNE1,CDC42EP4,CNDP2,COL11A1,CPT1A,CRYAB,CSF3R,CXADR,DACT1,DEGS1,DLC1,DPP4,ELOVL4,ENPEP,ESRRG,FABP4,FABP5,FHL1,FNBP1,FYB,GADD45A,GJA1,GJA5,GPR126,GPSM2,GSTA4,HEY1,HILPDA,HMOX1,HYAL4,IGF1R,IL10RA,INSIG1,ISL1,KCNK12,KCTD12,LIPG,LOX,LPCAT3,MAL,MAN1C1,MARC1,MBNL3,MGLL,MGST3,MMD,MMP19,NCALD,NET1,NFE2L3,NID2,OPN3,OSBPL1A,P2RY6,PAPPA2,PCTP,PEG10,PERP,PFKP,PHC1,PHKA1,PHKG2,PIP5K1B,PIPOX,PPARG,PRC1,PTGER3,PTN,RAB30,RPS11,RRM2,RUNX1,RXRA,SEMA3C,SEPT11,SERPINE1,SLC19A1,SLC5A3,SRI,SRPX,ST6GAL1,ST8SIA1,STC1,SYNGR1,TENM1,TIAM1,TLE1,TRAK1,UPK1A,VLDLR,VPS33B,XRCC4,ZNF395 |
| GO:0065009 | regulation of molecular function | 38 | 3322 | 0.0263 | ENSP00000216117,ENSP00000223095,ENSP00000230658,ENSP00000256104,ENSP00000262065,ENSP00000262643,ENSP00000265729,ENSP00000266395,ENSP00000268035,ENSP00000273258,ENSP00000276297,ENSP00000282561,ENSP00000286827,ENSP00000287820,ENSP00000290271,ENSP00000301455,ENSP00000305924,ENSP00000310880,ENSP00000316845,ENSP00000337439,ENSP00000338258,ENSP00000338272,ENSP00000341071,ENSP00000341170,ENSP00000347134,ENSP00000350310,ENSP00000360025,ENSP00000371347,ENSP00000371532,ENSP00000377710,ENSP00000378145,ENSP00000385510,ENSP00000397157,ENSP00000403954,ENSP00000421491,ENSP00000422607,ENSP00000433560,ENSP00000479870 | ACSL1,ALOX5AP,ANGPT4,ANGPTL4,ARHGEF3,ARHGEF4,ARL6IP5,ATP2B4,CCDC109B,CCNE1,CDC42EP4,CRYAB,DACT1,DLC1,FABP4,FHL1,GADD45A,GJA1,GPSM2,HEY1,HMOX1,IGF1R,ISL1,MAL,MMD,NET1,PDE6H,PERP,PPARG,PPP4R4,PTN,SERPINE1,SRI,STC1,TENM1,TIAM1,VLDLR,XRCC4 |
| GO:1901700 | response to oxygen-containing compound | 21 | 1427 | 0.0268 | ENSP00000216117,ENSP00000223095,ENSP00000227752,ENSP00000265641,ENSP00000268035,ENSP00000282561,ENSP00000285930,ENSP00000286827,ENSP00000287820,ENSP00000290271,ENSP00000295006,ENSP00000313437,ENSP00000320866,ENSP00000341170,ENSP00000344741,ENSP00000347134,ENSP00000350310,ENSP00000419692,ENSP00000422607,ENSP00000433560,ENSP00000480966 | ACSL1,AKR1B1,ATP2B4,CALR,CAPN2,CPT1A,CRYAB,GJA1,HMOX1,IGF1R,IL10RA,INSIG1,MMP19,NET1,P2RY6,PPARG,PTN,RXRA,SERPINE1,STC1,TIAM1 |
| GO:0048738 | cardiac muscle tissue development | 6 | 154 | 0.0274 | ENSP00000230658,ENSP00000284878,ENSP00000320866,ENSP00000359114,ENSP00000419692,ENSP00000484552 | CALR,COL11A1,CXADR,GJA5,ISL1,RXRA |
| GO:0071407 | cellular response to organic cyclic compound | 11 | 505 | 0.0274 | ENSP00000230658,ENSP00000262643,ENSP00000287820,ENSP00000290271,ENSP00000320866,ENSP00000341170,ENSP00000344741,ENSP00000350310,ENSP00000355904,ENSP00000419692,ENSP00000480966 | ATP2B4,CALR,CCNE1,ESRRG,INSIG1,ISL1,P2RY6,PPARG,PTN,RXRA,STC1 |
| GO:0044255 | cellular lipid metabolic process | 16 | 946 | 0.0281 | ENSP00000256104,ENSP00000261292,ENSP00000261407,ENSP00000265052,ENSP00000265382,ENSP00000265605,ENSP00000265641,ENSP00000268896,ENSP00000287820,ENSP00000297258,ENSP00000316476,ENSP00000344741,ENSP00000348429,ENSP00000358831,ENSP00000379353,ENSP00000422607 | ACSL1,ACSL5,ALDH8A1,CPT1A,DEGS1,ELOVL4,FABP4,FABP5,INSIG1,LIPG,LPCAT3,MGLL,PCTP,PIP5K1B,PPARG,ST8SIA1 |
| GO:0019433 | triglyceride catabolic process | 3 | 25 | 0.0293 | ENSP00000256104,ENSP00000265052,ENSP00000297258 | FABP4,FABP5,MGLL |
| GO:0060135 | maternal process involved in female pregnancy | 4 | 60 | 0.0306 | ENSP00000282561,ENSP00000285930,ENSP00000290271,ENSP00000419692 | AKR1B1,GJA1,RXRA,STC1 |
| GO:0071248 | cellular response to metal ion | 6 | 162 | 0.0328 | ENSP00000216117,ENSP00000256104,ENSP00000320866,ENSP00000384006,ENSP00000421491,ENSP00000479870 | ALOX5AP,CALR,CARF,FABP4,HMOX1,XRCC4 |
| GO:0006820 | anion transport | 11 | 524 | 0.0343 | ENSP00000256104,ENSP00000265641,ENSP00000268896,ENSP00000273258,ENSP00000282561,ENSP00000287820,ENSP00000308895,ENSP00000311291,ENSP00000419692,ENSP00000422607,ENSP00000480966 | ACSL1,ARL6IP5,CPT1A,FABP4,GJA1,P2RY6,PCTP,PPARG,RXRA,SLC19A1,SLCO2A1 |
| GO:0071453 | cellular response to oxygen levels | 6 | 164 | 0.0343 | ENSP00000216117,ENSP00000257696,ENSP00000287820,ENSP00000290271,ENSP00000341170,ENSP00000371347 | ANGPT4,HILPDA,HMOX1,PPARG,PTN,STC1 |
| GO:0009966 | regulation of signal transduction | 35 | 3033 | 0.0344 | ENSP00000216117,ENSP00000223095,ENSP00000224337,ENSP00000230658,ENSP00000265052,ENSP00000265382,ENSP00000265729,ENSP00000266395,ENSP00000268035,ENSP00000273258,ENSP00000276297,ENSP00000285930,ENSP00000286827,ENSP00000287820,ENSP00000290271,ENSP00000297258,ENSP00000300305,ENSP00000310880,ENSP00000316845,ENSP00000320866,ENSP00000337439,ENSP00000338272,ENSP00000341071,ENSP00000341170,ENSP00000344741,ENSP00000347134,ENSP00000348429,ENSP00000350310,ENSP00000360025,ENSP00000365682,ENSP00000367794,ENSP00000371347,ENSP00000403954,ENSP00000418944,ENSP00000480966 | ACSL5,AKR1B1,ANGPT4,ARHGEF3,ARHGEF4,ARL6IP5,ATP2B4,BLNK,CALR,DACT1,DLC1,FABP5,GADD45A,HEY1,HMOX1,IGF1R,INSIG1,ISL1,MAL,MGLL,NET1,P2RY6,PDE6H,PEG10,PIP5K1B,PPARG,PTN,RUNX1,SERPINE1,SRI,SRPX,STC1,TENM1,TIAM1,TLE1 |
| GO:0048731 | system development | 44 | 4144 | 0.0363 | ENSP00000216117,ENSP00000223095,ENSP00000224337,ENSP00000230658,ENSP00000231004,ENSP00000244769,ENSP00000262065,ENSP00000265162,ENSP00000265361,ENSP00000265729,ENSP00000276297,ENSP00000282561,ENSP00000284878,ENSP00000285930,ENSP00000286827,ENSP00000287820,ENSP00000288207,ENSP00000290271,ENSP00000300305,ENSP00000301455,ENSP00000310880,ENSP00000313437,ENSP00000320866,ENSP00000328998,ENSP00000337439,ENSP00000338272,ENSP00000341170,ENSP00000344741,ENSP00000350310,ENSP00000356581,ENSP00000356634,ENSP00000359114,ENSP00000362195,ENSP00000365682,ENSP00000369055,ENSP00000370543,ENSP00000371347,ENSP00000371532,ENSP00000377710,ENSP00000397157,ENSP00000403954,ENSP00000419692,ENSP00000433560,ENSP00000484552 | AKR1B1,ANGPT4,ANGPTL4,ATP2B4,ATXN1,B4GALT1,BLNK,CALR,CCNB2,COL11A1,CRYAB,CSF3R,CXADR,DACT1,DLC1,ENPEP,FHL1,GJA1,GJA5,GPR126,HEY1,HMOX1,INSIG1,ISL1,LOX,MAL,MMD,MMP19,PAPPA2,PERP,PPARG,PTN,RUNX1,RXRA,SEMA3C,SERPINE1,SLC5A3,SRI,STC1,TENM1,TIAM1,TLE1,TRAK1,VLDLR |
| GO:0055088 | lipid homeostasis | 5 | 111 | 0.0363 | ENSP00000256104,ENSP00000261292,ENSP00000287820,ENSP00000301455,ENSP00000344741 | ANGPTL4,FABP4,INSIG1,LIPG,PPARG |
| GO:0051270 | regulation of cellular component movement | 15 | 886 | 0.0366 | ENSP00000216117,ENSP00000223095,ENSP00000265361,ENSP00000265729,ENSP00000268035,ENSP00000276297,ENSP00000286827,ENSP00000287820,ENSP00000290271,ENSP00000320866,ENSP00000341170,ENSP00000369055,ENSP00000371347,ENSP00000480966,ENSP00000484552 | ANGPT4,B4GALT1,CALR,DLC1,GJA5,HMOX1,IGF1R,P2RY6,PPARG,PTN,SEMA3C,SERPINE1,SRI,STC1,TIAM1 |
| GO:0009755 | hormone-mediated signaling pathway | 6 | 171 | 0.0396 | ENSP00000262643,ENSP00000287820,ENSP00000320866,ENSP00000355904,ENSP00000419692,ENSP00000422607 | ACSL1,CALR,CCNE1,ESRRG,PPARG,RXRA |
| GO:0042592 | homeostatic process | 21 | 1491 | 0.0396 | ENSP00000216117,ENSP00000237612,ENSP00000256104,ENSP00000261292,ENSP00000262643,ENSP00000265729,ENSP00000282561,ENSP00000284878,ENSP00000285930,ENSP00000287820,ENSP00000288207,ENSP00000290271,ENSP00000297205,ENSP00000297258,ENSP00000301455,ENSP00000320866,ENSP00000344741,ENSP00000350310,ENSP00000377710,ENSP00000378145,ENSP00000484552 | ABCG2,AKR1B1,ANGPTL4,ATP2B4,CALR,CCDC109B,CCNB2,CCNE1,CXADR,FABP4,FABP5,FHL1,GJA1,GJA5,HMOX1,INSIG1,LIPG,PPARG,SRI,STC1,STEAP1 |
| GO:0044272 | sulfur compound biosynthetic process | 6 | 172 | 0.0403 | ENSP00000325548,ENSP00000348429,ENSP00000356864,ENSP00000360002,ENSP00000369055,ENSP00000422607 | ACSL1,ACSL5,B4GALT1,CNDP2,GSTA4,MGST3 |
| GO:0050796 | regulation of insulin secretion | 6 | 172 | 0.0403 | ENSP00000230658,ENSP00000265641,ENSP00000265729,ENSP00000282561,ENSP00000286827,ENSP00000353731 | CPT1A,DPP4,GJA1,ISL1,SRI,TIAM1 |
| GO:0042493 | response to drug | 15 | 900 | 0.0406 | ENSP00000216117,ENSP00000231004,ENSP00000237612,ENSP00000265361,ENSP00000265641,ENSP00000273258,ENSP00000282561,ENSP00000286827,ENSP00000287820,ENSP00000308895,ENSP00000320866,ENSP00000341170,ENSP00000347134,ENSP00000422607,ENSP00000433560 | ABCG2,ACSL1,ARL6IP5,CALR,CPT1A,CRYAB,GJA1,HMOX1,LOX,NET1,PPARG,PTN,SEMA3C,SLC19A1,TIAM1 |
| GO:0051101 | regulation of DNA binding | 5 | 116 | 0.0406 | ENSP00000216117,ENSP00000230658,ENSP00000265729,ENSP00000287820,ENSP00000338272 | HEY1,HMOX1,ISL1,PPARG,SRI |
| GO:0007422 | peripheral nervous system development | 4 | 68 | 0.0414 | ENSP00000230658,ENSP00000300305,ENSP00000356581,ENSP00000370543 | GPR126,ISL1,RUNX1,SLC5A3 |
| GO:0048856 | anatomical structure development | 51 | 5085 | 0.0416 | ENSP00000216117,ENSP00000223095,ENSP00000224337,ENSP00000230658,ENSP00000231004,ENSP00000244769,ENSP00000262065,ENSP00000265162,ENSP00000265361,ENSP00000265641,ENSP00000265729,ENSP00000276297,ENSP00000282561,ENSP00000284878,ENSP00000285930,ENSP00000286827,ENSP00000287820,ENSP00000288207,ENSP00000290271,ENSP00000295006,ENSP00000297258,ENSP00000300305,ENSP00000301455,ENSP00000310880,ENSP00000313437,ENSP00000320866,ENSP00000328998,ENSP00000337439,ENSP00000338272,ENSP00000341170,ENSP00000344741,ENSP00000350310,ENSP00000356581,ENSP00000356634,ENSP00000357668,ENSP00000359114,ENSP00000359890,ENSP00000362195,ENSP00000365682,ENSP00000369055,ENSP00000370543,ENSP00000371347,ENSP00000371532,ENSP00000377710,ENSP00000397157,ENSP00000403954,ENSP00000419692,ENSP00000433560,ENSP00000440674,ENSP00000478942,ENSP00000484552 | ADAM12,AKR1B1,ANGPT4,ANGPTL4,ATP2B4,ATXN1,B4GALT1,BLNK,CALR,CAPN2,CCNB2,COL11A1,CPT1A,CRYAB,CSF3R,CXADR,DACT1,DLC1,ENPEP,FABP5,FHL1,GJA1,GJA5,GPR126,HEY1,HMOX1,INSIG1,ISL1,LOX,MAL,MBNL3,MMD,MMP19,PAPPA2,PERP,PHC1,PPARG,PTN,RUNX1,RXRA,SEMA3C,SERPINE1,SLC5A3,SRI,STC1,TENM1,TIAM1,TLE1,TRAK1,UPK1A,VLDLR |
| GO:0046394 | carboxylic acid biosynthetic process | 8 | 311 | 0.0427 | ENSP00000265052,ENSP00000265605,ENSP00000316476,ENSP00000320291,ENSP00000356864,ENSP00000358831,ENSP00000370517,ENSP00000479870 | ALDH8A1,ALOX5AP,DEGS1,ELOVL4,MGLL,MGST3,OSBPL1A,PFKP |
| GO:0001893 | maternal placenta development | 3 | 31 | 0.0431 | ENSP00000282561,ENSP00000290271,ENSP00000419692 | GJA1,RXRA,STC1 |
| GO:0008217 | regulation of blood pressure | 6 | 177 | 0.0431 | ENSP00000216117,ENSP00000265162,ENSP00000282561,ENSP00000287820,ENSP00000379256,ENSP00000484552 | ENPEP,GJA1,GJA5,HMOX1,NCALD,PPARG |
| GO:0016264 | gap junction assembly | 2 | 7 | 0.0431 | ENSP00000282561,ENSP00000484552 | GJA1,GJA5 |
| GO:0051260 | protein homooligomerization | 8 | 312 | 0.0431 | ENSP00000216117,ENSP00000265641,ENSP00000301455,ENSP00000366694,ENSP00000369055,ENSP00000419692,ENSP00000433560,ENSP00000479870 | ALOX5AP,ANGPTL4,B4GALT1,CPT1A,CRYAB,HMOX1,KCTD12,RXRA |
| GO:0060373 | regulation of ventricular cardiac muscle cell membrane depolarization | 2 | 7 | 0.0431 | ENSP00000282561,ENSP00000484552 | GJA1,GJA5 |
| GO:0097006 | regulation of plasma lipoprotein particle levels | 4 | 70 | 0.0431 | ENSP00000216117,ENSP00000261292,ENSP00000261407,ENSP00000371532 | HMOX1,LIPG,LPCAT3,VLDLR |
| GO:0010565 | regulation of cellular ketone metabolic process | 5 | 122 | 0.0448 | ENSP00000265641,ENSP00000287820,ENSP00000297258,ENSP00000344741,ENSP00000350310 | ATP2B4,CPT1A,FABP5,INSIG1,PPARG |
| GO:0055114 | oxidation-reduction process | 15 | 923 | 0.0454 | ENSP00000216117,ENSP00000231004,ENSP00000265605,ENSP00000265641,ENSP00000285930,ENSP00000287820,ENSP00000297205,ENSP00000316476,ENSP00000317721,ENSP00000353770,ENSP00000355877,ENSP00000356864,ENSP00000362643,ENSP00000370517,ENSP00000455607 | AKR1B1,ALDH8A1,CPT1A,DEGS1,HMOX1,LOX,MARC1,MGST3,PFKP,PHKA1,PHKG2,PIPOX,PPARG,RRM2,STEAP1 |
| GO:0032787 | monocarboxylic acid metabolic process | 10 | 477 | 0.046 | ENSP00000265052,ENSP00000265605,ENSP00000265641,ENSP00000287820,ENSP00000316476,ENSP00000320291,ENSP00000348429,ENSP00000358831,ENSP00000370517,ENSP00000422607 | ACSL1,ACSL5,ALDH8A1,CPT1A,DEGS1,ELOVL4,MGLL,OSBPL1A,PFKP,PPARG |
| GO:0032502 | developmental process | 53 | 5401 | 0.047 | ENSP00000216117,ENSP00000223095,ENSP00000224337,ENSP00000230658,ENSP00000231004,ENSP00000244769,ENSP00000256104,ENSP00000262065,ENSP00000265162,ENSP00000265361,ENSP00000265641,ENSP00000265729,ENSP00000276297,ENSP00000282561,ENSP00000284878,ENSP00000285930,ENSP00000286827,ENSP00000287820,ENSP00000288207,ENSP00000290271,ENSP00000295006,ENSP00000297258,ENSP00000300305,ENSP00000301455,ENSP00000310880,ENSP00000313437,ENSP00000320866,ENSP00000328998,ENSP00000337439,ENSP00000338272,ENSP00000341170,ENSP00000344741,ENSP00000350310,ENSP00000356581,ENSP00000356634,ENSP00000357668,ENSP00000359114,ENSP00000359890,ENSP00000362195,ENSP00000365682,ENSP00000369055,ENSP00000370543,ENSP00000371347,ENSP00000371532,ENSP00000377710,ENSP00000397157,ENSP00000403954,ENSP00000418944,ENSP00000419692,ENSP00000433560,ENSP00000440674,ENSP00000478942,ENSP00000484552 | ADAM12,AKR1B1,ANGPT4,ANGPTL4,ATP2B4,ATXN1,B4GALT1,BLNK,CALR,CAPN2,CCNB2,COL11A1,CPT1A,CRYAB,CSF3R,CXADR,DACT1,DLC1,ENPEP,FABP4,FABP5,FHL1,GJA1,GJA5,GPR126,HEY1,HMOX1,INSIG1,ISL1,LOX,MAL,MBNL3,MMD,MMP19,PAPPA2,PEG10,PERP,PHC1,PPARG,PTN,RUNX1,RXRA,SEMA3C,SERPINE1,SLC5A3,SRI,STC1,TENM1,TIAM1,TLE1,TRAK1,UPK1A,VLDLR |
| GO:0006575 | cellular modified amino acid metabolic process | 6 | 185 | 0.0484 | ENSP00000261407,ENSP00000265641,ENSP00000308895,ENSP00000317721,ENSP00000325548,ENSP00000360002 | CNDP2,CPT1A,GSTA4,LPCAT3,PIPOX,SLC19A1 |
| GO:0035357 | peroxisome proliferator activated receptor signaling pathway | 2 | 8 | 0.0484 | ENSP00000287820,ENSP00000419692 | PPARG,RXRA |
| GO:0046486 | glycerolipid metabolic process | 9 | 401 | 0.0484 | ENSP00000256104,ENSP00000261407,ENSP00000265052,ENSP00000265382,ENSP00000265641,ENSP00000268896,ENSP00000297258,ENSP00000344741,ENSP00000422607 | ACSL1,CPT1A,FABP4,FABP5,INSIG1,LPCAT3,MGLL,PCTP,PIP5K1B |
| GO:0060371 | regulation of atrial cardiac muscle cell membrane depolarization | 2 | 8 | 0.0484 | ENSP00000282561,ENSP00000484552 | GJA1,GJA5 |
| GO:0060411 | cardiac septum morphogenesis | 4 | 74 | 0.0484 | ENSP00000230658,ENSP00000265361,ENSP00000338272,ENSP00000484552 | GJA5,HEY1,ISL1,SEMA3C |
| GO:0048583 | regulation of response to stimulus | 41 | 3882 | 0.0492 | ENSP00000216117,ENSP00000223095,ENSP00000224337,ENSP00000230658,ENSP00000256104,ENSP00000265052,ENSP00000265382,ENSP00000265729,ENSP00000266395,ENSP00000268035,ENSP00000273258,ENSP00000276297,ENSP00000282561,ENSP00000284878,ENSP00000285930,ENSP00000286827,ENSP00000287820,ENSP00000290271,ENSP00000297258,ENSP00000300305,ENSP00000310880,ENSP00000316845,ENSP00000320866,ENSP00000337439,ENSP00000338272,ENSP00000341071,ENSP00000341170,ENSP00000344741,ENSP00000347134,ENSP00000348429,ENSP00000349003,ENSP00000350310,ENSP00000360025,ENSP00000365682,ENSP00000367794,ENSP00000371347,ENSP00000403954,ENSP00000418944,ENSP00000427114,ENSP00000433560,ENSP00000480966 | ACSL5,AKR1B1,ANGPT4,ARHGEF3,ARHGEF4,ARL6IP5,ATP2B4,BLNK,CALR,CRYAB,CXADR,DACT1,DLC1,FABP4,FABP5,FYB,GADD45A,GJA1,HEY1,HMOX1,IGF1R,INSIG1,ISL1,MAL,MGLL,NET1,P2RY6,PDE6H,PEG10,PIP5K1B,PPARG,PTGER3,PTN,RUNX1,SERPINE1,SRI,SRPX,STC1,TENM1,TIAM1,TLE1 |
| GO:0051239 | regulation of multicellular organismal process | 32 | 2788 | 0.0492 | ENSP00000216117,ENSP00000223095,ENSP00000230658,ENSP00000257696,ENSP00000261292,ENSP00000262065,ENSP00000265052,ENSP00000265361,ENSP00000265729,ENSP00000282561,ENSP00000284878,ENSP00000286827,ENSP00000287820,ENSP00000290271,ENSP00000297258,ENSP00000300305,ENSP00000301455,ENSP00000320866,ENSP00000328998,ENSP00000337439,ENSP00000338272,ENSP00000341170,ENSP00000349003,ENSP00000350310,ENSP00000357668,ENSP00000362195,ENSP00000369055,ENSP00000370543,ENSP00000371347,ENSP00000371532,ENSP00000419692,ENSP00000484552 | ADAM12,ANGPT4,ANGPTL4,ATP2B4,B4GALT1,CALR,CSF3R,CXADR,DACT1,FABP5,GJA1,GJA5,HEY1,HILPDA,HMOX1,ISL1,LIPG,MGLL,MMD,PPARG,PTGER3,PTN,RUNX1,RXRA,SEMA3C,SERPINE1,SLC5A3,SRI,STC1,TIAM1,TRAK1,VLDLR |
| GO:0051240 | positive regulation of multicellular organismal process | 21 | 1551 | 0.0494 | ENSP00000216117,ENSP00000223095,ENSP00000230658,ENSP00000257696,ENSP00000261292,ENSP00000262065,ENSP00000265361,ENSP00000282561,ENSP00000286827,ENSP00000287820,ENSP00000300305,ENSP00000301455,ENSP00000320866,ENSP00000328998,ENSP00000341170,ENSP00000349003,ENSP00000357668,ENSP00000369055,ENSP00000371347,ENSP00000371532,ENSP00000484552 | ADAM12,ANGPT4,ANGPTL4,B4GALT1,CALR,GJA1,GJA5,HILPDA,HMOX1,ISL1,LIPG,MMD,PPARG,PTGER3,PTN,RUNX1,SEMA3C,SERPINE1,TIAM1,TRAK1,VLDLR |

Table S2_EVCT_DEGs_Go_Cellular Component

| #term ID | term description | observed gene count | background gene count | false discovery rate | matching proteins in your network (IDs) | matching proteins in your network (labels) |
| --- | --- | --- | --- | --- | --- | --- |
| GO:0044444 | cytoplasmic part | 89 | 9377 | 0.0053 | ENSP00000169298,ENSP00000216117,ENSP00000223095,ENSP00000223167,ENSP00000224337,ENSP00000237612,ENSP00000244769,ENSP00000256104,ENSP00000257696,ENSP00000261292,ENSP00000261407,ENSP00000262065,ENSP00000262643,ENSP00000265052,ENSP00000265162,ENSP00000265382,ENSP00000265605,ENSP00000265641,ENSP00000265729,ENSP00000268896,ENSP00000270625,ENSP00000273258,ENSP00000276297,ENSP00000282561,ENSP00000284878,ENSP00000285930,ENSP00000286827,ENSP00000287820,ENSP00000288207,ENSP00000295006,ENSP00000297205,ENSP00000297258,ENSP00000300305,ENSP00000305924,ENSP00000310880,ENSP00000316476,ENSP00000316845,ENSP00000317721,ENSP00000320291,ENSP00000320866,ENSP00000325548,ENSP00000327650,ENSP00000328998,ENSP00000332287,ENSP00000337439,ENSP00000338258,ENSP00000339754,ENSP00000340494,ENSP00000341071,ENSP00000341170,ENSP00000344741,ENSP00000347134,ENSP00000348429,ENSP00000350310,ENSP00000352111,ENSP00000353731,ENSP00000353770,ENSP00000355877,ENSP00000356634,ENSP00000356864,ENSP00000358831,ENSP00000359114,ENSP00000360002,ENSP00000362643,ENSP00000363452,ENSP00000365682,ENSP00000367794,ENSP00000369055,ENSP00000370517,ENSP00000371532,ENSP00000377710,ENSP00000377793,ENSP00000378145,ENSP00000379256,ENSP00000379353,ENSP00000385510,ENSP00000397157,ENSP00000403954,ENSP00000413625,ENSP00000418944,ENSP00000421491,ENSP00000422607,ENSP00000426120,ENSP00000427114,ENSP00000433560,ENSP00000435189,ENSP00000455607,ENSP00000478942,ENSP00000479870 | ABCG2,ACSL1,ACSL5,AKR1B1,ALDH8A1,ALOX5AP,ARHGEF3,ARHGEF4,ARL4C,ARL6IP5,ATP2B4,ATXN1,B4GALT1,BLNK,CALR,CAPN2,CCDC109B,CCNB2,CCNE1,CDC42EP4,CNDP2,COL11A1,CPT1A,CRYAB,CXADR,DACT1,DEGS1,DLC1,DPP4,ELOVL4,ENPEP,FABP4,FABP5,FHL1,FNBP1,FYB,GJA1,GOLGA8A,GPSM2,GRAMD3,GSTA4,HILPDA,HMOX1,INSIG1,LIPG,LPCAT3,MAL,MAN1C1,MARC1,MGLL,MGST3,MMD,MYL10,NCALD,NET1,OSBPL1A,PAPPA2,PCTP,PEG10,PERP,PFKP,PHKA1,PHKG2,PIP5K1B,PIPOX,PPARG,PPP4R4,PRC1,PTN,RAB30,RPS11,RRM2,RUNX1,SERPINE1,SRI,SRPX,ST6GAL1,ST8SIA1,STEAP1,SYNGR1,TENM1,TIAM1,TLE1,TRAK1,UPK1A,VLDLR,VPS33B,XRCC4,ZNF395 |
| GO:0005737 | cytoplasm | 98 | 11238 | 0.0075 | ENSP00000056233,ENSP00000169298,ENSP00000216117,ENSP00000223095,ENSP00000223167,ENSP00000224337,ENSP00000230658,ENSP00000237612,ENSP00000244769,ENSP00000256104,ENSP00000257696,ENSP00000261292,ENSP00000261407,ENSP00000262065,ENSP00000262643,ENSP00000264893,ENSP00000265052,ENSP00000265162,ENSP00000265382,ENSP00000265605,ENSP00000265641,ENSP00000265729,ENSP00000268896,ENSP00000270625,ENSP00000273258,ENSP00000276297,ENSP00000282561,ENSP00000284878,ENSP00000285930,ENSP00000286827,ENSP00000287820,ENSP00000288207,ENSP00000290271,ENSP00000295006,ENSP00000297205,ENSP00000297258,ENSP00000300305,ENSP00000305924,ENSP00000310880,ENSP00000316476,ENSP00000316845,ENSP00000317721,ENSP00000320291,ENSP00000320866,ENSP00000325548,ENSP00000327650,ENSP00000328998,ENSP00000332287,ENSP00000337439,ENSP00000338258,ENSP00000338272,ENSP00000339754,ENSP00000340494,ENSP00000341071,ENSP00000341170,ENSP00000344741,ENSP00000347134,ENSP00000348429,ENSP00000350310,ENSP00000352111,ENSP00000353731,ENSP00000353770,ENSP00000355877,ENSP00000356634,ENSP00000356864,ENSP00000358831,ENSP00000359114,ENSP00000359890,ENSP00000360002,ENSP00000360025,ENSP00000362643,ENSP00000363452,ENSP00000365682,ENSP00000367794,ENSP00000369055,ENSP00000370517,ENSP00000371532,ENSP00000377710,ENSP00000377793,ENSP00000378145,ENSP00000378996,ENSP00000379256,ENSP00000379353,ENSP00000385510,ENSP00000397157,ENSP00000403954,ENSP00000413625,ENSP00000418944,ENSP00000421491,ENSP00000422607,ENSP00000426120,ENSP00000427114,ENSP00000433560,ENSP00000435189,ENSP00000455607,ENSP00000464780,ENSP00000478942,ENSP00000479870 | ABCG2,ACSL1,ACSL5,AKR1B1,ALDH8A1,ALOX5AP,ARHGEF3,ARHGEF4,ARL4C,ARL6IP5,ATP2B4,ATXN1,B4GALT1,BLNK,CALR,CAPN2,CCDC109B,CCNB2,CCNE1,CDC42EP4,CNDP2,COL11A1,CPT1A,CRYAB,CXADR,DACT1,DEGS1,DLC1,DPP4,ELOVL4,ENPEP,FABP4,FABP5,FHL1,FNBP1,FYB,GADD45A,GJA1,GOLGA8A,GPSM2,GRAMD3,GSTA4,HEY1,HILPDA,HMOX1,INSIG1,ISL1,LIPG,LPCAT3,MAL,MAN1C1,MARC1,MBNL3,MGLL,MGST3,MMD,MYL10,NCALD,NET1,NFE2L3,NREP,OSBPL1A,PAPPA2,PCTP,PEG10,PERP,PFKP,PHKA1,PHKG2,PIP5K1B,PIPOX,PPARG,PPP4R4,PRC1,PTN,RAB30,RPS11,RRM2,RUNX1,SEPT11,SERPINE1,SRI,SRPX,ST6GAL1,ST8SIA1,STC1,STEAP1,SYNGR1,TENM1,TIAM1,TLE1,TPGS2,TRAK1,UPK1A,VLDLR,VPS33B,XRCC4,ZNF395 |
| GO:0005886 | plasma membrane | 57 | 5159 | 0.0075 | ENSP00000216117,ENSP00000216286,ENSP00000223095,ENSP00000224337,ENSP00000227752,ENSP00000228936,ENSP00000237612,ENSP00000262065,ENSP00000265052,ENSP00000265162,ENSP00000265729,ENSP00000268035,ENSP00000273258,ENSP00000276297,ENSP00000282561,ENSP00000284878,ENSP00000286827,ENSP00000290271,ENSP00000295006,ENSP00000297205,ENSP00000297258,ENSP00000308895,ENSP00000310880,ENSP00000311291,ENSP00000316476,ENSP00000316845,ENSP00000320866,ENSP00000327611,ENSP00000332287,ENSP00000338258,ENSP00000339754,ENSP00000347134,ENSP00000349003,ENSP00000350310,ENSP00000353731,ENSP00000355512,ENSP00000356581,ENSP00000356634,ENSP00000357668,ENSP00000362195,ENSP00000362643,ENSP00000366694,ENSP00000369055,ENSP00000370543,ENSP00000371532,ENSP00000377710,ENSP00000385510,ENSP00000397157,ENSP00000403954,ENSP00000413625,ENSP00000422607,ENSP00000427114,ENSP00000433560,ENSP00000460602,ENSP00000478942,ENSP00000480966,ENSP00000484552 | ABCG2,ACSL1,ADAM12,ARHGEF4,ARL4C,ARL6IP5,ART4,ATP2B4,B4GALT1,BLNK,CALR,CAPN2,CDC42EP4,CRYAB,CSF3R,CXADR,DEGS1,DLC1,DPP4,ENPEP,ERVMER34-1,FABP5,FHL1,FNBP1,FYB,GJA1,GJA5,GPR126,GPSM2,HMOX1,IGF1R,IL10RA,KCNK12,KCTD12,MAL,MGLL,MMD,NET1,NID2,OPN3,P2RY6,PAPPA2,PERP,PHKA1,PTGER3,SERPINE1,SLC19A1,SLC5A3,SLCO2A1,SRI,STC1,STEAP1,SYNGR1,TENM1,TIAM1,UPK1A,VLDLR |
| GO:0016020 | membrane | 80 | 8420 | 0.0075 | ENSP00000169298,ENSP00000216117,ENSP00000216286,ENSP00000223026,ENSP00000223095,ENSP00000224337,ENSP00000227752,ENSP00000228936,ENSP00000237612,ENSP00000257696,ENSP00000261407,ENSP00000262065,ENSP00000265052,ENSP00000265162,ENSP00000265382,ENSP00000265641,ENSP00000265729,ENSP00000268035,ENSP00000273258,ENSP00000276297,ENSP00000282561,ENSP00000284878,ENSP00000286827,ENSP00000288207,ENSP00000290271,ENSP00000295006,ENSP00000297205,ENSP00000297258,ENSP00000308895,ENSP00000310880,ENSP00000311291,ENSP00000316476,ENSP00000316845,ENSP00000320866,ENSP00000327611,ENSP00000327650,ENSP00000332287,ENSP00000338258,ENSP00000339754,ENSP00000341170,ENSP00000344741,ENSP00000347134,ENSP00000348429,ENSP00000349003,ENSP00000350310,ENSP00000352111,ENSP00000353731,ENSP00000355512,ENSP00000355877,ENSP00000356581,ENSP00000356634,ENSP00000356864,ENSP00000357668,ENSP00000358831,ENSP00000362195,ENSP00000362643,ENSP00000363452,ENSP00000366694,ENSP00000367794,ENSP00000369055,ENSP00000370543,ENSP00000371532,ENSP00000377710,ENSP00000378145,ENSP00000379256,ENSP00000379353,ENSP00000382767,ENSP00000385510,ENSP00000397157,ENSP00000403954,ENSP00000413625,ENSP00000422607,ENSP00000427114,ENSP00000433560,ENSP00000435189,ENSP00000460602,ENSP00000478942,ENSP00000479870,ENSP00000480966,ENSP00000484552 | ABCG2,ACSL1,ACSL5,ADAM12,ALOX5AP,ARHGEF4,ARL4C,ARL6IP5,ART4,ATP2B4,B4GALT1,BLNK,CALR,CAPN2,CCDC109B,CCNB2,CDC42EP4,CPT1A,CRYAB,CSF3R,CXADR,DEGS1,DLC1,DPP4,ELOVL4,ENPEP,ERVMER34-1,FABP5,FHL1,FNBP1,FYB,GJA1,GJA5,GOLGA8A,GPR126,GPSM2,HILPDA,HMOX1,HYAL4,IGF1R,IL10RA,INSIG1,KCNK12,KCTD12,LPCAT3,MAL,MAN1C1,MARC1,MGLL,MGST3,MMD,NCALD,NET1,NID2,OPN3,P2RY6,PAPPA2,PERP,PHKA1,PIP5K1B,PTGER3,PTN,RAB30,SEL1L3,SERPINE1,SLC19A1,SLC5A3,SLCO2A1,SRI,SRPX,ST6GAL1,ST8SIA1,STC1,STEAP1,SYNGR1,TENM1,TIAM1,UPK1A,VLDLR,VPS33B |
| GO:0016324 | apical plasma membrane | 10 | 307 | 0.0075 | ENSP00000237612,ENSP00000265162,ENSP00000282561,ENSP00000290271,ENSP00000308895,ENSP00000310880,ENSP00000353731,ENSP00000356634,ENSP00000478942,ENSP00000480966 | ABCG2,DPP4,ENPEP,GJA1,MAL,P2RY6,PAPPA2,SLC19A1,STC1,UPK1A |
| GO:0044459 | plasma membrane part | 36 | 2651 | 0.0075 | ENSP00000216117,ENSP00000237612,ENSP00000262065,ENSP00000265162,ENSP00000265729,ENSP00000268035,ENSP00000276297,ENSP00000282561,ENSP00000284878,ENSP00000286827,ENSP00000290271,ENSP00000297205,ENSP00000297258,ENSP00000308895,ENSP00000310880,ENSP00000311291,ENSP00000316476,ENSP00000316845,ENSP00000320866,ENSP00000327611,ENSP00000349003,ENSP00000350310,ENSP00000353731,ENSP00000355512,ENSP00000356634,ENSP00000362195,ENSP00000366694,ENSP00000369055,ENSP00000370543,ENSP00000385510,ENSP00000397157,ENSP00000403954,ENSP00000433560,ENSP00000478942,ENSP00000480966,ENSP00000484552 | ABCG2,ARHGEF4,ATP2B4,B4GALT1,CALR,CRYAB,CSF3R,CXADR,DEGS1,DLC1,DPP4,ENPEP,FABP5,GJA1,GJA5,GPSM2,HMOX1,IGF1R,KCNK12,KCTD12,MAL,MMD,OPN3,P2RY6,PAPPA2,PERP,PTGER3,SLC19A1,SLC5A3,SLCO2A1,SRI,STC1,STEAP1,TENM1,TIAM1,UPK1A |
| GO:0098590 | plasma membrane region | 20 | 1061 | 0.0075 | ENSP00000216117,ENSP00000237612,ENSP00000265162,ENSP00000276297,ENSP00000282561,ENSP00000284878,ENSP00000286827,ENSP00000290271,ENSP00000297258,ENSP00000308895,ENSP00000310880,ENSP00000316845,ENSP00000350310,ENSP00000353731,ENSP00000356634,ENSP00000366694,ENSP00000369055,ENSP00000433560,ENSP00000478942,ENSP00000480966 | ABCG2,ARHGEF4,ATP2B4,B4GALT1,CRYAB,CXADR,DLC1,DPP4,ENPEP,FABP5,GJA1,HMOX1,KCTD12,MAL,P2RY6,PAPPA2,SLC19A1,STC1,TIAM1,UPK1A |
| GO:0044425 | membrane part | 64 | 6517 | 0.0158 | ENSP00000169298,ENSP00000216117,ENSP00000223026,ENSP00000227752,ENSP00000228936,ENSP00000237612,ENSP00000257696,ENSP00000261407,ENSP00000262065,ENSP00000265052,ENSP00000265162,ENSP00000265641,ENSP00000265729,ENSP00000268035,ENSP00000273258,ENSP00000276297,ENSP00000282561,ENSP00000284878,ENSP00000286827,ENSP00000290271,ENSP00000295006,ENSP00000297205,ENSP00000297258,ENSP00000308895,ENSP00000310880,ENSP00000311291,ENSP00000316476,ENSP00000316845,ENSP00000320866,ENSP00000327611,ENSP00000332287,ENSP00000344741,ENSP00000348429,ENSP00000349003,ENSP00000350310,ENSP00000353731,ENSP00000355512,ENSP00000355877,ENSP00000356581,ENSP00000356634,ENSP00000356864,ENSP00000357668,ENSP00000358831,ENSP00000362195,ENSP00000363452,ENSP00000366694,ENSP00000369055,ENSP00000370543,ENSP00000371532,ENSP00000378145,ENSP00000379256,ENSP00000379353,ENSP00000382767,ENSP00000385510,ENSP00000397157,ENSP00000403954,ENSP00000413625,ENSP00000422607,ENSP00000433560,ENSP00000460602,ENSP00000478942,ENSP00000479870,ENSP00000480966,ENSP00000484552 | ABCG2,ACSL1,ACSL5,ADAM12,ALOX5AP,ARHGEF4,ARL6IP5,ART4,ATP2B4,B4GALT1,CALR,CAPN2,CCDC109B,CPT1A,CRYAB,CSF3R,CXADR,DEGS1,DLC1,DPP4,ELOVL4,ENPEP,ERVMER34-1,FABP5,FNBP1,GJA1,GJA5,GPR126,GPSM2,HILPDA,HMOX1,HYAL4,IGF1R,IL10RA,INSIG1,KCNK12,KCTD12,LPCAT3,MAL,MAN1C1,MARC1,MGLL,MGST3,MMD,NCALD,OPN3,P2RY6,PAPPA2,PERP,PTGER3,SEL1L3,SLC19A1,SLC5A3,SLCO2A1,SRI,ST6GAL1,ST8SIA1,STC1,STEAP1,SYNGR1,TENM1,TIAM1,UPK1A,VLDLR |
| GO:0005911 | cell-cell junction | 10 | 402 | 0.0195 | ENSP00000282561,ENSP00000284878,ENSP00000285930,ENSP00000286827,ENSP00000297205,ENSP00000338258,ENSP00000353731,ENSP00000369055,ENSP00000397157,ENSP00000484552 | AKR1B1,B4GALT1,CDC42EP4,CXADR,DPP4,GJA1,GJA5,PERP,STEAP1,TIAM1 |
| GO:0030054 | cell junction | 17 | 1006 | 0.0199 | ENSP00000264893,ENSP00000276297,ENSP00000282561,ENSP00000284878,ENSP00000285930,ENSP00000286827,ENSP00000297205,ENSP00000297258,ENSP00000332287,ENSP00000337439,ENSP00000338258,ENSP00000353731,ENSP00000366694,ENSP00000369055,ENSP00000397157,ENSP00000427114,ENSP00000484552 | AKR1B1,B4GALT1,CDC42EP4,CXADR,DACT1,DLC1,DPP4,FABP5,FYB,GJA1,GJA5,KCTD12,PERP,SEPT11,STEAP1,SYNGR1,TIAM1 |
| GO:0005964 | phosphorylase kinase complex | 2 | 5 | 0.0296 | ENSP00000362643,ENSP00000455607 | PHKA1,PHKG2 |
| GO:0012505 | endomembrane system | 46 | 4347 | 0.0296 | ENSP00000169298,ENSP00000216117,ENSP00000223095,ENSP00000257696,ENSP00000261292,ENSP00000261407,ENSP00000262065,ENSP00000265052,ENSP00000265382,ENSP00000265729,ENSP00000273258,ENSP00000282561,ENSP00000284878,ENSP00000295006,ENSP00000297205,ENSP00000297258,ENSP00000310880,ENSP00000316476,ENSP00000320291,ENSP00000320866,ENSP00000327650,ENSP00000328998,ENSP00000332287,ENSP00000338258,ENSP00000341170,ENSP00000344741,ENSP00000348429,ENSP00000349003,ENSP00000352111,ENSP00000356864,ENSP00000358831,ENSP00000359114,ENSP00000363452,ENSP00000367794,ENSP00000369055,ENSP00000371532,ENSP00000379256,ENSP00000379353,ENSP00000397157,ENSP00000403954,ENSP00000413625,ENSP00000422607,ENSP00000433560,ENSP00000435189,ENSP00000478942,ENSP00000479870 | ACSL1,ACSL5,ALOX5AP,ARL6IP5,B4GALT1,CALR,CAPN2,CDC42EP4,COL11A1,CRYAB,CXADR,DEGS1,ELOVL4,FABP5,FNBP1,GJA1,GOLGA8A,HILPDA,HMOX1,INSIG1,LIPG,LPCAT3,MAL,MAN1C1,MGLL,MGST3,MMD,NCALD,OSBPL1A,PERP,PIP5K1B,PTGER3,PTN,RAB30,SERPINE1,SRI,SRPX,ST6GAL1,ST8SIA1,STEAP1,SYNGR1,TENM1,TRAK1,UPK1A,VLDLR,VPS33B |
| GO:0044464 | cell part | 124 | 16244 | 0.0296 | ENSP00000056233,ENSP00000169298,ENSP00000216117,ENSP00000216286,ENSP00000223026,ENSP00000223095,ENSP00000223167,ENSP00000224337,ENSP00000227752,ENSP00000228936,ENSP00000230658,ENSP00000231004,ENSP00000237612,ENSP00000244769,ENSP00000256104,ENSP00000257696,ENSP00000258243,ENSP00000261292,ENSP00000261407,ENSP00000262065,ENSP00000262643,ENSP00000264893,ENSP00000265052,ENSP00000265162,ENSP00000265382,ENSP00000265605,ENSP00000265641,ENSP00000265729,ENSP00000268035,ENSP00000268896,ENSP00000270625,ENSP00000273258,ENSP00000276297,ENSP00000282561,ENSP00000284878,ENSP00000285930,ENSP00000286827,ENSP00000287820,ENSP00000288207,ENSP00000290271,ENSP00000295006,ENSP00000297205,ENSP00000297258,ENSP00000300305,ENSP00000305924,ENSP00000308895,ENSP00000310880,ENSP00000311291,ENSP00000316476,ENSP00000316845,ENSP00000317721,ENSP00000320291,ENSP00000320866,ENSP00000325548,ENSP00000327611,ENSP00000327650,ENSP00000328998,ENSP00000332287,ENSP00000337439,ENSP00000338258,ENSP00000338272,ENSP00000339754,ENSP00000340494,ENSP00000341071,ENSP00000341170,ENSP00000344741,ENSP00000347134,ENSP00000348429,ENSP00000349003,ENSP00000350310,ENSP00000352111,ENSP00000353731,ENSP00000353770,ENSP00000355512,ENSP00000355877,ENSP00000355904,ENSP00000356581,ENSP00000356634,ENSP00000356864,ENSP00000357668,ENSP00000358831,ENSP00000359114,ENSP00000359890,ENSP00000360002,ENSP00000360025,ENSP00000362195,ENSP00000362643,ENSP00000363452,ENSP00000365682,ENSP00000366694,ENSP00000367794,ENSP00000369055,ENSP00000370517,ENSP00000370543,ENSP00000371532,ENSP00000377710,ENSP00000377793,ENSP00000378145,ENSP00000378996,ENSP00000379256,ENSP00000379353,ENSP00000379808,ENSP00000382767,ENSP00000384006,ENSP00000385510,ENSP00000397157,ENSP00000403954,ENSP00000413625,ENSP00000418944,ENSP00000419692,ENSP00000421491,ENSP00000422607,ENSP00000426120,ENSP00000427114,ENSP00000433560,ENSP00000435189,ENSP00000440674,ENSP00000455607,ENSP00000460602,ENSP00000464780,ENSP00000478942,ENSP00000479870,ENSP00000480966,ENSP00000484552 | ABCG2,ACSL1,ACSL5,ADAM12,AKR1B1,ALDH8A1,ALOX5AP,ARHGEF3,ARHGEF4,ARL4C,ARL6IP5,ART4,ATF7IP2,ATP2B4,ATXN1,B4GALT1,BLNK,CALR,CAPN2,CARF,CCDC109B,CCNB2,CCNE1,CDC42EP4,CNDP2,COL11A1,CPT1A,CRYAB,CSF3R,CXADR,DACT1,DEGS1,DLC1,DPP4,ELOVL4,ENPEP,ERVMER34-1,ESRRG,FABP4,FABP5,FHL1,FNBP1,FYB,GADD45A,GJA1,GJA5,GOLGA8A,GPR126,GPSM2,GRAMD3,GSTA4,HEY1,HILPDA,HMOX1,HYAL4,IGF1R,IL10RA,INSIG1,ISL1,KCNK12,KCTD12,LIPG,LOX,LPCAT3,MAL,MAN1C1,MARC1,MBNL3,MGLL,MGST3,MMD,MYL10,NCALD,NET1,NFE2L3,NID2,NREP,OPN3,OSBPL1A,P2RY6,PAPPA2,PCTP,PEG10,PERP,PFKP,PHC1,PHKA1,PHKG2,PIP5K1B,PIPOX,PPARG,PPP4R4,PRC1,PTGER3,PTN,RAB30,RPS11,RRM2,RUNX1,RXRA,SEL1L3,SEPT11,SERPINE1,SLC19A1,SLC5A3,SLCO2A1,SRI,SRPX,ST6GAL1,ST8SIA1,STC1,STEAP1,SYNGR1,TENM1,TIAM1,TLE1,TPGS2,TRAK1,UPK1A,URB2,VLDLR,VPS33B,XRCC4,ZNF395 |
| GO:0098805 | whole membrane | 22 | 1554 | 0.0296 | ENSP00000216117,ENSP00000262065,ENSP00000265641,ENSP00000276297,ENSP00000282561,ENSP00000284878,ENSP00000295006,ENSP00000297205,ENSP00000297258,ENSP00000310880,ENSP00000316476,ENSP00000320866,ENSP00000327650,ENSP00000332287,ENSP00000348429,ENSP00000350310,ENSP00000353731,ENSP00000355877,ENSP00000369055,ENSP00000371532,ENSP00000379256,ENSP00000422607 | ACSL1,ACSL5,ATP2B4,B4GALT1,CALR,CAPN2,CPT1A,CXADR,DEGS1,DLC1,DPP4,FABP5,GJA1,HMOX1,MAL,MARC1,MMD,NCALD,STEAP1,SYNGR1,VLDLR,VPS33B |
| GO:0005829 | cytosol | 50 | 4958 | 0.0299 | ENSP00000216117,ENSP00000223167,ENSP00000224337,ENSP00000244769,ENSP00000256104,ENSP00000257696,ENSP00000262643,ENSP00000265052,ENSP00000265382,ENSP00000265605,ENSP00000265729,ENSP00000268896,ENSP00000270625,ENSP00000276297,ENSP00000282561,ENSP00000285930,ENSP00000286827,ENSP00000287820,ENSP00000288207,ENSP00000295006,ENSP00000297258,ENSP00000300305,ENSP00000305924,ENSP00000316845,ENSP00000317721,ENSP00000320291,ENSP00000320866,ENSP00000325548,ENSP00000337439,ENSP00000339754,ENSP00000340494,ENSP00000341071,ENSP00000347134,ENSP00000352111,ENSP00000353770,ENSP00000356634,ENSP00000360002,ENSP00000362643,ENSP00000365682,ENSP00000370517,ENSP00000377710,ENSP00000377793,ENSP00000379256,ENSP00000385510,ENSP00000413625,ENSP00000418944,ENSP00000421491,ENSP00000427114,ENSP00000433560,ENSP00000455607 | AKR1B1,ALDH8A1,ARHGEF3,ARHGEF4,ARL4C,ATXN1,BLNK,CALR,CAPN2,CCNB2,CCNE1,CNDP2,CRYAB,DACT1,DLC1,FABP4,FABP5,FHL1,FNBP1,FYB,GJA1,GOLGA8A,GPSM2,GSTA4,HILPDA,HMOX1,MGLL,MYL10,NCALD,NET1,OSBPL1A,PAPPA2,PCTP,PEG10,PFKP,PHKA1,PHKG2,PIP5K1B,PIPOX,PPARG,PPP4R4,PRC1,RPS11,RRM2,RUNX1,SRI,TIAM1,TLE1,XRCC4,ZNF395 |
| GO:0044291 | cell-cell contact zone | 4 | 67 | 0.0299 | ENSP00000282561,ENSP00000284878,ENSP00000286827,ENSP00000484552 | CXADR,GJA1,GJA5,TIAM1 |
| GO:0045121 | membrane raft | 8 | 300 | 0.0299 | ENSP00000216117,ENSP00000276297,ENSP00000282561,ENSP00000284878,ENSP00000295006,ENSP00000310880,ENSP00000350310,ENSP00000353731 | ATP2B4,CAPN2,CXADR,DLC1,DPP4,GJA1,HMOX1,MAL |

Table S3_EVCT_DEGs_Go_Molecular Function

| #term ID | term description | observed gene count | background gene count | false discovery rate | matching proteins in your network (IDs) | matching proteins in your network (labels) |
| --- | --- | --- | --- | --- | --- | --- |
| GO:0071253 | connexin binding | 3 | 6 | 0.0135 | ENSP00000282561,ENSP00000284878,ENSP00000484552 | CXADR,GJA1,GJA5 |
| GO:0005504 | fatty acid binding | 4 | 27 | 0.015 | ENSP00000256104,ENSP00000287820,ENSP00000297258,ENSP00000479870 | ALOX5AP,FABP4,FABP5,PPARG |
| GO:0005515 | protein binding | 67 | 6605 | 0.015 | ENSP00000216117,ENSP00000223095,ENSP00000224337,ENSP00000227752,ENSP00000230658,ENSP00000237612,ENSP00000244769,ENSP00000256104,ENSP00000257696,ENSP00000262643,ENSP00000265052,ENSP00000265361,ENSP00000265641,ENSP00000265729,ENSP00000268035,ENSP00000276297,ENSP00000282561,ENSP00000284878,ENSP00000286827,ENSP00000287820,ENSP00000290271,ENSP00000295006,ENSP00000297258,ENSP00000300305,ENSP00000301455,ENSP00000316845,ENSP00000317721,ENSP00000320866,ENSP00000328998,ENSP00000337439,ENSP00000338258,ENSP00000338272,ENSP00000339754,ENSP00000341071,ENSP00000341170,ENSP00000347134,ENSP00000350310,ENSP00000353731,ENSP00000355904,ENSP00000356581,ENSP00000357668,ENSP00000359114,ENSP00000360002,ENSP00000360025,ENSP00000362195,ENSP00000362643,ENSP00000365682,ENSP00000366694,ENSP00000369055,ENSP00000370517,ENSP00000371347,ENSP00000371532,ENSP00000377710,ENSP00000377793,ENSP00000379256,ENSP00000385510,ENSP00000403954,ENSP00000413625,ENSP00000419692,ENSP00000421491,ENSP00000426120,ENSP00000427114,ENSP00000433560,ENSP00000455607,ENSP00000478942,ENSP00000479870,ENSP00000484552 | ABCG2,ADAM12,ALOX5AP,ANGPT4,ANGPTL4,ARHGEF3,ARHGEF4,ARL4C,ATP2B4,ATXN1,B4GALT1,BLNK,CALR,CAPN2,CCNE1,CDC42EP4,COL11A1,CPT1A,CRYAB,CSF3R,CXADR,DACT1,DLC1,DPP4,ESRRG,FABP4,FABP5,FHL1,FNBP1,FYB,GADD45A,GJA1,GJA5,GPR126,GPSM2,GRAMD3,GSTA4,HEY1,HILPDA,HMOX1,IGF1R,IL10RA,ISL1,KCTD12,MGLL,NCALD,NET1,PFKP,PHKA1,PHKG2,PIPOX,PPARG,PRC1,PTN,RUNX1,RXRA,SEMA3C,SERPINE1,SRI,STC1,TENM1,TIAM1,TLE1,TRAK1,UPK1A,VLDLR,XRCC4 |
| GO:0033293 | monocarboxylic acid binding | 5 | 57 | 0.015 | ENSP00000256104,ENSP00000287820,ENSP00000297258,ENSP00000419692,ENSP00000479870 | ALOX5AP,FABP4,FABP5,PPARG,RXRA |
| GO:0036041 | long-chain fatty acid binding | 3 | 13 | 0.015 | ENSP00000256104,ENSP00000287820,ENSP00000479870 | ALOX5AP,FABP4,PPARG |
| GO:0042802 | identical protein binding | 27 | 1754 | 0.015 | ENSP00000216117,ENSP00000237612,ENSP00000244769,ENSP00000265052,ENSP00000265641,ENSP00000265729,ENSP00000268035,ENSP00000284878,ENSP00000287820,ENSP00000297258,ENSP00000300305,ENSP00000301455,ENSP00000353731,ENSP00000360002,ENSP00000360025,ENSP00000365682,ENSP00000366694,ENSP00000369055,ENSP00000370517,ENSP00000377793,ENSP00000385510,ENSP00000403954,ENSP00000413625,ENSP00000421491,ENSP00000426120,ENSP00000433560,ENSP00000478942 | ABCG2,ANGPTL4,ATXN1,B4GALT1,CPT1A,CRYAB,CXADR,DPP4,FABP5,FNBP1,GADD45A,GPSM2,GRAMD3,GSTA4,HMOX1,IGF1R,KCTD12,MGLL,PFKP,PPARG,PRC1,RUNX1,SRI,TENM1,TLE1,UPK1A,XRCC4 |
| GO:0086075 | gap junction channel activity involved in cardiac conduction electrical coupling | 2 | 3 | 0.0349 | ENSP00000282561,ENSP00000484552 | GJA1,GJA5 |
| GO:0004689 | phosphorylase kinase activity | 2 | 5 | 0.045 | ENSP00000362643,ENSP00000455607 | PHKA1,PHKG2 |
| GO:0005102 | signaling receptor binding | 22 | 1513 | 0.045 | ENSP00000223095,ENSP00000224337,ENSP00000230658,ENSP00000256104,ENSP00000257696,ENSP00000262643,ENSP00000265361,ENSP00000265729,ENSP00000268035,ENSP00000282561,ENSP00000284878,ENSP00000286827,ENSP00000287820,ENSP00000290271,ENSP00000317721,ENSP00000320866,ENSP00000328998,ENSP00000341170,ENSP00000353731,ENSP00000371347,ENSP00000419692,ENSP00000427114 | ANGPT4,BLNK,CALR,CCNE1,CXADR,DPP4,FABP4,FYB,GJA1,HILPDA,IGF1R,ISL1,PIPOX,PPARG,PTN,RXRA,SEMA3C,SERPINE1,SRI,STC1,TIAM1,TRAK1 |
| GO:0008289 | lipid binding | 13 | 673 | 0.045 | ENSP00000256104,ENSP00000268896,ENSP00000276297,ENSP00000286827,ENSP00000287820,ENSP00000297258,ENSP00000310880,ENSP00000320291,ENSP00000355904,ENSP00000413625,ENSP00000419692,ENSP00000427114,ENSP00000479870 | ALOX5AP,DLC1,ESRRG,FABP4,FABP5,FNBP1,FYB,MAL,OSBPL1A,PCTP,PPARG,RXRA,TIAM1 |
| GO:0016922 | nuclear receptor binding | 5 | 111 | 0.045 | ENSP00000230658,ENSP00000262643,ENSP00000287820,ENSP00000320866,ENSP00000419692 | CALR,CCNE1,ISL1,PPARG,RXRA |
| GO:0050544 | arachidonic acid binding | 2 | 5 | 0.045 | ENSP00000287820,ENSP00000479870 | ALOX5AP,PPARG |
| GO:0050692 | DBD domain binding | 2 | 4 | 0.045 | ENSP00000287820,ENSP00000419692 | PPARG,RXRA |
| GO:0050693 | LBD domain binding | 2 | 6 | 0.045 | ENSP00000287820,ENSP00000419692 | PPARG,RXRA |
| GO:0051427 | hormone receptor binding | 6 | 174 | 0.045 | ENSP00000230658,ENSP00000256104,ENSP00000262643,ENSP00000287820,ENSP00000320866,ENSP00000419692 | CALR,CCNE1,FABP4,ISL1,PPARG,RXRA |
| GO:1904399 | heparan sulfate binding | 2 | 4 | 0.045 | ENSP00000341170,ENSP00000359114 | COL11A1,PTN |

Table S4_EVCT_DEGs_Pathways

| #term ID | term description | observed gene count | background gene count | false discovery rate | matching proteins in your network (IDs) | matching proteins in your network (labels) |
| --- | --- | --- | --- | --- | --- | --- |
| hsa03320 | PPAR signaling pathway | 8 | 72 | 1.15E-05 | ENSP00000256104,ENSP00000265641,ENSP00000287820,ENSP00000297258,ENSP00000301455,ENSP00000348429,ENSP00000419692,ENSP00000422607 | ACSL1,ACSL5,ANGPTL4,CPT1A,FABP4,FABP5,PPARG,RXRA |
| hsa04115 | p53 signaling pathway | 6 | 68 | 0.0009 | ENSP00000223095,ENSP00000262643,ENSP00000288207,ENSP00000353770,ENSP00000360025,ENSP00000397157 | CCNB2,CCNE1,GADD45A,PERP,RRM2,SERPINE1 |
| hsa04216 | Ferroptosis | 4 | 40 | 0.0121 | ENSP00000216117,ENSP00000261407,ENSP00000348429,ENSP00000422607 | ACSL1,ACSL5,HMOX1,LPCAT3 |
| hsa05200 | Pathways in cancer | 12 | 515 | 0.0121 | ENSP00000216117,ENSP00000262643,ENSP00000268035,ENSP00000287820,ENSP00000300305,ENSP00000338272,ENSP00000349003,ENSP00000356864,ENSP00000360002,ENSP00000360025,ENSP00000362195,ENSP00000419692 | CCNE1,CSF3R,GADD45A,GSTA4,HEY1,HMOX1,IGF1R,MGST3,PPARG,PTGER3,RUNX1,RXRA |
| hsa00052 | Galactose metabolism | 3 | 31 | 0.0499 | ENSP00000285930,ENSP00000369055,ENSP00000370517 | AKR1B1,B4GALT1,PFKP |
| hsa04920 | Adipocytokine signaling pathway | 4 | 69 | 0.0499 | ENSP00000265641,ENSP00000348429,ENSP00000419692,ENSP00000422607 | ACSL1,ACSL5,CPT1A,RXRA |
| HSA-556833 | Metabolism of lipids | 19 | 721 | 0.00028 | ENSP00000256104,ENSP00000257696,ENSP00000261407,ENSP00000265052,ENSP00000265382,ENSP00000265641,ENSP00000268896,ENSP00000285930,ENSP00000287820,ENSP00000297258,ENSP00000301455,ENSP00000316476,ENSP00000320291,ENSP00000344741,ENSP00000348429,ENSP00000358831,ENSP00000419692,ENSP00000422607,ENSP00000479870 | ACSL1,ACSL5,AKR1B1,ALOX5AP,ANGPTL4,CPT1A,DEGS1,ELOVL4,FABP4,FABP5,HILPDA,INSIG1,LPCAT3,MGLL,OSBPL1A,PCTP,PIP5K1B,PPARG,RXRA |
| HSA-1430728 | Metabolism | 33 | 2032 | 0.00049 | ENSP00000216117,ENSP00000237612,ENSP00000256104,ENSP00000257696,ENSP00000261407,ENSP00000265052,ENSP00000265382,ENSP00000265641,ENSP00000268896,ENSP00000270625,ENSP00000285930,ENSP00000287820,ENSP00000297258,ENSP00000301455,ENSP00000308895,ENSP00000316476,ENSP00000317721,ENSP00000320291,ENSP00000325548,ENSP00000344741,ENSP00000348429,ENSP00000353770,ENSP00000355877,ENSP00000356864,ENSP00000358831,ENSP00000360002,ENSP00000362643,ENSP00000369055,ENSP00000370517,ENSP00000419692,ENSP00000422607,ENSP00000455607,ENSP00000479870 | ABCG2,ACSL1,ACSL5,AKR1B1,ALOX5AP,ANGPTL4,B4GALT1,CNDP2,CPT1A,DEGS1,ELOVL4,FABP4,FABP5,GSTA4,HILPDA,HMOX1,INSIG1,LPCAT3,MARC1,MGLL,MGST3,OSBPL1A,PCTP,PFKP,PHKA1,PHKG2,PIP5K1B,PIPOX,PPARG,RPS11,RRM2,RXRA,SLC19A1 |
| HSA-5362517 | Signaling by Retinoic Acid | 4 | 43 | 0.0322 | ENSP00000265605,ENSP00000265641,ENSP00000297258,ENSP00000419692 | ALDH8A1,CPT1A,FABP5,RXRA |
| HSA-8978868 | Fatty acid metabolism | 7 | 171 | 0.0322 | ENSP00000265641,ENSP00000268896,ENSP00000348429,ENSP00000358831,ENSP00000419692,ENSP00000422607,ENSP00000479870 | ACSL1,ACSL5,ALOX5AP,CPT1A,ELOVL4,PCTP,RXRA |

Table S5_VCT_DEGs_Go_Biological Process

| #term ID | term description | observed gene count | background gene count | false discovery rate | matching proteins in your network (IDs) | matching proteins in your network (labels) |
| --- | --- | --- | --- | --- | --- | --- |
| GO:0000184 | nuclear-transcribed mRNA catabolic process, nonsense-mediated decay | 19 | 118 | 1.64E-12 | ENSP00000222247,ENSP00000262584,ENSP00000270625,ENSP00000309334,ENSP00000325074,ENSP00000339795,ENSP00000341885,ENSP00000346015,ENSP00000359345,ENSP00000373715,ENSP00000378163,ENSP00000379506,ENSP00000379888,ENSP00000385958,ENSP00000400467,ENSP00000403172,ENSP00000419117,ENSP00000435777,ENSP00000464813 | DCP2,PPP2R2A,RPL14,RPL15,RPL18A,RPL27,RPL27A,RPL34,RPL35A,RPL5,RPL6,RPL7,RPL8,RPL9,RPS11,RPS13,RPS14,RPS2,RPS8 |
| GO:0000956 | nuclear-transcribed mRNA catabolic process | 22 | 191 | 2.12E-12 | ENSP00000221233,ENSP00000222247,ENSP00000262584,ENSP00000265564,ENSP00000270625,ENSP00000309334,ENSP00000325074,ENSP00000339795,ENSP00000341885,ENSP00000346015,ENSP00000359345,ENSP00000361433,ENSP00000373715,ENSP00000378163,ENSP00000379506,ENSP00000379888,ENSP00000385958,ENSP00000400467,ENSP00000403172,ENSP00000419117,ENSP00000435777,ENSP00000464813 | DCP2,EXOSC2,EXOSC5,EXOSC7,PPP2R2A,RPL14,RPL15,RPL18A,RPL27,RPL27A,RPL34,RPL35A,RPL5,RPL6,RPL7,RPL8,RPL9,RPS11,RPS13,RPS14,RPS2,RPS8 |
| GO:0006614 | SRP-dependent cotranslational protein targeting to membrane | 17 | 92 | 2.65E-12 | ENSP00000222247,ENSP00000262584,ENSP00000270625,ENSP00000309334,ENSP00000339795,ENSP00000341885,ENSP00000346015,ENSP00000359345,ENSP00000378163,ENSP00000379506,ENSP00000379888,ENSP00000385958,ENSP00000400467,ENSP00000403172,ENSP00000419117,ENSP00000435777,ENSP00000464813 | RPL14,RPL15,RPL18A,RPL27,RPL27A,RPL34,RPL35A,RPL5,RPL6,RPL7,RPL8,RPL9,RPS11,RPS13,RPS14,RPS2,RPS8 |
| GO:0006413 | translational initiation | 19 | 142 | 5.22E-12 | ENSP00000222247,ENSP00000223273,ENSP00000262584,ENSP00000270625,ENSP00000309334,ENSP00000339795,ENSP00000341885,ENSP00000346015,ENSP00000359345,ENSP00000368927,ENSP00000378163,ENSP00000379506,ENSP00000379888,ENSP00000385958,ENSP00000400467,ENSP00000403172,ENSP00000419117,ENSP00000435777,ENSP00000464813 | EIF1AX,RPL14,RPL15,RPL18A,RPL27,RPL27A,RPL34,RPL35A,RPL5,RPL6,RPL7,RPL8,RPL9,RPS11,RPS13,RPS14,RPS2,RPS8,YAE1D1 |
| GO:0090150 | establishment of protein localization to membrane | 20 | 217 | 3.39E-10 | ENSP00000222247,ENSP00000262584,ENSP00000270625,ENSP00000309334,ENSP00000327716,ENSP00000339795,ENSP00000341885,ENSP00000346015,ENSP00000359345,ENSP00000365606,ENSP00000378163,ENSP00000379506,ENSP00000379888,ENSP00000385958,ENSP00000400467,ENSP00000403172,ENSP00000419117,ENSP00000435777,ENSP00000464813,ENSP00000479322 | CLIP1,GRIPAP1,RPL14,RPL15,RPL18A,RPL27,RPL27A,RPL34,RPL35A,RPL5,RPL6,RPL7,RPL8,RPL9,RPS11,RPS13,RPS14,RPS2,RPS8,WRB |
| GO:0043043 | peptide biosynthetic process | 25 | 386 | 6.73E-10 | ENSP00000210060,ENSP00000222247,ENSP00000223273,ENSP00000252602,ENSP00000253099,ENSP00000262584,ENSP00000270625,ENSP00000309334,ENSP00000316335,ENSP00000339795,ENSP00000341885,ENSP00000346015,ENSP00000359345,ENSP00000367038,ENSP00000368927,ENSP00000378163,ENSP00000379506,ENSP00000379888,ENSP00000385958,ENSP00000400467,ENSP00000403172,ENSP00000419117,ENSP00000435777,ENSP00000464813,ENSP00000483552 | DHPS,EIF1AX,FARS2,FURIN,MRPL34,MRPL4,RPL14,RPL15,RPL18A,RPL27,RPL27A,RPL34,RPL35A,RPL5,RPL6,RPL7,RPL8,RPL9,RPS11,RPS13,RPS14,RPS2,RPS8,RRBP1,YAE1D1 |
| GO:0034655 | nucleobase-containing compound catabolic process | 25 | 394 | 9.53E-10 | ENSP00000221233,ENSP00000222247,ENSP00000229239,ENSP00000262584,ENSP00000265564,ENSP00000270625,ENSP00000309334,ENSP00000325074,ENSP00000339795,ENSP00000341885,ENSP00000346015,ENSP00000359345,ENSP00000361433,ENSP00000364145,ENSP00000364212,ENSP00000373715,ENSP00000378163,ENSP00000379506,ENSP00000379888,ENSP00000385958,ENSP00000400467,ENSP00000403172,ENSP00000419117,ENSP00000435777,ENSP00000464813 | CDA,DCP2,EXOSC2,EXOSC5,EXOSC7,GAPDH,PFKFB1,PPP2R2A,RPL14,RPL15,RPL18A,RPL27,RPL27A,RPL34,RPL35A,RPL5,RPL6,RPL7,RPL8,RPL9,RPS11,RPS13,RPS14,RPS2,RPS8 |
| GO:0006412 | translation | 24 | 362 | 1.04E-09 | ENSP00000210060,ENSP00000222247,ENSP00000223273,ENSP00000252602,ENSP00000253099,ENSP00000262584,ENSP00000270625,ENSP00000309334,ENSP00000316335,ENSP00000339795,ENSP00000341885,ENSP00000346015,ENSP00000359345,ENSP00000367038,ENSP00000368927,ENSP00000378163,ENSP00000379506,ENSP00000379888,ENSP00000385958,ENSP00000400467,ENSP00000403172,ENSP00000419117,ENSP00000435777,ENSP00000464813 | DHPS,EIF1AX,FARS2,MRPL34,MRPL4,RPL14,RPL15,RPL18A,RPL27,RPL27A,RPL34,RPL35A,RPL5,RPL6,RPL7,RPL8,RPL9,RPS11,RPS13,RPS14,RPS2,RPS8,RRBP1,YAE1D1 |
| GO:0019439 | aromatic compound catabolic process | 26 | 453 | 2.52E-09 | ENSP00000221233,ENSP00000222247,ENSP00000229239,ENSP00000262584,ENSP00000265564,ENSP00000270625,ENSP00000309334,ENSP00000325074,ENSP00000339795,ENSP00000341885,ENSP00000346015,ENSP00000359345,ENSP00000361433,ENSP00000364145,ENSP00000364212,ENSP00000373715,ENSP00000378163,ENSP00000379506,ENSP00000379888,ENSP00000385080,ENSP00000385958,ENSP00000400467,ENSP00000403172,ENSP00000419117,ENSP00000435777,ENSP00000464813 | CDA,DCP2,EXOSC2,EXOSC5,EXOSC7,FAH,GAPDH,PFKFB1,PPP2R2A,RPL14,RPL15,RPL18A,RPL27,RPL27A,RPL34,RPL35A,RPL5,RPL6,RPL7,RPL8,RPL9,RPS11,RPS13,RPS14,RPS2,RPS8 |
| GO:0016071 | mRNA metabolic process | 31 | 667 | 3.78E-09 | ENSP00000221233,ENSP00000222247,ENSP00000230859,ENSP00000236273,ENSP00000240185,ENSP00000262584,ENSP00000265564,ENSP00000270625,ENSP00000300291,ENSP00000309334,ENSP00000325074,ENSP00000339795,ENSP00000341885,ENSP00000346015,ENSP00000354518,ENSP00000359345,ENSP00000361433,ENSP00000373715,ENSP00000378163,ENSP00000379506,ENSP00000379888,ENSP00000385958,ENSP00000394085,ENSP00000400467,ENSP00000403172,ENSP00000418563,ENSP00000419117,ENSP00000435777,ENSP00000443926,ENSP00000452123,ENSP00000464813 | CELF2,DCP2,EXOSC2,EXOSC5,EXOSC7,JMJD6,NUDT21,PAPD7,PPP2R2A,RPL14,RPL15,RPL18A,RPL27,RPL27A,RPL34,RPL35A,RPL5,RPL6,RPL7,RPL8,RPL9,RPS11,RPS13,RPS14,RPS2,RPS8,SRSF5,SYF2,TARDBP,U2SURP,ZNF830 |
| GO:1901361 | organic cyclic compound catabolic process | 26 | 484 | 8.07E-09 | ENSP00000221233,ENSP00000222247,ENSP00000229239,ENSP00000262584,ENSP00000265564,ENSP00000270625,ENSP00000309334,ENSP00000325074,ENSP00000339795,ENSP00000341885,ENSP00000346015,ENSP00000359345,ENSP00000361433,ENSP00000364145,ENSP00000364212,ENSP00000373715,ENSP00000378163,ENSP00000379506,ENSP00000379888,ENSP00000385080,ENSP00000385958,ENSP00000400467,ENSP00000403172,ENSP00000419117,ENSP00000435777,ENSP00000464813 | CDA,DCP2,EXOSC2,EXOSC5,EXOSC7,FAH,GAPDH,PFKFB1,PPP2R2A,RPL14,RPL15,RPL18A,RPL27,RPL27A,RPL34,RPL35A,RPL5,RPL6,RPL7,RPL8,RPL9,RPS11,RPS13,RPS14,RPS2,RPS8 |
| GO:0006518 | peptide metabolic process | 26 | 497 | 1.33E-08 | ENSP00000210060,ENSP00000222247,ENSP00000223273,ENSP00000225719,ENSP00000252602,ENSP00000253099,ENSP00000262584,ENSP00000270625,ENSP00000309334,ENSP00000316335,ENSP00000339795,ENSP00000341885,ENSP00000346015,ENSP00000359345,ENSP00000367038,ENSP00000368927,ENSP00000378163,ENSP00000379506,ENSP00000379888,ENSP00000385958,ENSP00000400467,ENSP00000403172,ENSP00000419117,ENSP00000435777,ENSP00000464813,ENSP00000483552 | CPD,DHPS,EIF1AX,FARS2,FURIN,MRPL34,MRPL4,RPL14,RPL15,RPL18A,RPL27,RPL27A,RPL34,RPL35A,RPL5,RPL6,RPL7,RPL8,RPL9,RPS11,RPS13,RPS14,RPS2,RPS8,RRBP1,YAE1D1 |
| GO:0022613 | ribonucleoprotein complex biogenesis | 23 | 409 | 4.46E-08 | ENSP00000221233,ENSP00000223273,ENSP00000265564,ENSP00000273317,ENSP00000300291,ENSP00000339795,ENSP00000341885,ENSP00000345917,ENSP00000348965,ENSP00000359345,ENSP00000359859,ENSP00000361433,ENSP00000366843,ENSP00000378163,ENSP00000379506,ENSP00000379888,ENSP00000385958,ENSP00000403172,ENSP00000419117,ENSP00000423067,ENSP00000443926,ENSP00000452123,ENSP00000464813 | ATXN2,CELF2,DYNC1H1,EXOSC2,EXOSC5,EXOSC7,LIMD1,LYAR,MTG2,NUDT21,RPL14,RPL27,RPL34,RPL35A,RPL5,RPL6,RPL7,RPS14,RPS2,RPS8,SRSF5,WDR36,YAE1D1 |
| GO:0044265 | cellular macromolecule catabolic process | 32 | 842 | 1.64E-07 | ENSP00000044462,ENSP00000216455,ENSP00000221233,ENSP00000222247,ENSP00000261427,ENSP00000262584,ENSP00000265564,ENSP00000270625,ENSP00000274306,ENSP00000309334,ENSP00000325074,ENSP00000339795,ENSP00000341885,ENSP00000346015,ENSP00000359345,ENSP00000361433,ENSP00000361540,ENSP00000373715,ENSP00000378163,ENSP00000379506,ENSP00000379888,ENSP00000380178,ENSP00000385958,ENSP00000400467,ENSP00000401802,ENSP00000403172,ENSP00000419117,ENSP00000435777,ENSP00000443824,ENSP00000451261,ENSP00000464813,ENSP00000473631 | CDC20,DCP2,DNAJC3,EXOSC2,EXOSC5,EXOSC7,GZMA,PPP2R2A,PSMA3,PSMA4,PSMC6,RPL14,RPL15,RPL18A,RPL27,RPL27A,RPL34,RPL35A,RPL5,RPL6,RPL7,RPL8,RPL9,RPS11,RPS13,RPS14,RPS2,RPS8,UBE2G1,UBE2J1,UBE2K,ZNRF3 |
| GO:1901566 | organonitrogen compound biosynthetic process | 42 | 1370 | 1.67E-07 | ENSP00000210060,ENSP00000222247,ENSP00000223273,ENSP00000229239,ENSP00000233242,ENSP00000252602,ENSP00000253099,ENSP00000256578,ENSP00000259056,ENSP00000260600,ENSP00000262584,ENSP00000270625,ENSP00000287820,ENSP00000309334,ENSP00000312631,ENSP00000316335,ENSP00000339795,ENSP00000341885,ENSP00000344260,ENSP00000346015,ENSP00000353910,ENSP00000359345,ENSP00000363852,ENSP00000364145,ENSP00000364212,ENSP00000367038,ENSP00000368927,ENSP00000369519,ENSP00000377836,ENSP00000378163,ENSP00000379506,ENSP00000379888,ENSP00000385958,ENSP00000389176,ENSP00000400467,ENSP00000403172,ENSP00000419117,ENSP00000428340,ENSP00000435777,ENSP00000451261,ENSP00000464813,ENSP00000483552 | ADCY3,AMPD2,ANGPT1,APOB,C1GALT1,CDA,DHPS,EIF1AX,FARS2,FURIN,FUT8,GALNT15,GALNT5,GAPDH,GPC3,INSM1,MRPL34,MRPL4,MTAP,PFKFB1,PPARG,RPL14,RPL15,RPL18A,RPL27,RPL27A,RPL34,RPL35A,RPL5,RPL6,RPL7,RPL8,RPL9,RPS11,RPS13,RPS14,RPS2,RPS8,RRBP1,SLC44A1,UBE2J1,YAE1D1 |
| GO:0072657 | protein localization to membrane | 22 | 405 | 1.74E-07 | ENSP00000222247,ENSP00000262584,ENSP00000270625,ENSP00000309334,ENSP00000327716,ENSP00000339795,ENSP00000341885,ENSP00000346015,ENSP00000346151,ENSP00000356042,ENSP00000359345,ENSP00000365606,ENSP00000378163,ENSP00000379506,ENSP00000379888,ENSP00000385958,ENSP00000400467,ENSP00000403172,ENSP00000419117,ENSP00000435777,ENSP00000464813,ENSP00000479322 | CLIP1,EZR,GRIPAP1,MAGI2,RPL14,RPL15,RPL18A,RPL27,RPL27A,RPL34,RPL35A,RPL5,RPL6,RPL7,RPL8,RPL9,RPS11,RPS13,RPS14,RPS2,RPS8,WRB |
| GO:0010605 | negative regulation of macromolecule metabolic process | 61 | 2558 | 2.25E-07 | ENSP00000221233,ENSP00000222247,ENSP00000229239,ENSP00000240185,ENSP00000244769,ENSP00000262584,ENSP00000265564,ENSP00000270625,ENSP00000273317,ENSP00000274306,ENSP00000276431,ENSP00000280154,ENSP00000285670,ENSP00000286234,ENSP00000287820,ENSP00000294304,ENSP00000296140,ENSP00000296953,ENSP00000299084,ENSP00000303427,ENSP00000309334,ENSP00000312631,ENSP00000320176,ENSP00000323696,ENSP00000325074,ENSP00000327025,ENSP00000339795,ENSP00000341680,ENSP00000341885,ENSP00000345917,ENSP00000346015,ENSP00000349016,ENSP00000354518,ENSP00000356042,ENSP00000359345,ENSP00000361433,ENSP00000366843,ENSP00000373715,ENSP00000377836,ENSP00000378163,ENSP00000379506,ENSP00000379888,ENSP00000384371,ENSP00000385958,ENSP00000387122,ENSP00000395007,ENSP00000400467,ENSP00000403172,ENSP00000413929,ENSP00000419117,ENSP00000419692,ENSP00000420095,ENSP00000428340,ENSP00000430432,ENSP00000435777,ENSP00000451261,ENSP00000464813,ENSP00000469880,ENSP00000473631,ENSP00000475814,ENSP00000483552 | ANGPT1,ATXN1,ATXN2,BCL6,CCR1,CLEC16A,CREBRF,CRTAP,DCP2,DEPTOR,DNAJC3,DTNBP1,EXOSC2,EXOSC5,EXOSC7,EZR,FURIN,GAPDH,GPC3,GZMA,HCLS1,HDAC2,HIST2H2AA3,INSM1,LIMD1,LRP5,LYAR,PDCD4,PDS5A,PEX14,PPARG,PPP2R2A,RCOR3,RORC,RPL14,RPL15,RPL18A,RPL27,RPL27A,RPL34,RPL35A,RPL5,RPL6,RPL7,RPL8,RPL9,RPS11,RPS13,RPS14,RPS2,RPS8,RXRA,SAMSN1,SPRED1,TARDBP,TNFRSF10B,UBE2J1,VRK3,ZMYND8,ZNF746,ZNF830 |
| GO:0009892 | negative regulation of metabolic process | 64 | 2762 | 2.29E-07 | ENSP00000221233,ENSP00000222247,ENSP00000229239,ENSP00000240185,ENSP00000244769,ENSP00000262584,ENSP00000265564,ENSP00000270625,ENSP00000273317,ENSP00000274306,ENSP00000276431,ENSP00000280154,ENSP00000285670,ENSP00000286234,ENSP00000287820,ENSP00000294304,ENSP00000296140,ENSP00000296953,ENSP00000299084,ENSP00000303427,ENSP00000309334,ENSP00000312631,ENSP00000320176,ENSP00000323696,ENSP00000325074,ENSP00000327025,ENSP00000339795,ENSP00000341680,ENSP00000341885,ENSP00000345917,ENSP00000346015,ENSP00000347742,ENSP00000349016,ENSP00000354518,ENSP00000356042,ENSP00000359345,ENSP00000361433,ENSP00000364145,ENSP00000364212,ENSP00000366843,ENSP00000373715,ENSP00000377836,ENSP00000378163,ENSP00000379506,ENSP00000379888,ENSP00000384371,ENSP00000385958,ENSP00000387122,ENSP00000395007,ENSP00000400467,ENSP00000403172,ENSP00000413929,ENSP00000419117,ENSP00000419692,ENSP00000420095,ENSP00000428340,ENSP00000430432,ENSP00000435777,ENSP00000451261,ENSP00000464813,ENSP00000469880,ENSP00000473631,ENSP00000475814,ENSP00000483552 | ANGPT1,ATXN1,ATXN2,BCL6,CCR1,CDA,CLEC16A,CREBRF,CRTAP,DCP2,DEPTOR,DNAJC3,DTNBP1,EXOSC2,EXOSC5,EXOSC7,EZR,FURIN,GAPDH,GPC3,GZMA,HCLS1,HDAC2,HIST2H2AA3,INSM1,LIMD1,LRP5,LYAR,PDCD4,PDS5A,PDZD3,PEX14,PFKFB1,PPARG,PPP2R2A,RCOR3,RORC,RPL14,RPL15,RPL18A,RPL27,RPL27A,RPL34,RPL35A,RPL5,RPL6,RPL7,RPL8,RPL9,RPS11,RPS13,RPS14,RPS2,RPS8,RXRA,SAMSN1,SPRED1,TARDBP,TNFRSF10B,UBE2J1,VRK3,ZMYND8,ZNF746,ZNF830 |
| GO:0009057 | macromolecule catabolic process | 34 | 970 | 2.85E-07 | ENSP00000044462,ENSP00000216455,ENSP00000221233,ENSP00000222247,ENSP00000233242,ENSP00000261427,ENSP00000262584,ENSP00000265564,ENSP00000270625,ENSP00000274306,ENSP00000309334,ENSP00000325074,ENSP00000339795,ENSP00000341885,ENSP00000346015,ENSP00000359345,ENSP00000361433,ENSP00000361540,ENSP00000373715,ENSP00000377836,ENSP00000378163,ENSP00000379506,ENSP00000379888,ENSP00000380178,ENSP00000385958,ENSP00000400467,ENSP00000401802,ENSP00000403172,ENSP00000419117,ENSP00000435777,ENSP00000443824,ENSP00000451261,ENSP00000464813,ENSP00000473631 | APOB,CDC20,DCP2,DNAJC3,EXOSC2,EXOSC5,EXOSC7,GPC3,GZMA,PPP2R2A,PSMA3,PSMA4,PSMC6,RPL14,RPL15,RPL18A,RPL27,RPL27A,RPL34,RPL35A,RPL5,RPL6,RPL7,RPL8,RPL9,RPS11,RPS13,RPS14,RPS2,RPS8,UBE2G1,UBE2J1,UBE2K,ZNRF3 |
| GO:0009056 | catabolic process | 49 | 1859 | 6.00E-07 | ENSP00000044462,ENSP00000210060,ENSP00000215730,ENSP00000216455,ENSP00000221233,ENSP00000222247,ENSP00000229239,ENSP00000233242,ENSP00000258317,ENSP00000261427,ENSP00000262584,ENSP00000265564,ENSP00000270625,ENSP00000274306,ENSP00000294304,ENSP00000304642,ENSP00000309334,ENSP00000310226,ENSP00000325074,ENSP00000339795,ENSP00000341885,ENSP00000346015,ENSP00000353910,ENSP00000357722,ENSP00000359345,ENSP00000361433,ENSP00000361540,ENSP00000363852,ENSP00000364145,ENSP00000364212,ENSP00000373715,ENSP00000377836,ENSP00000378163,ENSP00000379506,ENSP00000379888,ENSP00000380178,ENSP00000385080,ENSP00000385958,ENSP00000387122,ENSP00000400467,ENSP00000401802,ENSP00000403172,ENSP00000419117,ENSP00000435777,ENSP00000443824,ENSP00000451261,ENSP00000464813,ENSP00000473631,ENSP00000483552 | APOB,CDA,CDC20,CLEC16A,DCP2,DHPS,DNAJC3,EXOSC2,EXOSC5,EXOSC7,FAH,FAM134B,FURIN,FUT8,GAPDH,GPC3,GZMA,LRP5,NPL,PFKFB1,PPP2R2A,PSMA3,PSMA4,PSMC6,RAB1B,RPL14,RPL15,RPL18A,RPL27,RPL27A,RPL34,RPL35A,RPL5,RPL6,RPL7,RPL8,RPL9,RPS11,RPS13,RPS14,RPS2,RPS8,S100A8,SLC44A1,SNAP29,UBE2G1,UBE2J1,UBE2K,ZNRF3 |
| GO:0042254 | ribosome biogenesis | 17 | 270 | 1.56E-06 | ENSP00000221233,ENSP00000223273,ENSP00000265564,ENSP00000339795,ENSP00000341885,ENSP00000345917,ENSP00000359345,ENSP00000359859,ENSP00000361433,ENSP00000378163,ENSP00000379506,ENSP00000379888,ENSP00000385958,ENSP00000403172,ENSP00000419117,ENSP00000423067,ENSP00000464813 | EXOSC2,EXOSC5,EXOSC7,LYAR,MTG2,RPL14,RPL27,RPL34,RPL35A,RPL5,RPL6,RPL7,RPS14,RPS2,RPS8,WDR36,YAE1D1 |
| GO:0006605 | protein targeting | 18 | 318 | 2.63E-06 | ENSP00000222247,ENSP00000262584,ENSP00000270625,ENSP00000309334,ENSP00000339795,ENSP00000341885,ENSP00000346015,ENSP00000349016,ENSP00000359345,ENSP00000378163,ENSP00000379506,ENSP00000379888,ENSP00000385958,ENSP00000400467,ENSP00000403172,ENSP00000419117,ENSP00000435777,ENSP00000464813 | PEX14,RPL14,RPL15,RPL18A,RPL27,RPL27A,RPL34,RPL35A,RPL5,RPL6,RPL7,RPL8,RPL9,RPS11,RPS13,RPS14,RPS2,RPS8 |
| GO:0044248 | cellular catabolic process | 44 | 1646 | 2.63E-06 | ENSP00000044462,ENSP00000215730,ENSP00000216455,ENSP00000221233,ENSP00000222247,ENSP00000229239,ENSP00000233242,ENSP00000258317,ENSP00000261427,ENSP00000262584,ENSP00000265564,ENSP00000270625,ENSP00000274306,ENSP00000304642,ENSP00000309334,ENSP00000310226,ENSP00000325074,ENSP00000339795,ENSP00000341885,ENSP00000346015,ENSP00000357722,ENSP00000359345,ENSP00000361433,ENSP00000361540,ENSP00000363852,ENSP00000364145,ENSP00000364212,ENSP00000373715,ENSP00000378163,ENSP00000379506,ENSP00000379888,ENSP00000380178,ENSP00000385080,ENSP00000385958,ENSP00000387122,ENSP00000400467,ENSP00000401802,ENSP00000403172,ENSP00000419117,ENSP00000435777,ENSP00000443824,ENSP00000451261,ENSP00000464813,ENSP00000473631 | APOB,CDA,CDC20,CLEC16A,DCP2,DNAJC3,EXOSC2,EXOSC5,EXOSC7,FAH,FAM134B,GAPDH,GZMA,NPL,PFKFB1,PPP2R2A,PSMA3,PSMA4,PSMC6,RAB1B,RPL14,RPL15,RPL18A,RPL27,RPL27A,RPL34,RPL35A,RPL5,RPL6,RPL7,RPL8,RPL9,RPS11,RPS13,RPS14,RPS2,RPS8,S100A8,SLC44A1,SNAP29,UBE2G1,UBE2J1,UBE2K,ZNRF3 |
| GO:0072594 | establishment of protein localization to organelle | 20 | 396 | 2.63E-06 | ENSP00000222247,ENSP00000262584,ENSP00000270625,ENSP00000309334,ENSP00000339795,ENSP00000341885,ENSP00000346015,ENSP00000349016,ENSP00000357552,ENSP00000359345,ENSP00000378163,ENSP00000379506,ENSP00000379888,ENSP00000384371,ENSP00000385958,ENSP00000400467,ENSP00000403172,ENSP00000419117,ENSP00000435777,ENSP00000464813 | BCL6,KPNA5,PEX14,RPL14,RPL15,RPL18A,RPL27,RPL27A,RPL34,RPL35A,RPL5,RPL6,RPL7,RPL8,RPL9,RPS11,RPS13,RPS14,RPS2,RPS8 |
| GO:0010629 | negative regulation of gene expression | 44 | 1670 | 3.81E-06 | ENSP00000221233,ENSP00000222247,ENSP00000229239,ENSP00000240185,ENSP00000244769,ENSP00000262584,ENSP00000265564,ENSP00000270625,ENSP00000273317,ENSP00000280154,ENSP00000287820,ENSP00000296140,ENSP00000296953,ENSP00000309334,ENSP00000312631,ENSP00000320176,ENSP00000325074,ENSP00000327025,ENSP00000339795,ENSP00000341885,ENSP00000345917,ENSP00000346015,ENSP00000349016,ENSP00000356042,ENSP00000359345,ENSP00000361433,ENSP00000373715,ENSP00000378163,ENSP00000379506,ENSP00000379888,ENSP00000384371,ENSP00000385958,ENSP00000395007,ENSP00000400467,ENSP00000403172,ENSP00000413929,ENSP00000419117,ENSP00000419692,ENSP00000420095,ENSP00000430432,ENSP00000435777,ENSP00000464813,ENSP00000475814,ENSP00000483552 | ATXN1,BCL6,CCR1,CREBRF,DCP2,EXOSC2,EXOSC5,EXOSC7,EZR,FURIN,GAPDH,HCLS1,HDAC2,HIST2H2AA3,INSM1,LIMD1,LYAR,PDCD4,PEX14,PPARG,PPP2R2A,RCOR3,RORC,RPL14,RPL15,RPL18A,RPL27,RPL27A,RPL34,RPL35A,RPL5,RPL6,RPL7,RPL8,RPL9,RPS11,RPS13,RPS14,RPS2,RPS8,RXRA,TARDBP,ZMYND8,ZNF746 |
| GO:1901575 | organic substance catabolic process | 43 | 1609 | 3.81E-06 | ENSP00000044462,ENSP00000210060,ENSP00000216455,ENSP00000221233,ENSP00000222247,ENSP00000229239,ENSP00000233242,ENSP00000258317,ENSP00000261427,ENSP00000262584,ENSP00000265564,ENSP00000270625,ENSP00000274306,ENSP00000294304,ENSP00000309334,ENSP00000325074,ENSP00000339795,ENSP00000341885,ENSP00000346015,ENSP00000353910,ENSP00000359345,ENSP00000361433,ENSP00000361540,ENSP00000363852,ENSP00000364145,ENSP00000364212,ENSP00000373715,ENSP00000377836,ENSP00000378163,ENSP00000379506,ENSP00000379888,ENSP00000380178,ENSP00000385080,ENSP00000385958,ENSP00000400467,ENSP00000401802,ENSP00000403172,ENSP00000419117,ENSP00000435777,ENSP00000443824,ENSP00000451261,ENSP00000464813,ENSP00000473631 | APOB,CDA,CDC20,DCP2,DHPS,DNAJC3,EXOSC2,EXOSC5,EXOSC7,FAH,FUT8,GAPDH,GPC3,GZMA,LRP5,NPL,PFKFB1,PPP2R2A,PSMA3,PSMA4,PSMC6,RPL14,RPL15,RPL18A,RPL27,RPL27A,RPL34,RPL35A,RPL5,RPL6,RPL7,RPL8,RPL9,RPS11,RPS13,RPS14,RPS2,RPS8,SLC44A1,UBE2G1,UBE2J1,UBE2K,ZNRF3 |
| GO:0006364 | rRNA processing | 13 | 192 | 3.09E-05 | ENSP00000221233,ENSP00000265564,ENSP00000339795,ENSP00000341885,ENSP00000345917,ENSP00000359345,ENSP00000361433,ENSP00000379506,ENSP00000379888,ENSP00000385958,ENSP00000419117,ENSP00000423067,ENSP00000464813 | EXOSC2,EXOSC5,EXOSC7,LYAR,RPL14,RPL27,RPL35A,RPL5,RPL7,RPS14,RPS2,RPS8,WDR36 |
| GO:0002181 | cytoplasmic translation | 8 | 57 | 3.98E-05 | ENSP00000222247,ENSP00000262584,ENSP00000309334,ENSP00000339795,ENSP00000346015,ENSP00000400467,ENSP00000403172,ENSP00000419117 | RPL15,RPL18A,RPL27A,RPL35A,RPL6,RPL7,RPL8,RPL9 |
| GO:0048519 | negative regulation of biological process | 87 | 4953 | 3.99E-05 | ENSP00000221233,ENSP00000222247,ENSP00000229239,ENSP00000236273,ENSP00000240185,ENSP00000244769,ENSP00000250003,ENSP00000262584,ENSP00000265074,ENSP00000265564,ENSP00000270625,ENSP00000273317,ENSP00000274306,ENSP00000276431,ENSP00000280154,ENSP00000285670,ENSP00000286234,ENSP00000287820,ENSP00000294304,ENSP00000296140,ENSP00000296953,ENSP00000299084,ENSP00000301455,ENSP00000303427,ENSP00000304642,ENSP00000306822,ENSP00000309334,ENSP00000310557,ENSP00000312631,ENSP00000320176,ENSP00000323074,ENSP00000323696,ENSP00000325074,ENSP00000327025,ENSP00000330572,ENSP00000337946,ENSP00000339795,ENSP00000341680,ENSP00000341885,ENSP00000344242,ENSP00000345917,ENSP00000346015,ENSP00000346151,ENSP00000347742,ENSP00000349016,ENSP00000350348,ENSP00000354518,ENSP00000356042,ENSP00000358795,ENSP00000359345,ENSP00000361433,ENSP00000361824,ENSP00000364145,ENSP00000364212,ENSP00000366843,ENSP00000373715,ENSP00000377836,ENSP00000378163,ENSP00000378974,ENSP00000379506,ENSP00000379888,ENSP00000384371,ENSP00000385958,ENSP00000386766,ENSP00000387122,ENSP00000392660,ENSP00000395007,ENSP00000400467,ENSP00000403172,ENSP00000413929,ENSP00000419117,ENSP00000419692,ENSP00000420095,ENSP00000420211,ENSP00000428340,ENSP00000429900,ENSP00000430432,ENSP00000435777,ENSP00000440864,ENSP00000443824,ENSP00000451261,ENSP00000464813,ENSP00000469880,ENSP00000473631,ENSP00000475814,ENSP00000483552,ENSP00000484288 | ANGPT1,ANGPTL4,ARHGAP28,ASAP1,ATXN1,ATXN2,AVEN,BCL6,CCR1,CDA,CLEC16A,CREBRF,CRTAP,DCP2,DEPTOR,DNAJC3,DTNBP1,EXOSC2,EXOSC5,EXOSC7,EZR,FAM134B,FURIN,GAPDH,GPC3,GRM7,GZMA,HCLS1,HDAC2,HIST2H2AA3,INSM1,KCNE3,LIG4,LIMD1,LRP5,LRRC17,LYAR,MAGI2,MAPK8,MAPK8IP2,MYOD1,NEURL1,NPR3,NR4A1,PACRG,PDCD4,PDS5A,PDZD3,PEX14,PFKFB1,PPARG,PPP2R2A,RAI1,RCOR3,RGL2,RORC,RPL14,RPL15,RPL18A,RPL27,RPL27A,RPL34,RPL35A,RPL5,RPL6,RPL7,RPL8,RPL9,RPS11,RPS13,RPS14,RPS2,RPS8,RXRA,SAMSN1,SLC39A10,SPRED1,SPTAN1,SYF2,TARDBP,TNFRSF10B,UBE2J1,VRK3,ZMYND8,ZNF746,ZNF830,ZNRF3 |
| GO:0006396 | RNA processing | 26 | 825 | 0.00013 | ENSP00000221233,ENSP00000230859,ENSP00000236273,ENSP00000240185,ENSP00000244769,ENSP00000265564,ENSP00000293860,ENSP00000300291,ENSP00000316335,ENSP00000339795,ENSP00000341885,ENSP00000345917,ENSP00000354518,ENSP00000359345,ENSP00000361433,ENSP00000379506,ENSP00000379888,ENSP00000384792,ENSP00000385958,ENSP00000394085,ENSP00000418563,ENSP00000419117,ENSP00000423067,ENSP00000443926,ENSP00000452123,ENSP00000464813 | ATXN1,CELF2,EXOSC2,EXOSC5,EXOSC7,FARS2,JMJD6,LYAR,NUDT21,PAPD7,POLR3K,RPL14,RPL27,RPL35A,RPL5,RPL7,RPS14,RPS2,RPS8,SRSF5,SYF2,TARDBP,U2SURP,WDR36,WDR62,ZNF830 |
| GO:0060255 | regulation of macromolecule metabolic process | 97 | 6072 | 0.00044 | ENSP00000211936,ENSP00000216455,ENSP00000221233,ENSP00000222247,ENSP00000229239,ENSP00000233242,ENSP00000237853,ENSP00000240185,ENSP00000244769,ENSP00000250003,ENSP00000261427,ENSP00000262319,ENSP00000262584,ENSP00000265564,ENSP00000265981,ENSP00000270625,ENSP00000273317,ENSP00000274026,ENSP00000274306,ENSP00000276431,ENSP00000280154,ENSP00000285670,ENSP00000286234,ENSP00000287820,ENSP00000291547,ENSP00000294304,ENSP00000296140,ENSP00000296953,ENSP00000299084,ENSP00000300291,ENSP00000303427,ENSP00000309334,ENSP00000310226,ENSP00000312631,ENSP00000320176,ENSP00000323074,ENSP00000323696,ENSP00000324463,ENSP00000325074,ENSP00000327025,ENSP00000330572,ENSP00000338788,ENSP00000339795,ENSP00000341680,ENSP00000341885,ENSP00000345917,ENSP00000346015,ENSP00000346151,ENSP00000349016,ENSP00000353910,ENSP00000354518,ENSP00000356042,ENSP00000356331,ENSP00000356399,ENSP00000357722,ENSP00000358795,ENSP00000359345,ENSP00000359859,ENSP00000361014,ENSP00000361433,ENSP00000361540,ENSP00000366843,ENSP00000373715,ENSP00000376534,ENSP00000377836,ENSP00000378163,ENSP00000378974,ENSP00000379506,ENSP00000379888,ENSP00000384000,ENSP00000384371,ENSP00000385958,ENSP00000386184,ENSP00000386766,ENSP00000387122,ENSP00000393795,ENSP00000394085,ENSP00000395007,ENSP00000400467,ENSP00000401802,ENSP00000403172,ENSP00000413929,ENSP00000418287,ENSP00000419117,ENSP00000419692,ENSP00000420095,ENSP00000428340,ENSP00000430432,ENSP00000435777,ENSP00000440864,ENSP00000451261,ENSP00000452123,ENSP00000464813,ENSP00000469880,ENSP00000473631,ENSP00000475814,ENSP00000483552 | ANGPT1,APOB,ATXN1,ATXN2,BAZ2B,BCL6,CCNA2,CCR1,CDC20,CFH,CIAO1,CLEC16A,CREBRF,CRTAP,DCP2,DEPTOR,DNAJC3,DTNBP1,ELL2,EXOSC2,EXOSC5,EXOSC7,EZR,FURIN,FUT8,GAPDH,GPC3,GZMA,HCLS1,HDAC2,HIST2H2AA3,INSM1,JMJD6,LIMD1,LRP5,LYAR,MAGI2,MAPK8,MAPK8IP2,MKNK1,MTG2,MYOD1,NEURL1,NR4A1,NR5A2,NUDT21,PDCD4,PDS5A,PEX14,PKNOX1,PPARG,PPP2R2A,PSMA3,PSMC6,RAB1B,RAI1,RCOR3,RNF141,RORC,RPL14,RPL15,RPL18A,RPL27,RPL27A,RPL34,RPL35A,RPL5,RPL6,RPL7,RPL8,RPL9,RPS11,RPS13,RPS14,RPS2,RPS8,RXRA,S100A8,SAMSN1,SLC39A10,SPRED1,SRSF5,TARDBP,TELO2,TNFRSF10B,UBE2J1,UBE2K,VRK3,YLPM1,ZBED8,ZC3H15,ZMYND8,ZNF184,ZNF512B,ZNF681,ZNF746,ZNF830 |
| GO:0019222 | regulation of metabolic process | 102 | 6516 | 0.0005 | ENSP00000211936,ENSP00000216455,ENSP00000221233,ENSP00000222247,ENSP00000229239,ENSP00000233242,ENSP00000237853,ENSP00000240185,ENSP00000244769,ENSP00000250003,ENSP00000261427,ENSP00000262319,ENSP00000262584,ENSP00000265074,ENSP00000265564,ENSP00000265981,ENSP00000270625,ENSP00000273317,ENSP00000274026,ENSP00000274306,ENSP00000276431,ENSP00000280154,ENSP00000285670,ENSP00000286234,ENSP00000287820,ENSP00000291547,ENSP00000294304,ENSP00000296140,ENSP00000296953,ENSP00000299084,ENSP00000300291,ENSP00000301455,ENSP00000303427,ENSP00000309334,ENSP00000310226,ENSP00000312631,ENSP00000320176,ENSP00000323074,ENSP00000323696,ENSP00000324463,ENSP00000325074,ENSP00000327025,ENSP00000330572,ENSP00000338788,ENSP00000339795,ENSP00000341680,ENSP00000341885,ENSP00000345917,ENSP00000346015,ENSP00000346151,ENSP00000347742,ENSP00000349016,ENSP00000353910,ENSP00000354518,ENSP00000356042,ENSP00000356331,ENSP00000356399,ENSP00000357722,ENSP00000358795,ENSP00000359345,ENSP00000359859,ENSP00000361014,ENSP00000361433,ENSP00000361540,ENSP00000364145,ENSP00000364212,ENSP00000366843,ENSP00000373715,ENSP00000376534,ENSP00000377836,ENSP00000378163,ENSP00000378974,ENSP00000379506,ENSP00000379888,ENSP00000384000,ENSP00000384371,ENSP00000385958,ENSP00000386184,ENSP00000386766,ENSP00000387122,ENSP00000393795,ENSP00000394085,ENSP00000395007,ENSP00000400467,ENSP00000401802,ENSP00000403172,ENSP00000413929,ENSP00000418287,ENSP00000419117,ENSP00000419692,ENSP00000420095,ENSP00000428340,ENSP00000430432,ENSP00000435777,ENSP00000440864,ENSP00000451261,ENSP00000452123,ENSP00000464813,ENSP00000469880,ENSP00000473631,ENSP00000475814,ENSP00000483552 | ANGPT1,ANGPTL4,APOB,ATXN1,ATXN2,BAZ2B,BCL6,CCNA2,CCR1,CDA,CDC20,CFH,CIAO1,CLEC16A,CREBRF,CRTAP,DCP2,DEPTOR,DNAJC3,DTNBP1,ELL2,EXOSC2,EXOSC5,EXOSC7,EZR,FURIN,FUT8,GAPDH,GPC3,GZMA,HCLS1,HDAC2,HIST2H2AA3,INSM1,JMJD6,LIMD1,LRP5,LYAR,MAGI2,MAPK8,MAPK8IP2,MKNK1,MTG2,MYOD1,NEURL1,NPR3,NR4A1,NR5A2,NUDT21,PDCD4,PDS5A,PDZD3,PEX14,PFKFB1,PKNOX1,PPARG,PPP2R2A,PSMA3,PSMC6,RAB1B,RAI1,RCOR3,RNF141,RORC,RPL14,RPL15,RPL18A,RPL27,RPL27A,RPL34,RPL35A,RPL5,RPL6,RPL7,RPL8,RPL9,RPS11,RPS13,RPS14,RPS2,RPS8,RXRA,S100A8,SAMSN1,SLC39A10,SPRED1,SRSF5,TARDBP,TELO2,TNFRSF10B,UBE2J1,UBE2K,VRK3,YLPM1,ZBED8,ZC3H15,ZMYND8,ZNF184,ZNF512B,ZNF681,ZNF746,ZNF830 |
| GO:0034470 | ncRNA processing | 15 | 340 | 0.00054 | ENSP00000221233,ENSP00000265564,ENSP00000293860,ENSP00000316335,ENSP00000339795,ENSP00000341885,ENSP00000345917,ENSP00000359345,ENSP00000361433,ENSP00000379506,ENSP00000379888,ENSP00000385958,ENSP00000419117,ENSP00000423067,ENSP00000464813 | EXOSC2,EXOSC5,EXOSC7,FARS2,LYAR,POLR3K,RPL14,RPL27,RPL35A,RPL5,RPL7,RPS14,RPS2,RPS8,WDR36 |
| GO:0033365 | protein localization to organelle | 21 | 649 | 0.00085 | ENSP00000222247,ENSP00000262584,ENSP00000270625,ENSP00000309334,ENSP00000327716,ENSP00000339795,ENSP00000341885,ENSP00000346015,ENSP00000349016,ENSP00000357552,ENSP00000359345,ENSP00000378163,ENSP00000379506,ENSP00000379888,ENSP00000384371,ENSP00000385958,ENSP00000400467,ENSP00000403172,ENSP00000419117,ENSP00000435777,ENSP00000464813 | BCL6,KPNA5,PEX14,RPL14,RPL15,RPL18A,RPL27,RPL27A,RPL34,RPL35A,RPL5,RPL6,RPL7,RPL8,RPL9,RPS11,RPS13,RPS14,RPS2,RPS8,WRB |
| GO:0010468 | regulation of gene expression | 77 | 4533 | 0.00089 | ENSP00000211936,ENSP00000221233,ENSP00000222247,ENSP00000229239,ENSP00000233242,ENSP00000237853,ENSP00000240185,ENSP00000244769,ENSP00000250003,ENSP00000262584,ENSP00000265564,ENSP00000265981,ENSP00000270625,ENSP00000273317,ENSP00000274026,ENSP00000280154,ENSP00000287820,ENSP00000291547,ENSP00000294304,ENSP00000296140,ENSP00000296953,ENSP00000300291,ENSP00000309334,ENSP00000312631,ENSP00000320176,ENSP00000323074,ENSP00000324463,ENSP00000325074,ENSP00000327025,ENSP00000338788,ENSP00000339795,ENSP00000341680,ENSP00000341885,ENSP00000345917,ENSP00000346015,ENSP00000349016,ENSP00000353910,ENSP00000356042,ENSP00000356331,ENSP00000356399,ENSP00000357722,ENSP00000358795,ENSP00000359345,ENSP00000359859,ENSP00000361014,ENSP00000361433,ENSP00000366843,ENSP00000373715,ENSP00000376534,ENSP00000377836,ENSP00000378163,ENSP00000378974,ENSP00000379506,ENSP00000379888,ENSP00000384000,ENSP00000384371,ENSP00000385958,ENSP00000386184,ENSP00000393795,ENSP00000394085,ENSP00000395007,ENSP00000400467,ENSP00000401802,ENSP00000403172,ENSP00000413929,ENSP00000418287,ENSP00000419117,ENSP00000419692,ENSP00000420095,ENSP00000430432,ENSP00000435777,ENSP00000440864,ENSP00000452123,ENSP00000464813,ENSP00000473631,ENSP00000475814,ENSP00000483552 | APOB,ATXN1,ATXN2,BAZ2B,BCL6,CCNA2,CCR1,CFH,CIAO1,CREBRF,DCP2,DNAJC3,DTNBP1,ELL2,EXOSC2,EXOSC5,EXOSC7,EZR,FURIN,FUT8,GAPDH,GPC3,HCLS1,HDAC2,HIST2H2AA3,INSM1,JMJD6,LIMD1,LRP5,LYAR,MAPK8,MKNK1,MTG2,MYOD1,NEURL1,NR4A1,NR5A2,NUDT21,PDCD4,PEX14,PKNOX1,PPARG,PPP2R2A,PSMC6,RAI1,RCOR3,RNF141,RORC,RPL14,RPL15,RPL18A,RPL27,RPL27A,RPL34,RPL35A,RPL5,RPL6,RPL7,RPL8,RPL9,RPS11,RPS13,RPS14,RPS2,RPS8,RXRA,S100A8,SRSF5,TARDBP,YLPM1,ZBED8,ZC3H15,ZMYND8,ZNF184,ZNF512B,ZNF681,ZNF746 |
| GO:0044267 | cellular protein metabolic process | 64 | 3603 | 0.0018 | ENSP00000044462,ENSP00000210060,ENSP00000216455,ENSP00000222247,ENSP00000223273,ENSP00000229239,ENSP00000233242,ENSP00000238616,ENSP00000250003,ENSP00000252602,ENSP00000253099,ENSP00000259056,ENSP00000261427,ENSP00000262584,ENSP00000265981,ENSP00000270625,ENSP00000274026,ENSP00000274306,ENSP00000299084,ENSP00000305976,ENSP00000306461,ENSP00000309334,ENSP00000310226,ENSP00000316335,ENSP00000323696,ENSP00000325074,ENSP00000330572,ENSP00000339795,ENSP00000341885,ENSP00000344260,ENSP00000346015,ENSP00000349016,ENSP00000353910,ENSP00000357722,ENSP00000358795,ENSP00000359345,ENSP00000361014,ENSP00000361540,ENSP00000361824,ENSP00000367038,ENSP00000368927,ENSP00000377836,ENSP00000378163,ENSP00000378974,ENSP00000379506,ENSP00000379888,ENSP00000380178,ENSP00000385958,ENSP00000389176,ENSP00000394085,ENSP00000400467,ENSP00000401802,ENSP00000403172,ENSP00000419117,ENSP00000420095,ENSP00000428340,ENSP00000430432,ENSP00000435777,ENSP00000443824,ENSP00000451261,ENSP00000464813,ENSP00000469880,ENSP00000473631,ENSP00000483552 | ANGPT1,APOB,C1GALT1,CCNA2,CDC20,CRTAP,DHPS,DNAJC3,EIF1AX,FARS2,FURIN,FUT8,GALNT15,GALNT5,GAPDH,GPC3,GZMA,HDAC2,JMJD6,MAPK8,MAPK8IP2,MKNK1,MRPL34,MRPL4,MYOD1,NEK9,NEURL1,PEX14,PPP2R2A,PSMA3,PSMA4,PSMC6,RAB1B,RNF141,RPL14,RPL15,RPL18A,RPL27,RPL27A,RPL34,RPL35A,RPL5,RPL6,RPL7,RPL8,RPL9,RPS11,RPS13,RPS14,RPS2,RPS8,RRBP1,S100A8,SIK2,SPRED1,SPRR1B,SPTAN1,UBE2G1,UBE2J1,UBE2K,VRK3,YAE1D1,ZMYND8,ZNRF3 |
| GO:0010608 | posttranscriptional regulation of gene expression | 16 | 441 | 0.0025 | ENSP00000221233,ENSP00000229239,ENSP00000240185,ENSP00000265564,ENSP00000273317,ENSP00000300291,ENSP00000358795,ENSP00000359345,ENSP00000359859,ENSP00000361014,ENSP00000361433,ENSP00000366843,ENSP00000373715,ENSP00000385958,ENSP00000419692,ENSP00000473631 | ATXN2,DCP2,DNAJC3,EXOSC2,EXOSC5,EXOSC7,GAPDH,LIMD1,MKNK1,MTG2,NEURL1,NUDT21,RPL5,RPS14,RXRA,TARDBP |
| GO:0006886 | intracellular protein transport | 23 | 836 | 0.0036 | ENSP00000222247,ENSP00000262584,ENSP00000270625,ENSP00000309334,ENSP00000339795,ENSP00000341885,ENSP00000346015,ENSP00000349016,ENSP00000357552,ENSP00000359345,ENSP00000365606,ENSP00000378163,ENSP00000379506,ENSP00000379888,ENSP00000384371,ENSP00000385958,ENSP00000400467,ENSP00000403172,ENSP00000419117,ENSP00000435777,ENSP00000452123,ENSP00000464813,ENSP00000479322 | BCL6,CLIP1,GRIPAP1,KPNA5,PEX14,RPL14,RPL15,RPL18A,RPL27,RPL27A,RPL34,RPL35A,RPL5,RPL6,RPL7,RPL8,RPL9,RPS11,RPS13,RPS14,RPS2,RPS8,SRSF5 |
| GO:0010467 | gene expression | 64 | 3733 | 0.005 | ENSP00000210060,ENSP00000211936,ENSP00000221233,ENSP00000222247,ENSP00000223273,ENSP00000225719,ENSP00000230859,ENSP00000236273,ENSP00000237853,ENSP00000240185,ENSP00000244769,ENSP00000250003,ENSP00000252602,ENSP00000253099,ENSP00000262584,ENSP00000265564,ENSP00000270625,ENSP00000273317,ENSP00000287820,ENSP00000291547,ENSP00000293860,ENSP00000296953,ENSP00000300291,ENSP00000309334,ENSP00000312631,ENSP00000316335,ENSP00000324463,ENSP00000327025,ENSP00000339795,ENSP00000341885,ENSP00000345917,ENSP00000346015,ENSP00000354518,ENSP00000356331,ENSP00000359345,ENSP00000361433,ENSP00000367038,ENSP00000368927,ENSP00000376534,ENSP00000378163,ENSP00000379506,ENSP00000379888,ENSP00000384000,ENSP00000384371,ENSP00000384792,ENSP00000385958,ENSP00000393795,ENSP00000394085,ENSP00000395007,ENSP00000400467,ENSP00000403172,ENSP00000413929,ENSP00000418563,ENSP00000419117,ENSP00000419692,ENSP00000420095,ENSP00000423067,ENSP00000430432,ENSP00000435777,ENSP00000440864,ENSP00000443926,ENSP00000452123,ENSP00000464813,ENSP00000483552 | ATXN1,BAZ2B,BCL6,CELF2,CPD,CREBRF,DHPS,EIF1AX,ELL2,EXOSC2,EXOSC5,EXOSC7,FARS2,FURIN,HDAC2,INSM1,JMJD6,LIMD1,LYAR,MRPL34,MRPL4,MYOD1,NR4A1,NR5A2,NUDT21,PAPD7,PKNOX1,POLR3K,PPARG,RCOR3,RORC,RPL14,RPL15,RPL18A,RPL27,RPL27A,RPL34,RPL35A,RPL5,RPL6,RPL7,RPL8,RPL9,RPS11,RPS13,RPS14,RPS2,RPS8,RRBP1,RXRA,SRSF5,SYF2,TARDBP,U2SURP,WDR36,WDR62,YAE1D1,YLPM1,ZMYND8,ZNF184,ZNF512B,ZNF681,ZNF746,ZNF830 |
| GO:0051234 | establishment of localization | 70 | 4248 | 0.0068 | ENSP00000215730,ENSP00000216487,ENSP00000222247,ENSP00000233242,ENSP00000236192,ENSP00000244769,ENSP00000246554,ENSP00000262584,ENSP00000265074,ENSP00000265715,ENSP00000270625,ENSP00000287490,ENSP00000287820,ENSP00000294304,ENSP00000296140,ENSP00000306185,ENSP00000309334,ENSP00000310226,ENSP00000310557,ENSP00000327716,ENSP00000339795,ENSP00000341680,ENSP00000341885,ENSP00000346015,ENSP00000346931,ENSP00000347742,ENSP00000348965,ENSP00000349016,ENSP00000349456,ENSP00000356042,ENSP00000357552,ENSP00000357722,ENSP00000358795,ENSP00000359345,ENSP00000359621,ENSP00000361824,ENSP00000362146,ENSP00000363298,ENSP00000363520,ENSP00000363852,ENSP00000364212,ENSP00000365477,ENSP00000365606,ENSP00000366843,ENSP00000367038,ENSP00000369860,ENSP00000371018,ENSP00000373734,ENSP00000378163,ENSP00000378974,ENSP00000379506,ENSP00000379888,ENSP00000384371,ENSP00000385028,ENSP00000385958,ENSP00000386766,ENSP00000394085,ENSP00000400467,ENSP00000403172,ENSP00000409544,ENSP00000411528,ENSP00000419117,ENSP00000419692,ENSP00000435777,ENSP00000444149,ENSP00000452123,ENSP00000464813,ENSP00000473631,ENSP00000479322,ENSP00000483552 | ANTXR2,APOB,ATXN1,ATXN2,BCL6,BSPRY,C6orf120,CCDC93,CCR1,CDA,CDCA8,CLCC1,CLDN4,CLIP1,COX6A2,COX6B1,DENND5B,DNAJC3,DTNBP1,DYNC1H1,EZR,FURIN,GJC1,GRIPAP1,JMJD6,KCNE3,KPNA5,LRP5,MAPK8,MCOLN3,MOSPD2,NEURL1,NIPAL3,NPR3,PDZD3,PEX14,PPARG,RAB1B,RIN3,RPL14,RPL15,RPL18A,RPL27,RPL27A,RPL34,RPL35A,RPL5,RPL6,RPL7,RPL8,RPL9,RPS11,RPS13,RPS14,RPS2,RPS8,RRBP1,RXRA,S100A8,SIRPB1,SLC22A23,SLC26A4,SLC39A10,SLC44A1,SLCO1C1,SNAP29,SPTAN1,SRSF5,VAMP4,WRB |
| GO:0044260 | cellular macromolecule metabolic process | 96 | 6413 | 0.007 | ENSP00000044462,ENSP00000210060,ENSP00000211936,ENSP00000216455,ENSP00000221233,ENSP00000222247,ENSP00000223273,ENSP00000229239,ENSP00000230859,ENSP00000233242,ENSP00000237853,ENSP00000238616,ENSP00000240185,ENSP00000244769,ENSP00000250003,ENSP00000252602,ENSP00000253099,ENSP00000259056,ENSP00000261427,ENSP00000262584,ENSP00000265564,ENSP00000265981,ENSP00000270625,ENSP00000273317,ENSP00000274026,ENSP00000274306,ENSP00000287820,ENSP00000291547,ENSP00000293860,ENSP00000296953,ENSP00000299084,ENSP00000300291,ENSP00000303427,ENSP00000305976,ENSP00000306461,ENSP00000309334,ENSP00000310226,ENSP00000312631,ENSP00000316335,ENSP00000323696,ENSP00000324463,ENSP00000325074,ENSP00000327025,ENSP00000330572,ENSP00000339795,ENSP00000341885,ENSP00000344260,ENSP00000345917,ENSP00000346015,ENSP00000349016,ENSP00000353910,ENSP00000354518,ENSP00000356042,ENSP00000356331,ENSP00000357722,ENSP00000358795,ENSP00000359345,ENSP00000361014,ENSP00000361433,ENSP00000361540,ENSP00000361824,ENSP00000367038,ENSP00000368927,ENSP00000373715,ENSP00000376534,ENSP00000377836,ENSP00000378163,ENSP00000378974,ENSP00000379506,ENSP00000379888,ENSP00000380178,ENSP00000384000,ENSP00000384371,ENSP00000385958,ENSP00000389176,ENSP00000393795,ENSP00000394085,ENSP00000395007,ENSP00000400467,ENSP00000401802,ENSP00000403172,ENSP00000413929,ENSP00000419117,ENSP00000419692,ENSP00000420095,ENSP00000428340,ENSP00000430432,ENSP00000435777,ENSP00000440864,ENSP00000443824,ENSP00000451261,ENSP00000464813,ENSP00000469880,ENSP00000473631,ENSP00000483552,ENSP00000484288 | ANGPT1,APOB,ATXN1,BAZ2B,BCL6,C1GALT1,CCNA2,CDC20,CREBRF,CRTAP,DCP2,DHPS,DNAJC3,EIF1AX,ELL2,EXOSC2,EXOSC5,EXOSC7,EZR,FARS2,FURIN,FUT8,GALNT15,GALNT5,GAPDH,GPC3,GZMA,HDAC2,INSM1,JMJD6,LIG4,LIMD1,LYAR,MAPK8,MAPK8IP2,MKNK1,MRPL34,MRPL4,MYOD1,NEK9,NEURL1,NR4A1,NR5A2,NUDT21,PAPD7,PDS5A,PEX14,PKNOX1,POLR3K,PPARG,PPP2R2A,PSMA3,PSMA4,PSMC6,RAB1B,RCOR3,RNF141,RORC,RPL14,RPL15,RPL18A,RPL27,RPL27A,RPL34,RPL35A,RPL5,RPL6,RPL7,RPL8,RPL9,RPS11,RPS13,RPS14,RPS2,RPS8,RRBP1,RXRA,S100A8,SIK2,SPRED1,SPRR1B,SPTAN1,TARDBP,UBE2G1,UBE2J1,UBE2K,VRK3,YAE1D1,YLPM1,ZMYND8,ZNF184,ZNF512B,ZNF681,ZNF746,ZNF830,ZNRF3 |
| GO:0019538 | protein metabolic process | 69 | 4194 | 0.008 | ENSP00000044462,ENSP00000210060,ENSP00000216455,ENSP00000222247,ENSP00000223273,ENSP00000225719,ENSP00000229239,ENSP00000233242,ENSP00000238616,ENSP00000250003,ENSP00000252602,ENSP00000253099,ENSP00000259056,ENSP00000261427,ENSP00000262584,ENSP00000265981,ENSP00000270625,ENSP00000274026,ENSP00000274306,ENSP00000287820,ENSP00000299084,ENSP00000300289,ENSP00000305976,ENSP00000306461,ENSP00000309334,ENSP00000310226,ENSP00000316335,ENSP00000323696,ENSP00000325074,ENSP00000330572,ENSP00000339795,ENSP00000341885,ENSP00000344260,ENSP00000346015,ENSP00000348308,ENSP00000349016,ENSP00000353910,ENSP00000356399,ENSP00000357722,ENSP00000358795,ENSP00000359345,ENSP00000361014,ENSP00000361540,ENSP00000361824,ENSP00000367038,ENSP00000368927,ENSP00000377836,ENSP00000378163,ENSP00000378974,ENSP00000379506,ENSP00000379888,ENSP00000380178,ENSP00000385958,ENSP00000389176,ENSP00000394085,ENSP00000400467,ENSP00000401802,ENSP00000403172,ENSP00000419117,ENSP00000420095,ENSP00000428340,ENSP00000430432,ENSP00000435777,ENSP00000443824,ENSP00000451261,ENSP00000464813,ENSP00000469880,ENSP00000473631,ENSP00000483552 | ANGPT1,APOB,C1GALT1,CCNA2,CDC20,CFH,CPD,CRTAP,DHPS,DNAJC3,EIF1AX,FARS2,FURIN,FUT8,GALNT15,GALNT5,GAPDH,GPC3,GZMA,HDAC2,JMJD6,MAPK8,MAPK8IP2,MKNK1,MMP23B,MRPL34,MRPL4,MYOD1,NEK9,NEURL1,PDIA3,PEX14,PPARG,PPP2R2A,PSMA3,PSMA4,PSMC6,RAB1B,RNF141,RPL14,RPL15,RPL18A,RPL27,RPL27A,RPL34,RPL35A,RPL5,RPL6,RPL7,RPL8,RPL9,RPS11,RPS13,RPS14,RPS2,RPS8,RRBP1,S100A8,SIK2,SPRED1,SPRR1B,SPTAN1,UBE2G1,UBE2J1,UBE2K,VRK3,YAE1D1,ZMYND8,ZNRF3 |
| GO:0006810 | transport | 68 | 4130 | 0.0086 | ENSP00000215730,ENSP00000216487,ENSP00000222247,ENSP00000233242,ENSP00000236192,ENSP00000244769,ENSP00000246554,ENSP00000262584,ENSP00000265074,ENSP00000265715,ENSP00000270625,ENSP00000287490,ENSP00000287820,ENSP00000294304,ENSP00000296140,ENSP00000306185,ENSP00000309334,ENSP00000310226,ENSP00000310557,ENSP00000339795,ENSP00000341680,ENSP00000341885,ENSP00000346015,ENSP00000346931,ENSP00000347742,ENSP00000348965,ENSP00000349016,ENSP00000349456,ENSP00000356042,ENSP00000357552,ENSP00000357722,ENSP00000358795,ENSP00000359345,ENSP00000359621,ENSP00000361824,ENSP00000363298,ENSP00000363520,ENSP00000363852,ENSP00000364212,ENSP00000365477,ENSP00000365606,ENSP00000366843,ENSP00000367038,ENSP00000369860,ENSP00000371018,ENSP00000373734,ENSP00000378163,ENSP00000378974,ENSP00000379506,ENSP00000379888,ENSP00000384371,ENSP00000385028,ENSP00000385958,ENSP00000386766,ENSP00000394085,ENSP00000400467,ENSP00000403172,ENSP00000409544,ENSP00000411528,ENSP00000419117,ENSP00000419692,ENSP00000435777,ENSP00000444149,ENSP00000452123,ENSP00000464813,ENSP00000473631,ENSP00000479322,ENSP00000483552 | ANTXR2,APOB,ATXN1,ATXN2,BCL6,BSPRY,C6orf120,CCDC93,CCR1,CDA,CLCC1,CLDN4,CLIP1,COX6A2,COX6B1,DENND5B,DNAJC3,DTNBP1,DYNC1H1,EZR,FURIN,GJC1,GRIPAP1,JMJD6,KCNE3,KPNA5,LRP5,MAPK8,MCOLN3,MOSPD2,NEURL1,NIPAL3,NPR3,PDZD3,PEX14,PPARG,RAB1B,RIN3,RPL14,RPL15,RPL18A,RPL27,RPL27A,RPL34,RPL35A,RPL5,RPL6,RPL7,RPL8,RPL9,RPS11,RPS13,RPS14,RPS2,RPS8,RRBP1,RXRA,S100A8,SIRPB1,SLC22A23,SLC26A4,SLC39A10,SLC44A1,SLCO1C1,SNAP29,SPTAN1,SRSF5,VAMP4 |
| GO:0009059 | macromolecule biosynthetic process | 61 | 3576 | 0.0086 | ENSP00000210060,ENSP00000211936,ENSP00000222247,ENSP00000223273,ENSP00000233242,ENSP00000237853,ENSP00000240185,ENSP00000244769,ENSP00000250003,ENSP00000252602,ENSP00000253099,ENSP00000259056,ENSP00000262584,ENSP00000270625,ENSP00000273317,ENSP00000287820,ENSP00000291547,ENSP00000293860,ENSP00000296953,ENSP00000300291,ENSP00000309334,ENSP00000312631,ENSP00000316335,ENSP00000324463,ENSP00000327025,ENSP00000339795,ENSP00000341885,ENSP00000344260,ENSP00000345917,ENSP00000346015,ENSP00000353910,ENSP00000354518,ENSP00000356331,ENSP00000359345,ENSP00000367038,ENSP00000368927,ENSP00000376534,ENSP00000377836,ENSP00000378163,ENSP00000379506,ENSP00000379888,ENSP00000384000,ENSP00000384371,ENSP00000385958,ENSP00000389176,ENSP00000393795,ENSP00000394085,ENSP00000395007,ENSP00000400467,ENSP00000403172,ENSP00000413929,ENSP00000419117,ENSP00000419692,ENSP00000420095,ENSP00000428340,ENSP00000430432,ENSP00000435777,ENSP00000440864,ENSP00000451261,ENSP00000464813,ENSP00000484288 | ANGPT1,APOB,ATXN1,BAZ2B,BCL6,C1GALT1,CREBRF,DHPS,EIF1AX,ELL2,FARS2,FUT8,GALNT15,GALNT5,GPC3,HDAC2,INSM1,JMJD6,LIG4,LIMD1,LYAR,MRPL34,MRPL4,MYOD1,NR4A1,NR5A2,NUDT21,PKNOX1,POLR3K,PPARG,RCOR3,RORC,RPL14,RPL15,RPL18A,RPL27,RPL27A,RPL34,RPL35A,RPL5,RPL6,RPL7,RPL8,RPL9,RPS11,RPS13,RPS14,RPS2,RPS8,RRBP1,RXRA,TARDBP,UBE2J1,YAE1D1,YLPM1,ZMYND8,ZNF184,ZNF512B,ZNF681,ZNF746,ZNF830 |
| GO:0016070 | RNA metabolic process | 59 | 3430 | 0.0086 | ENSP00000211936,ENSP00000221233,ENSP00000222247,ENSP00000230859,ENSP00000236273,ENSP00000237853,ENSP00000240185,ENSP00000244769,ENSP00000250003,ENSP00000262584,ENSP00000265564,ENSP00000270625,ENSP00000273317,ENSP00000287820,ENSP00000291547,ENSP00000293860,ENSP00000296953,ENSP00000300291,ENSP00000309334,ENSP00000312631,ENSP00000316335,ENSP00000324463,ENSP00000325074,ENSP00000327025,ENSP00000339795,ENSP00000341885,ENSP00000345917,ENSP00000346015,ENSP00000354518,ENSP00000356331,ENSP00000359345,ENSP00000361433,ENSP00000366843,ENSP00000373715,ENSP00000376534,ENSP00000378163,ENSP00000379506,ENSP00000379888,ENSP00000384000,ENSP00000384371,ENSP00000384792,ENSP00000385958,ENSP00000393795,ENSP00000394085,ENSP00000395007,ENSP00000400467,ENSP00000403172,ENSP00000413929,ENSP00000418563,ENSP00000419117,ENSP00000419692,ENSP00000420095,ENSP00000423067,ENSP00000430432,ENSP00000435777,ENSP00000440864,ENSP00000443926,ENSP00000452123,ENSP00000464813 | ATXN1,ATXN2,BAZ2B,BCL6,CELF2,CREBRF,DCP2,ELL2,EXOSC2,EXOSC5,EXOSC7,FARS2,HDAC2,INSM1,JMJD6,LIMD1,LYAR,MYOD1,NR4A1,NR5A2,NUDT21,PAPD7,PKNOX1,POLR3K,PPARG,PPP2R2A,RCOR3,RORC,RPL14,RPL15,RPL18A,RPL27,RPL27A,RPL34,RPL35A,RPL5,RPL6,RPL7,RPL8,RPL9,RPS11,RPS13,RPS14,RPS2,RPS8,RXRA,SRSF5,SYF2,TARDBP,U2SURP,WDR36,WDR62,YLPM1,ZMYND8,ZNF184,ZNF512B,ZNF681,ZNF746,ZNF830 |
| GO:0034660 | ncRNA metabolic process | 16 | 497 | 0.0086 | ENSP00000221233,ENSP00000237853,ENSP00000265564,ENSP00000293860,ENSP00000316335,ENSP00000339795,ENSP00000341885,ENSP00000345917,ENSP00000359345,ENSP00000361433,ENSP00000379506,ENSP00000379888,ENSP00000385958,ENSP00000419117,ENSP00000423067,ENSP00000464813 | ELL2,EXOSC2,EXOSC5,EXOSC7,FARS2,LYAR,POLR3K,RPL14,RPL27,RPL35A,RPL5,RPL7,RPS14,RPS2,RPS8,WDR36 |
| GO:0042273 | ribosomal large subunit biogenesis | 6 | 68 | 0.0086 | ENSP00000223273,ENSP00000339795,ENSP00000359345,ENSP00000379506,ENSP00000403172,ENSP00000419117 | RPL14,RPL35A,RPL5,RPL6,RPL7,YAE1D1 |
| GO:0051172 | negative regulation of nitrogen compound metabolic process | 44 | 2307 | 0.0086 | ENSP00000229239,ENSP00000240185,ENSP00000244769,ENSP00000273317,ENSP00000274306,ENSP00000276431,ENSP00000280154,ENSP00000285670,ENSP00000286234,ENSP00000287820,ENSP00000294304,ENSP00000296953,ENSP00000299084,ENSP00000303427,ENSP00000312631,ENSP00000320176,ENSP00000323696,ENSP00000327025,ENSP00000341680,ENSP00000345917,ENSP00000347742,ENSP00000349016,ENSP00000354518,ENSP00000356042,ENSP00000359345,ENSP00000364145,ENSP00000364212,ENSP00000373715,ENSP00000377836,ENSP00000384371,ENSP00000385958,ENSP00000387122,ENSP00000395007,ENSP00000413929,ENSP00000419692,ENSP00000420095,ENSP00000428340,ENSP00000430432,ENSP00000435777,ENSP00000451261,ENSP00000469880,ENSP00000473631,ENSP00000475814,ENSP00000483552 | ANGPT1,ATXN1,BCL6,CDA,CLEC16A,CREBRF,CRTAP,DCP2,DEPTOR,DNAJC3,DTNBP1,EZR,FURIN,GAPDH,GPC3,GZMA,HCLS1,HDAC2,HIST2H2AA3,INSM1,LIMD1,LRP5,LYAR,PDCD4,PDS5A,PDZD3,PEX14,PFKFB1,PPARG,RCOR3,RORC,RPL5,RPS13,RPS14,RXRA,SAMSN1,SPRED1,TARDBP,TNFRSF10B,UBE2J1,VRK3,ZMYND8,ZNF746,ZNF830 |
| GO:0043170 | macromolecule metabolic process | 107 | 7453 | 0.0099 | ENSP00000044462,ENSP00000210060,ENSP00000211936,ENSP00000216455,ENSP00000221233,ENSP00000222247,ENSP00000223273,ENSP00000225719,ENSP00000229239,ENSP00000230859,ENSP00000233242,ENSP00000236273,ENSP00000237853,ENSP00000238616,ENSP00000240185,ENSP00000244769,ENSP00000250003,ENSP00000252602,ENSP00000253099,ENSP00000259056,ENSP00000261427,ENSP00000262584,ENSP00000265564,ENSP00000265981,ENSP00000270625,ENSP00000273317,ENSP00000274026,ENSP00000274306,ENSP00000287820,ENSP00000291547,ENSP00000293860,ENSP00000296953,ENSP00000299084,ENSP00000300289,ENSP00000300291,ENSP00000303427,ENSP00000305976,ENSP00000306461,ENSP00000309334,ENSP00000310226,ENSP00000312631,ENSP00000316335,ENSP00000323696,ENSP00000324463,ENSP00000325074,ENSP00000327025,ENSP00000330572,ENSP00000339795,ENSP00000341885,ENSP00000344260,ENSP00000345917,ENSP00000346015,ENSP00000348308,ENSP00000349016,ENSP00000353910,ENSP00000354518,ENSP00000356042,ENSP00000356331,ENSP00000356399,ENSP00000357722,ENSP00000358795,ENSP00000359345,ENSP00000361014,ENSP00000361433,ENSP00000361540,ENSP00000361824,ENSP00000366843,ENSP00000367038,ENSP00000368927,ENSP00000373715,ENSP00000376534,ENSP00000377836,ENSP00000378163,ENSP00000378974,ENSP00000379506,ENSP00000379888,ENSP00000380178,ENSP00000384000,ENSP00000384371,ENSP00000384792,ENSP00000385958,ENSP00000389176,ENSP00000393795,ENSP00000394085,ENSP00000395007,ENSP00000400467,ENSP00000401802,ENSP00000403172,ENSP00000413929,ENSP00000418563,ENSP00000419117,ENSP00000419692,ENSP00000420095,ENSP00000423067,ENSP00000428340,ENSP00000430432,ENSP00000435777,ENSP00000440864,ENSP00000443824,ENSP00000443926,ENSP00000451261,ENSP00000452123,ENSP00000464813,ENSP00000469880,ENSP00000473631,ENSP00000483552,ENSP00000484288 | ANGPT1,APOB,ATXN1,ATXN2,BAZ2B,BCL6,C1GALT1,CCNA2,CDC20,CELF2,CFH,CPD,CREBRF,CRTAP,DCP2,DHPS,DNAJC3,EIF1AX,ELL2,EXOSC2,EXOSC5,EXOSC7,EZR,FARS2,FURIN,FUT8,GALNT15,GALNT5,GAPDH,GPC3,GZMA,HDAC2,INSM1,JMJD6,LIG4,LIMD1,LYAR,MAPK8,MAPK8IP2,MKNK1,MMP23B,MRPL34,MRPL4,MYOD1,NEK9,NEURL1,NR4A1,NR5A2,NUDT21,PAPD7,PDIA3,PDS5A,PEX14,PKNOX1,POLR3K,PPARG,PPP2R2A,PSMA3,PSMA4,PSMC6,RAB1B,RCOR3,RNF141,RORC,RPL14,RPL15,RPL18A,RPL27,RPL27A,RPL34,RPL35A,RPL5,RPL6,RPL7,RPL8,RPL9,RPS11,RPS13,RPS14,RPS2,RPS8,RRBP1,RXRA,S100A8,SIK2,SPRED1,SPRR1B,SPTAN1,SRSF5,SYF2,TARDBP,U2SURP,UBE2G1,UBE2J1,UBE2K,VRK3,WDR36,WDR62,YAE1D1,YLPM1,ZMYND8,ZNF184,ZNF512B,ZNF681,ZNF746,ZNF830,ZNRF3 |
| GO:0034475 | U4 snRNA 3'-end processing | 3 | 8 | 0.0103 | ENSP00000221233,ENSP00000265564,ENSP00000361433 | EXOSC2,EXOSC5,EXOSC7 |
| GO:0030218 | erythrocyte differentiation | 6 | 76 | 0.0136 | ENSP00000291547,ENSP00000320176,ENSP00000345917,ENSP00000384371,ENSP00000385958,ENSP00000394085 | BCL6,HCLS1,JMJD6,LYAR,PKNOX1,RPS14 |
| GO:0034645 | cellular macromolecule biosynthetic process | 59 | 3518 | 0.0157 | ENSP00000210060,ENSP00000211936,ENSP00000222247,ENSP00000223273,ENSP00000233242,ENSP00000237853,ENSP00000240185,ENSP00000244769,ENSP00000250003,ENSP00000252602,ENSP00000253099,ENSP00000259056,ENSP00000262584,ENSP00000270625,ENSP00000273317,ENSP00000287820,ENSP00000291547,ENSP00000293860,ENSP00000296953,ENSP00000300291,ENSP00000309334,ENSP00000312631,ENSP00000316335,ENSP00000324463,ENSP00000327025,ENSP00000339795,ENSP00000341885,ENSP00000344260,ENSP00000345917,ENSP00000346015,ENSP00000353910,ENSP00000354518,ENSP00000356331,ENSP00000359345,ENSP00000367038,ENSP00000368927,ENSP00000376534,ENSP00000378163,ENSP00000379506,ENSP00000379888,ENSP00000384000,ENSP00000384371,ENSP00000385958,ENSP00000389176,ENSP00000393795,ENSP00000394085,ENSP00000395007,ENSP00000400467,ENSP00000403172,ENSP00000413929,ENSP00000419117,ENSP00000419692,ENSP00000420095,ENSP00000430432,ENSP00000435777,ENSP00000440864,ENSP00000451261,ENSP00000464813,ENSP00000484288 | APOB,ATXN1,BAZ2B,BCL6,C1GALT1,CREBRF,DHPS,EIF1AX,ELL2,FARS2,FUT8,GALNT15,GALNT5,HDAC2,INSM1,JMJD6,LIG4,LIMD1,LYAR,MRPL34,MRPL4,MYOD1,NR4A1,NR5A2,NUDT21,PKNOX1,POLR3K,PPARG,RCOR3,RORC,RPL14,RPL15,RPL18A,RPL27,RPL27A,RPL34,RPL35A,RPL5,RPL6,RPL7,RPL8,RPL9,RPS11,RPS13,RPS14,RPS2,RPS8,RRBP1,RXRA,TARDBP,UBE2J1,YAE1D1,YLPM1,ZMYND8,ZNF184,ZNF512B,ZNF681,ZNF746,ZNF830 |
| GO:0031324 | negative regulation of cellular metabolic process | 45 | 2463 | 0.0167 | ENSP00000229239,ENSP00000240185,ENSP00000244769,ENSP00000273317,ENSP00000274306,ENSP00000276431,ENSP00000280154,ENSP00000285670,ENSP00000286234,ENSP00000287820,ENSP00000294304,ENSP00000296953,ENSP00000299084,ENSP00000303427,ENSP00000312631,ENSP00000320176,ENSP00000323696,ENSP00000327025,ENSP00000341680,ENSP00000345917,ENSP00000347742,ENSP00000349016,ENSP00000354518,ENSP00000356042,ENSP00000359345,ENSP00000364145,ENSP00000364212,ENSP00000366843,ENSP00000373715,ENSP00000377836,ENSP00000384371,ENSP00000385958,ENSP00000387122,ENSP00000395007,ENSP00000413929,ENSP00000419692,ENSP00000420095,ENSP00000428340,ENSP00000430432,ENSP00000435777,ENSP00000451261,ENSP00000469880,ENSP00000473631,ENSP00000475814,ENSP00000483552 | ANGPT1,ATXN1,ATXN2,BCL6,CDA,CLEC16A,CREBRF,CRTAP,DCP2,DEPTOR,DNAJC3,DTNBP1,EZR,FURIN,GAPDH,GPC3,GZMA,HCLS1,HDAC2,HIST2H2AA3,INSM1,LIMD1,LRP5,LYAR,PDCD4,PDS5A,PDZD3,PEX14,PFKFB1,PPARG,RCOR3,RORC,RPL5,RPS13,RPS14,RXRA,SAMSN1,SPRED1,TARDBP,TNFRSF10B,UBE2J1,VRK3,ZMYND8,ZNF746,ZNF830 |
| GO:0034427 | nuclear-transcribed mRNA catabolic process, exonucleolytic, 3'-5' | 3 | 10 | 0.0167 | ENSP00000221233,ENSP00000265564,ENSP00000361433 | EXOSC2,EXOSC5,EXOSC7 |
| GO:0044271 | cellular nitrogen compound biosynthetic process | 59 | 3528 | 0.0167 | ENSP00000210060,ENSP00000211936,ENSP00000222247,ENSP00000223273,ENSP00000229239,ENSP00000237853,ENSP00000240185,ENSP00000244769,ENSP00000250003,ENSP00000252602,ENSP00000253099,ENSP00000256578,ENSP00000260600,ENSP00000262584,ENSP00000270625,ENSP00000273317,ENSP00000287820,ENSP00000291547,ENSP00000293860,ENSP00000296953,ENSP00000300291,ENSP00000309334,ENSP00000312631,ENSP00000316335,ENSP00000324463,ENSP00000327025,ENSP00000339795,ENSP00000341885,ENSP00000345917,ENSP00000346015,ENSP00000356331,ENSP00000359345,ENSP00000364145,ENSP00000364212,ENSP00000367038,ENSP00000368927,ENSP00000369519,ENSP00000376534,ENSP00000378163,ENSP00000379506,ENSP00000379888,ENSP00000384000,ENSP00000384371,ENSP00000385958,ENSP00000393795,ENSP00000394085,ENSP00000395007,ENSP00000400467,ENSP00000403172,ENSP00000413929,ENSP00000419117,ENSP00000419692,ENSP00000420095,ENSP00000430432,ENSP00000435777,ENSP00000440864,ENSP00000464813,ENSP00000483552,ENSP00000484288 | ADCY3,AMPD2,ATXN1,BAZ2B,BCL6,CDA,CREBRF,DHPS,EIF1AX,ELL2,FARS2,FURIN,GAPDH,HDAC2,INSM1,JMJD6,LIG4,LIMD1,LYAR,MRPL34,MRPL4,MTAP,MYOD1,NR4A1,NR5A2,NUDT21,PFKFB1,PKNOX1,POLR3K,PPARG,RCOR3,RORC,RPL14,RPL15,RPL18A,RPL27,RPL27A,RPL34,RPL35A,RPL5,RPL6,RPL7,RPL8,RPL9,RPS11,RPS13,RPS14,RPS2,RPS8,RRBP1,RXRA,TARDBP,YAE1D1,YLPM1,ZMYND8,ZNF184,ZNF512B,ZNF681,ZNF746 |
| GO:0051179 | localization | 80 | 5233 | 0.0167 | ENSP00000215730,ENSP00000216487,ENSP00000222247,ENSP00000233242,ENSP00000236192,ENSP00000244769,ENSP00000246554,ENSP00000260600,ENSP00000262584,ENSP00000265074,ENSP00000265715,ENSP00000270625,ENSP00000273317,ENSP00000276431,ENSP00000287490,ENSP00000287820,ENSP00000294304,ENSP00000296140,ENSP00000306185,ENSP00000309334,ENSP00000310226,ENSP00000310557,ENSP00000327716,ENSP00000339795,ENSP00000341680,ENSP00000341885,ENSP00000346015,ENSP00000346151,ENSP00000346931,ENSP00000347742,ENSP00000348965,ENSP00000349016,ENSP00000349456,ENSP00000353910,ENSP00000356042,ENSP00000357552,ENSP00000357722,ENSP00000358795,ENSP00000359170,ENSP00000359345,ENSP00000359621,ENSP00000361433,ENSP00000361824,ENSP00000362146,ENSP00000363298,ENSP00000363520,ENSP00000363852,ENSP00000364212,ENSP00000365477,ENSP00000365606,ENSP00000366843,ENSP00000367038,ENSP00000369860,ENSP00000371018,ENSP00000371472,ENSP00000373734,ENSP00000378163,ENSP00000378974,ENSP00000379506,ENSP00000379888,ENSP00000384371,ENSP00000385028,ENSP00000385958,ENSP00000386766,ENSP00000394085,ENSP00000400467,ENSP00000403172,ENSP00000409544,ENSP00000411528,ENSP00000419117,ENSP00000419692,ENSP00000428340,ENSP00000435777,ENSP00000440864,ENSP00000444149,ENSP00000452123,ENSP00000464813,ENSP00000473631,ENSP00000479322,ENSP00000483552 | ADCY3,ANGPT1,ANTXR2,APOB,ATXN1,ATXN2,BCL6,BSPRY,C6orf120,CCDC93,CCR1,CDA,CDCA8,CLCC1,CLDN4,CLIP1,COX6A2,COX6B1,DENND5B,DNAJC3,DPCD,DTNBP1,DYNC1H1,EXOSC2,EZR,FURIN,FUT8,GJC1,GRIPAP1,JMJD6,KCNE3,KPNA5,LIMD1,LRP5,MAGI2,MAPK8,MCOLN3,MOSPD2,NEURL1,NIN,NIPAL3,NPR3,NR4A1,PDZD3,PEX14,PPARG,RAB1B,RIN3,RPL14,RPL15,RPL18A,RPL27,RPL27A,RPL34,RPL35A,RPL5,RPL6,RPL7,RPL8,RPL9,RPS11,RPS13,RPS14,RPS2,RPS8,RRBP1,RXRA,S100A8,SIRPB1,SLC22A23,SLC26A4,SLC39A10,SLC44A1,SLCO1C1,SNAP29,SPTAN1,SRSF5,TNFRSF10B,VAMP4,WRB |
| GO:0031125 | rRNA 3'-end processing | 3 | 11 | 0.0197 | ENSP00000221233,ENSP00000265564,ENSP00000361433 | EXOSC2,EXOSC5,EXOSC7 |
| GO:0043628 | ncRNA 3'-end processing | 4 | 30 | 0.0222 | ENSP00000221233,ENSP00000265564,ENSP00000293860,ENSP00000361433 | EXOSC2,EXOSC5,EXOSC7,POLR3K |
| GO:0044237 | cellular metabolic process | 120 | 8797 | 0.0222 | ENSP00000044462,ENSP00000210060,ENSP00000211936,ENSP00000215730,ENSP00000216455,ENSP00000221233,ENSP00000222247,ENSP00000223273,ENSP00000225719,ENSP00000229239,ENSP00000230859,ENSP00000233242,ENSP00000236273,ENSP00000237853,ENSP00000238616,ENSP00000240185,ENSP00000244769,ENSP00000246554,ENSP00000250003,ENSP00000252602,ENSP00000253099,ENSP00000256578,ENSP00000258317,ENSP00000259056,ENSP00000260600,ENSP00000261427,ENSP00000262584,ENSP00000265564,ENSP00000265981,ENSP00000270625,ENSP00000273317,ENSP00000274026,ENSP00000274306,ENSP00000287490,ENSP00000287820,ENSP00000291547,ENSP00000293860,ENSP00000296953,ENSP00000299084,ENSP00000300291,ENSP00000303427,ENSP00000304642,ENSP00000305976,ENSP00000306461,ENSP00000309334,ENSP00000310226,ENSP00000312631,ENSP00000316335,ENSP00000323696,ENSP00000324463,ENSP00000325074,ENSP00000327025,ENSP00000330572,ENSP00000339795,ENSP00000341885,ENSP00000344260,ENSP00000345917,ENSP00000346015,ENSP00000349016,ENSP00000353910,ENSP00000354518,ENSP00000356042,ENSP00000356331,ENSP00000357722,ENSP00000358795,ENSP00000359345,ENSP00000361014,ENSP00000361433,ENSP00000361540,ENSP00000361824,ENSP00000363852,ENSP00000364145,ENSP00000364212,ENSP00000366843,ENSP00000367038,ENSP00000368927,ENSP00000369519,ENSP00000373715,ENSP00000376534,ENSP00000377836,ENSP00000378163,ENSP00000378974,ENSP00000379506,ENSP00000379888,ENSP00000380178,ENSP00000384000,ENSP00000384371,ENSP00000384792,ENSP00000385080,ENSP00000385958,ENSP00000387122,ENSP00000389176,ENSP00000393795,ENSP00000394085,ENSP00000395007,ENSP00000400467,ENSP00000401802,ENSP00000403172,ENSP00000413929,ENSP00000418287,ENSP00000418563,ENSP00000419117,ENSP00000419692,ENSP00000420095,ENSP00000423067,ENSP00000425487,ENSP00000428340,ENSP00000430432,ENSP00000435777,ENSP00000440864,ENSP00000443824,ENSP00000443926,ENSP00000451261,ENSP00000452123,ENSP00000464813,ENSP00000469880,ENSP00000473631,ENSP00000480571,ENSP00000483552,ENSP00000484288 | ADCY3,AMPD2,ANGPT1,APOB,ATXN1,ATXN2,BAZ2B,BCL6,C1GALT1,CCNA2,CDA,CDC20,CELF2,CIAO1,CLEC16A,COX6A2,COX6B1,CPD,CREBRF,CRTAP,CYP3A7,DCP2,DHPS,DNAJC3,EIF1AX,ELL2,EXOSC2,EXOSC5,EXOSC7,EZR,FAH,FAM134B,FARS2,FURIN,FUT8,GALNT15,GALNT5,GAPDH,GPC3,GZMA,HDAC2,INPP4B,INSM1,JMJD6,LIG4,LIMD1,LYAR,MAPK8,MAPK8IP2,MKNK1,MRPL34,MRPL4,MTAP,MYOD1,NEK9,NEURL1,NPL,NR4A1,NR5A2,NUDT21,PAPD7,PDS5A,PEX14,PFKFB1,PKNOX1,POLR3K,PPARG,PPP2R2A,PSMA3,PSMA4,PSMC6,RAB1B,RCOR3,RNF141,RORC,RPL14,RPL15,RPL18A,RPL27,RPL27A,RPL34,RPL35A,RPL5,RPL6,RPL7,RPL8,RPL9,RPS11,RPS13,RPS14,RPS2,RPS8,RRBP1,RXRA,S100A8,SIK2,SLC44A1,SNAP29,SPRED1,SPRR1B,SPTAN1,SRSF5,SYF2,TARDBP,U2SURP,UBE2G1,UBE2J1,UBE2K,VRK3,WDR36,WDR62,YAE1D1,YLPM1,ZMYND8,ZNF184,ZNF512B,ZNF681,ZNF746,ZNF830,ZNRF3 |
| GO:0022618 | ribonucleoprotein complex assembly | 9 | 203 | 0.0225 | ENSP00000273317,ENSP00000300291,ENSP00000348965,ENSP00000359345,ENSP00000366843,ENSP00000385958,ENSP00000403172,ENSP00000443926,ENSP00000452123 | ATXN2,CELF2,DYNC1H1,LIMD1,NUDT21,RPL5,RPL6,RPS14,SRSF5 |
| GO:0034641 | cellular nitrogen compound metabolic process | 78 | 5126 | 0.0225 | ENSP00000210060,ENSP00000211936,ENSP00000221233,ENSP00000222247,ENSP00000223273,ENSP00000225719,ENSP00000229239,ENSP00000230859,ENSP00000236273,ENSP00000237853,ENSP00000240185,ENSP00000244769,ENSP00000250003,ENSP00000252602,ENSP00000253099,ENSP00000256578,ENSP00000260600,ENSP00000262584,ENSP00000265564,ENSP00000270625,ENSP00000273317,ENSP00000287490,ENSP00000287820,ENSP00000291547,ENSP00000293860,ENSP00000296953,ENSP00000300291,ENSP00000303427,ENSP00000309334,ENSP00000312631,ENSP00000316335,ENSP00000324463,ENSP00000325074,ENSP00000327025,ENSP00000339795,ENSP00000341885,ENSP00000345917,ENSP00000346015,ENSP00000353910,ENSP00000354518,ENSP00000356331,ENSP00000359345,ENSP00000361433,ENSP00000363852,ENSP00000364145,ENSP00000364212,ENSP00000366843,ENSP00000367038,ENSP00000368927,ENSP00000369519,ENSP00000373715,ENSP00000376534,ENSP00000378163,ENSP00000379506,ENSP00000379888,ENSP00000384000,ENSP00000384371,ENSP00000384792,ENSP00000385958,ENSP00000393795,ENSP00000394085,ENSP00000395007,ENSP00000400467,ENSP00000403172,ENSP00000413929,ENSP00000418563,ENSP00000419117,ENSP00000419692,ENSP00000420095,ENSP00000423067,ENSP00000430432,ENSP00000435777,ENSP00000440864,ENSP00000443926,ENSP00000452123,ENSP00000464813,ENSP00000483552,ENSP00000484288 | ADCY3,AMPD2,ATXN1,ATXN2,BAZ2B,BCL6,CDA,CELF2,COX6A2,CPD,CREBRF,DCP2,DHPS,EIF1AX,ELL2,EXOSC2,EXOSC5,EXOSC7,FARS2,FURIN,FUT8,GAPDH,HDAC2,INSM1,JMJD6,LIG4,LIMD1,LYAR,MRPL34,MRPL4,MTAP,MYOD1,NR4A1,NR5A2,NUDT21,PAPD7,PDS5A,PFKFB1,PKNOX1,POLR3K,PPARG,PPP2R2A,RCOR3,RORC,RPL14,RPL15,RPL18A,RPL27,RPL27A,RPL34,RPL35A,RPL5,RPL6,RPL7,RPL8,RPL9,RPS11,RPS13,RPS14,RPS2,RPS8,RRBP1,RXRA,SLC44A1,SRSF5,SYF2,TARDBP,U2SURP,WDR36,WDR62,YAE1D1,YLPM1,ZMYND8,ZNF184,ZNF512B,ZNF681,ZNF746,ZNF830 |
| GO:0080090 | regulation of primary metabolic process | 88 | 5982 | 0.0235 | ENSP00000211936,ENSP00000216455,ENSP00000221233,ENSP00000229239,ENSP00000233242,ENSP00000237853,ENSP00000240185,ENSP00000244769,ENSP00000250003,ENSP00000261427,ENSP00000262319,ENSP00000265564,ENSP00000265981,ENSP00000273317,ENSP00000274026,ENSP00000274306,ENSP00000276431,ENSP00000280154,ENSP00000285670,ENSP00000286234,ENSP00000287820,ENSP00000291547,ENSP00000294304,ENSP00000296140,ENSP00000296953,ENSP00000299084,ENSP00000300291,ENSP00000301455,ENSP00000303427,ENSP00000310226,ENSP00000312631,ENSP00000320176,ENSP00000323074,ENSP00000323696,ENSP00000324463,ENSP00000325074,ENSP00000327025,ENSP00000330572,ENSP00000338788,ENSP00000341680,ENSP00000341885,ENSP00000345917,ENSP00000346151,ENSP00000347742,ENSP00000349016,ENSP00000354518,ENSP00000356042,ENSP00000356331,ENSP00000356399,ENSP00000357722,ENSP00000358795,ENSP00000359345,ENSP00000359859,ENSP00000361014,ENSP00000361433,ENSP00000361540,ENSP00000364145,ENSP00000364212,ENSP00000366843,ENSP00000373715,ENSP00000376534,ENSP00000377836,ENSP00000378974,ENSP00000384000,ENSP00000384371,ENSP00000385958,ENSP00000386184,ENSP00000386766,ENSP00000387122,ENSP00000393795,ENSP00000394085,ENSP00000395007,ENSP00000401802,ENSP00000403172,ENSP00000413929,ENSP00000418287,ENSP00000419692,ENSP00000420095,ENSP00000428340,ENSP00000430432,ENSP00000435777,ENSP00000440864,ENSP00000451261,ENSP00000452123,ENSP00000469880,ENSP00000473631,ENSP00000475814,ENSP00000483552 | ANGPT1,ANGPTL4,APOB,ATXN1,ATXN2,BAZ2B,BCL6,CCNA2,CCR1,CDA,CDC20,CFH,CIAO1,CLEC16A,CREBRF,CRTAP,DCP2,DEPTOR,DNAJC3,DTNBP1,ELL2,EXOSC2,EXOSC5,EXOSC7,EZR,FURIN,GAPDH,GPC3,GZMA,HCLS1,HDAC2,HIST2H2AA3,INSM1,JMJD6,LIMD1,LRP5,LYAR,MAGI2,MAPK8,MAPK8IP2,MKNK1,MTG2,MYOD1,NEURL1,NR4A1,NR5A2,NUDT21,PDCD4,PDS5A,PDZD3,PEX14,PFKFB1,PKNOX1,PPARG,PPP2R2A,PSMA3,PSMC6,RAB1B,RAI1,RCOR3,RNF141,RORC,RPL5,RPL6,RPS13,RPS14,RPS2,RXRA,S100A8,SAMSN1,SLC39A10,SPRED1,SRSF5,TARDBP,TELO2,TNFRSF10B,UBE2J1,UBE2K,VRK3,YLPM1,ZBED8,ZC3H15,ZMYND8,ZNF184,ZNF512B,ZNF681,ZNF746,ZNF830 |
| GO:0051171 | regulation of nitrogen compound metabolic process | 86 | 5827 | 0.0254 | ENSP00000211936,ENSP00000216455,ENSP00000221233,ENSP00000229239,ENSP00000237853,ENSP00000240185,ENSP00000244769,ENSP00000250003,ENSP00000261427,ENSP00000262319,ENSP00000265564,ENSP00000265981,ENSP00000273317,ENSP00000274026,ENSP00000274306,ENSP00000276431,ENSP00000280154,ENSP00000285670,ENSP00000286234,ENSP00000287820,ENSP00000291547,ENSP00000294304,ENSP00000296140,ENSP00000296953,ENSP00000299084,ENSP00000300291,ENSP00000303427,ENSP00000310226,ENSP00000312631,ENSP00000320176,ENSP00000323074,ENSP00000323696,ENSP00000324463,ENSP00000325074,ENSP00000327025,ENSP00000330572,ENSP00000338788,ENSP00000341680,ENSP00000341885,ENSP00000345917,ENSP00000346151,ENSP00000347742,ENSP00000349016,ENSP00000354518,ENSP00000356042,ENSP00000356331,ENSP00000356399,ENSP00000357722,ENSP00000358795,ENSP00000359345,ENSP00000359859,ENSP00000361014,ENSP00000361433,ENSP00000361540,ENSP00000364145,ENSP00000364212,ENSP00000366843,ENSP00000373715,ENSP00000376534,ENSP00000377836,ENSP00000378974,ENSP00000384000,ENSP00000384371,ENSP00000385958,ENSP00000386184,ENSP00000386766,ENSP00000387122,ENSP00000393795,ENSP00000394085,ENSP00000395007,ENSP00000401802,ENSP00000403172,ENSP00000413929,ENSP00000418287,ENSP00000419692,ENSP00000420095,ENSP00000428340,ENSP00000430432,ENSP00000435777,ENSP00000440864,ENSP00000451261,ENSP00000452123,ENSP00000469880,ENSP00000473631,ENSP00000475814,ENSP00000483552 | ANGPT1,ATXN1,ATXN2,BAZ2B,BCL6,CCNA2,CCR1,CDA,CDC20,CFH,CIAO1,CLEC16A,CREBRF,CRTAP,DCP2,DEPTOR,DNAJC3,DTNBP1,ELL2,EXOSC2,EXOSC5,EXOSC7,EZR,FURIN,GAPDH,GPC3,GZMA,HCLS1,HDAC2,HIST2H2AA3,INSM1,JMJD6,LIMD1,LRP5,LYAR,MAGI2,MAPK8,MAPK8IP2,MKNK1,MTG2,MYOD1,NEURL1,NR4A1,NR5A2,NUDT21,PDCD4,PDS5A,PDZD3,PEX14,PFKFB1,PKNOX1,PPARG,PPP2R2A,PSMA3,PSMC6,RAB1B,RAI1,RCOR3,RNF141,RORC,RPL5,RPL6,RPS13,RPS14,RPS2,RXRA,S100A8,SAMSN1,SLC39A10,SPRED1,SRSF5,TARDBP,TELO2,TNFRSF10B,UBE2J1,UBE2K,VRK3,YLPM1,ZBED8,ZC3H15,ZMYND8,ZNF184,ZNF512B,ZNF681,ZNF746,ZNF830 |
| GO:0015031 | protein transport | 29 | 1391 | 0.0267 | ENSP00000215730,ENSP00000222247,ENSP00000233242,ENSP00000262584,ENSP00000270625,ENSP00000287820,ENSP00000309334,ENSP00000310226,ENSP00000339795,ENSP00000341885,ENSP00000346015,ENSP00000349016,ENSP00000357552,ENSP00000359345,ENSP00000365477,ENSP00000365606,ENSP00000367038,ENSP00000378163,ENSP00000379506,ENSP00000379888,ENSP00000384371,ENSP00000385958,ENSP00000400467,ENSP00000403172,ENSP00000419117,ENSP00000435777,ENSP00000452123,ENSP00000464813,ENSP00000479322 | APOB,BCL6,CCDC93,CLIP1,GRIPAP1,KPNA5,PEX14,PPARG,RAB1B,RPL14,RPL15,RPL18A,RPL27,RPL27A,RPL34,RPL35A,RPL5,RPL6,RPL7,RPL8,RPL9,RPS11,RPS13,RPS14,RPS2,RPS8,RRBP1,SNAP29,SRSF5 |
| GO:0033962 | cytoplasmic mRNA processing body assembly | 3 | 13 | 0.0267 | ENSP00000273317,ENSP00000348965,ENSP00000366843 | ATXN2,DYNC1H1,LIMD1 |
| GO:0043928 | exonucleolytic catabolism of deadenylated mRNA | 4 | 33 | 0.0267 | ENSP00000221233,ENSP00000265564,ENSP00000361433,ENSP00000373715 | DCP2,EXOSC2,EXOSC5,EXOSC7 |
| GO:0046907 | intracellular transport | 29 | 1390 | 0.0267 | ENSP00000215730,ENSP00000222247,ENSP00000244769,ENSP00000262584,ENSP00000270625,ENSP00000309334,ENSP00000310226,ENSP00000339795,ENSP00000341680,ENSP00000341885,ENSP00000346015,ENSP00000348965,ENSP00000349016,ENSP00000357552,ENSP00000359345,ENSP00000361824,ENSP00000365606,ENSP00000378163,ENSP00000379506,ENSP00000379888,ENSP00000384371,ENSP00000385958,ENSP00000400467,ENSP00000403172,ENSP00000419117,ENSP00000435777,ENSP00000452123,ENSP00000464813,ENSP00000479322 | ATXN1,BCL6,CLIP1,DTNBP1,DYNC1H1,GRIPAP1,KPNA5,PEX14,RAB1B,RPL14,RPL15,RPL18A,RPL27,RPL27A,RPL34,RPL35A,RPL5,RPL6,RPL7,RPL8,RPL9,RPS11,RPS13,RPS14,RPS2,RPS8,SNAP29,SPTAN1,SRSF5 |
| GO:0071028 | nuclear mRNA surveillance | 3 | 13 | 0.0267 | ENSP00000221233,ENSP00000265564,ENSP00000361433 | EXOSC2,EXOSC5,EXOSC7 |
| GO:0045184 | establishment of protein localization | 30 | 1467 | 0.0273 | ENSP00000215730,ENSP00000222247,ENSP00000233242,ENSP00000262584,ENSP00000270625,ENSP00000287820,ENSP00000309334,ENSP00000310226,ENSP00000327716,ENSP00000339795,ENSP00000341885,ENSP00000346015,ENSP00000349016,ENSP00000357552,ENSP00000359345,ENSP00000365477,ENSP00000365606,ENSP00000367038,ENSP00000378163,ENSP00000379506,ENSP00000379888,ENSP00000384371,ENSP00000385958,ENSP00000400467,ENSP00000403172,ENSP00000419117,ENSP00000435777,ENSP00000452123,ENSP00000464813,ENSP00000479322 | APOB,BCL6,CCDC93,CLIP1,GRIPAP1,KPNA5,PEX14,PPARG,RAB1B,RPL14,RPL15,RPL18A,RPL27,RPL27A,RPL34,RPL35A,RPL5,RPL6,RPL7,RPL8,RPL9,RPS11,RPS13,RPS14,RPS2,RPS8,RRBP1,SNAP29,SRSF5,WRB |
| GO:0044085 | cellular component biogenesis | 45 | 2556 | 0.0287 | ENSP00000210060,ENSP00000215730,ENSP00000221233,ENSP00000223273,ENSP00000233242,ENSP00000236192,ENSP00000261427,ENSP00000265564,ENSP00000273317,ENSP00000300291,ENSP00000301455,ENSP00000310226,ENSP00000320176,ENSP00000330572,ENSP00000339795,ENSP00000341885,ENSP00000345917,ENSP00000346151,ENSP00000348965,ENSP00000349016,ENSP00000356042,ENSP00000358795,ENSP00000359345,ENSP00000359859,ENSP00000361433,ENSP00000361540,ENSP00000364212,ENSP00000366843,ENSP00000371472,ENSP00000378163,ENSP00000379506,ENSP00000379888,ENSP00000384792,ENSP00000385958,ENSP00000403172,ENSP00000411528,ENSP00000418287,ENSP00000419117,ENSP00000419692,ENSP00000423067,ENSP00000428340,ENSP00000429900,ENSP00000443926,ENSP00000452123,ENSP00000464813 | ANGPT1,ANGPTL4,APOB,ASAP1,ATXN2,CDA,CDC20,CELF2,CIAO1,DHPS,DYNC1H1,EXOSC2,EXOSC5,EXOSC7,EZR,GJC1,HCLS1,LIMD1,LYAR,MAGI2,MAPK8IP2,MTG2,NEURL1,NIN,NUDT21,PEX14,RAB1B,RPL14,RPL27,RPL34,RPL35A,RPL5,RPL6,RPL7,RPS14,RPS2,RPS8,RXRA,SNAP29,SRSF5,UBE2K,VAMP4,WDR36,WDR62,YAE1D1 |
| GO:0006807 | nitrogen compound metabolic process | 114 | 8349 | 0.0305 | ENSP00000044462,ENSP00000210060,ENSP00000211936,ENSP00000216455,ENSP00000221233,ENSP00000222247,ENSP00000223273,ENSP00000225719,ENSP00000229239,ENSP00000230859,ENSP00000233242,ENSP00000236273,ENSP00000237853,ENSP00000238616,ENSP00000240185,ENSP00000244769,ENSP00000250003,ENSP00000252602,ENSP00000253099,ENSP00000256578,ENSP00000259056,ENSP00000260600,ENSP00000261427,ENSP00000262584,ENSP00000265564,ENSP00000265981,ENSP00000270625,ENSP00000273317,ENSP00000274026,ENSP00000274306,ENSP00000287490,ENSP00000287820,ENSP00000291547,ENSP00000293860,ENSP00000296953,ENSP00000299084,ENSP00000300289,ENSP00000300291,ENSP00000303427,ENSP00000305976,ENSP00000306461,ENSP00000309334,ENSP00000310226,ENSP00000312631,ENSP00000316335,ENSP00000323696,ENSP00000324463,ENSP00000325074,ENSP00000327025,ENSP00000330572,ENSP00000339795,ENSP00000341885,ENSP00000344260,ENSP00000345917,ENSP00000346015,ENSP00000348308,ENSP00000349016,ENSP00000353910,ENSP00000354518,ENSP00000356331,ENSP00000356399,ENSP00000357722,ENSP00000358795,ENSP00000359345,ENSP00000361014,ENSP00000361433,ENSP00000361540,ENSP00000361824,ENSP00000363852,ENSP00000364145,ENSP00000364212,ENSP00000366843,ENSP00000367038,ENSP00000368927,ENSP00000369519,ENSP00000373715,ENSP00000376534,ENSP00000377836,ENSP00000378163,ENSP00000378974,ENSP00000379506,ENSP00000379888,ENSP00000380178,ENSP00000384000,ENSP00000384371,ENSP00000384792,ENSP00000385080,ENSP00000385958,ENSP00000389176,ENSP00000393795,ENSP00000394085,ENSP00000395007,ENSP00000400467,ENSP00000401802,ENSP00000403172,ENSP00000413929,ENSP00000418563,ENSP00000419117,ENSP00000419692,ENSP00000420095,ENSP00000423067,ENSP00000428340,ENSP00000430432,ENSP00000435777,ENSP00000440864,ENSP00000443824,ENSP00000443926,ENSP00000451261,ENSP00000452123,ENSP00000464813,ENSP00000469880,ENSP00000473631,ENSP00000483552,ENSP00000484288 | ADCY3,AMPD2,ANGPT1,APOB,ATXN1,ATXN2,BAZ2B,BCL6,C1GALT1,CCNA2,CDA,CDC20,CELF2,CFH,COX6A2,CPD,CREBRF,CRTAP,DCP2,DHPS,DNAJC3,EIF1AX,ELL2,EXOSC2,EXOSC5,EXOSC7,FAH,FARS2,FURIN,FUT8,GALNT15,GALNT5,GAPDH,GPC3,GZMA,HDAC2,INSM1,JMJD6,LIG4,LIMD1,LYAR,MAPK8,MAPK8IP2,MKNK1,MMP23B,MRPL34,MRPL4,MTAP,MYOD1,NEK9,NEURL1,NR4A1,NR5A2,NUDT21,PAPD7,PDIA3,PDS5A,PEX14,PFKFB1,PKNOX1,POLR3K,PPARG,PPP2R2A,PSMA3,PSMA4,PSMC6,RAB1B,RCOR3,RNF141,RORC,RPL14,RPL15,RPL18A,RPL27,RPL27A,RPL34,RPL35A,RPL5,RPL6,RPL7,RPL8,RPL9,RPS11,RPS13,RPS14,RPS2,RPS8,RRBP1,RXRA,S100A8,SIK2,SLC44A1,SPRED1,SPRR1B,SPTAN1,SRSF5,SYF2,TARDBP,U2SURP,UBE2G1,UBE2J1,UBE2K,VRK3,WDR36,WDR62,YAE1D1,YLPM1,ZMYND8,ZNF184,ZNF512B,ZNF681,ZNF746,ZNF830,ZNRF3 |
| GO:0033993 | response to lipid | 20 | 825 | 0.0305 | ENSP00000233242,ENSP00000250003,ENSP00000260600,ENSP00000274026,ENSP00000276431,ENSP00000280154,ENSP00000287820,ENSP00000327025,ENSP00000334448,ENSP00000356331,ENSP00000357722,ENSP00000358451,ENSP00000364145,ENSP00000369519,ENSP00000378974,ENSP00000409544,ENSP00000419692,ENSP00000430432,ENSP00000440864,ENSP00000464813 | ADCY3,APOB,CCNA2,CLDN4,GDAP2,GNG2,HDAC2,MAPK8,MTAP,MYOD1,NR4A1,NR5A2,PDCD4,PFKFB1,PPARG,RORC,RPL27,RXRA,S100A8,TNFRSF10B |
| GO:0065007 | biological regulation | 150 | 11740 | 0.0305 | ENSP00000210060,ENSP00000211936,ENSP00000215730,ENSP00000216455,ENSP00000216487,ENSP00000220507,ENSP00000221233,ENSP00000222247,ENSP00000229239,ENSP00000233242,ENSP00000236273,ENSP00000237853,ENSP00000240185,ENSP00000244769,ENSP00000250003,ENSP00000256578,ENSP00000260600,ENSP00000261427,ENSP00000262319,ENSP00000262584,ENSP00000265074,ENSP00000265564,ENSP00000265715,ENSP00000265981,ENSP00000270625,ENSP00000273317,ENSP00000274026,ENSP00000274306,ENSP00000276431,ENSP00000280154,ENSP00000285670,ENSP00000286234,ENSP00000287490,ENSP00000287820,ENSP00000290219,ENSP00000291547,ENSP00000294304,ENSP00000296140,ENSP00000296953,ENSP00000299084,ENSP00000300289,ENSP00000300291,ENSP00000301030,ENSP00000301455,ENSP00000303427,ENSP00000304642,ENSP00000305976,ENSP00000306185,ENSP00000306822,ENSP00000309334,ENSP00000310226,ENSP00000310557,ENSP00000312631,ENSP00000320176,ENSP00000323074,ENSP00000323696,ENSP00000324463,ENSP00000325074,ENSP00000327025,ENSP00000330572,ENSP00000334448,ENSP00000337946,ENSP00000338788,ENSP00000339795,ENSP00000341680,ENSP00000341838,ENSP00000341885,ENSP00000344242,ENSP00000345917,ENSP00000346015,ENSP00000346151,ENSP00000347742,ENSP00000348965,ENSP00000349016,ENSP00000350348,ENSP00000353910,ENSP00000354518,ENSP00000356042,ENSP00000356331,ENSP00000356399,ENSP00000357722,ENSP00000358795,ENSP00000359345,ENSP00000359859,ENSP00000361014,ENSP00000361433,ENSP00000361540,ENSP00000361824,ENSP00000363852,ENSP00000364145,ENSP00000364212,ENSP00000365606,ENSP00000366843,ENSP00000367038,ENSP00000369519,ENSP00000369860,ENSP00000371018,ENSP00000373715,ENSP00000373734,ENSP00000376534,ENSP00000377836,ENSP00000378163,ENSP00000378338,ENSP00000378974,ENSP00000379506,ENSP00000379888,ENSP00000380185,ENSP00000384000,ENSP00000384371,ENSP00000385958,ENSP00000386184,ENSP00000386766,ENSP00000387122,ENSP00000391524,ENSP00000392660,ENSP00000393795,ENSP00000394085,ENSP00000395007,ENSP00000400467,ENSP00000401802,ENSP00000403172,ENSP00000409544,ENSP00000411528,ENSP00000413929,ENSP00000414398,ENSP00000418287,ENSP00000419117,ENSP00000419692,ENSP00000420095,ENSP00000420211,ENSP00000423067,ENSP00000424765,ENSP00000425487,ENSP00000428340,ENSP00000429900,ENSP00000430432,ENSP00000435777,ENSP00000440864,ENSP00000443824,ENSP00000443926,ENSP00000444149,ENSP00000451261,ENSP00000452123,ENSP00000464813,ENSP00000469880,ENSP00000473631,ENSP00000475814,ENSP00000479322,ENSP00000483552,ENSP00000484288 | ADCY3,AMOTL2,AMPD2,ANGPT1,ANGPTL4,ANKRD11,ANTXR2,APOB,ARHGAP28,ASAP1,ATXN1,ATXN2,AVEN,BAZ2B,BCL6,CCNA2,CCR1,CDA,CDC20,CELF2,CFH,CIAO1,CLDN4,CLEC16A,CLIP1,COX6A2,CREBRF,CRTAP,DCP2,DENND5B,DENND6B,DEPTOR,DHPS,DNAJB11,DNAJC3,DTNBP1,DYNC1H1,ELL2,EXOSC2,EXOSC5,EXOSC7,EZR,FAM134B,FURIN,FUT8,GAPDH,GIT1,GJC1,GNG2,GPC3,GRIPAP1,GRM7,GZMA,HCLS1,HDAC2,HIST2H2AA3,IFNGR2,INPP4B,INSM1,JMJD6,KCNE3,LIG4,LIMD1,LRP5,LRRC17,LYAR,MAGI2,MAPK8,MAPK8IP2,MKNK1,MOSPD2,MTAP,MTG2,MYOD1,NEURL1,NPR3,NR4A1,NR5A2,NUDT21,PACRG,PDCD4,PDIA3,PDS5A,PDZD3,PEX14,PFKFB1,PKNOX1,PLEKHG6,PPARG,PPP2R2A,PSMA3,PSMC6,RAB1B,RAI1,RCOR3,RGL2,RHOV,RIN3,RNF141,RORC,RPL14,RPL15,RPL18A,RPL27,RPL27A,RPL34,RPL35A,RPL5,RPL6,RPL7,RPL8,RPL9,RPS11,RPS13,RPS14,RPS2,RPS8,RRBP1,RXRA,S100A8,SAMSN1,SIK2,SIRPB1,SLC26A4,SLC39A10,SLC44A1,SLCO1C1,SNAP29,SPRED1,SPTAN1,SRSF5,SYF2,TARDBP,TELO2,TNFRSF10B,TNNI3,UBE2J1,UBE2K,VRK3,WDR36,YLPM1,ZBED8,ZC3H15,ZMYND8,ZNF184,ZNF512B,ZNF681,ZNF746,ZNF830,ZNRF3 |
| GO:0090503 | RNA phosphodiester bond hydrolysis, exonucleolytic | 4 | 36 | 0.0321 | ENSP00000221233,ENSP00000265564,ENSP00000361433,ENSP00000373715 | DCP2,EXOSC2,EXOSC5,EXOSC7 |
| GO:0051246 | regulation of protein metabolic process | 46 | 2668 | 0.0343 | ENSP00000216455,ENSP00000229239,ENSP00000240185,ENSP00000261427,ENSP00000262319,ENSP00000276431,ENSP00000280154,ENSP00000285670,ENSP00000286234,ENSP00000287820,ENSP00000294304,ENSP00000296140,ENSP00000296953,ENSP00000299084,ENSP00000310226,ENSP00000312631,ENSP00000320176,ENSP00000323696,ENSP00000325074,ENSP00000330572,ENSP00000341680,ENSP00000341885,ENSP00000346151,ENSP00000356042,ENSP00000356399,ENSP00000357722,ENSP00000358795,ENSP00000359345,ENSP00000359859,ENSP00000361014,ENSP00000361540,ENSP00000366843,ENSP00000377836,ENSP00000378974,ENSP00000384371,ENSP00000385958,ENSP00000386766,ENSP00000387122,ENSP00000401802,ENSP00000419692,ENSP00000428340,ENSP00000430432,ENSP00000451261,ENSP00000469880,ENSP00000473631,ENSP00000483552 | ANGPT1,ATXN2,BCL6,CCR1,CDC20,CFH,CLEC16A,CREBRF,CRTAP,DEPTOR,DNAJC3,DTNBP1,EZR,FURIN,GAPDH,GPC3,HCLS1,HDAC2,INSM1,LRP5,MAGI2,MAPK8,MAPK8IP2,MKNK1,MTG2,NEURL1,PDCD4,PPARG,PPP2R2A,PSMA3,PSMC6,RAB1B,RPL5,RPS14,RPS2,RXRA,S100A8,SAMSN1,SLC39A10,SPRED1,TARDBP,TELO2,TNFRSF10B,UBE2J1,UBE2K,VRK3 |
| GO:0070727 | cellular macromolecule localization | 28 | 1374 | 0.038 | ENSP00000222247,ENSP00000262584,ENSP00000270625,ENSP00000309334,ENSP00000327716,ENSP00000339795,ENSP00000341885,ENSP00000346015,ENSP00000346151,ENSP00000349016,ENSP00000356042,ENSP00000357552,ENSP00000359345,ENSP00000361433,ENSP00000365606,ENSP00000378163,ENSP00000379506,ENSP00000379888,ENSP00000384371,ENSP00000385958,ENSP00000400467,ENSP00000403172,ENSP00000419117,ENSP00000428340,ENSP00000435777,ENSP00000452123,ENSP00000464813,ENSP00000479322 | ANGPT1,BCL6,CLIP1,EXOSC2,EZR,GRIPAP1,KPNA5,MAGI2,PEX14,RPL14,RPL15,RPL18A,RPL27,RPL27A,RPL34,RPL35A,RPL5,RPL6,RPL7,RPL8,RPL9,RPS11,RPS13,RPS14,RPS2,RPS8,SRSF5,WRB |
| GO:0050790 | regulation of catalytic activity | 40 | 2249 | 0.0422 | ENSP00000216455,ENSP00000216487,ENSP00000229239,ENSP00000262319,ENSP00000265074,ENSP00000274306,ENSP00000276431,ENSP00000280154,ENSP00000286234,ENSP00000287490,ENSP00000287820,ENSP00000294304,ENSP00000299084,ENSP00000301455,ENSP00000325074,ENSP00000330572,ENSP00000341680,ENSP00000341838,ENSP00000341885,ENSP00000346151,ENSP00000347742,ENSP00000350348,ENSP00000357722,ENSP00000358795,ENSP00000359345,ENSP00000361540,ENSP00000364145,ENSP00000377836,ENSP00000378338,ENSP00000378974,ENSP00000380185,ENSP00000384371,ENSP00000386766,ENSP00000392660,ENSP00000414398,ENSP00000428340,ENSP00000429900,ENSP00000469880,ENSP00000473631,ENSP00000483552 | ANGPT1,ANGPTL4,ARHGAP28,ASAP1,BCL6,CDC20,COX6A2,DEPTOR,DNAJB11,DNAJC3,DTNBP1,FURIN,GAPDH,GIT1,GPC3,GRM7,GZMA,LRP5,MAGI2,MAPK8,MAPK8IP2,NEURL1,NPR3,PDCD4,PDZD3,PFKFB1,PLEKHG6,PPARG,PPP2R2A,PSMA3,RIN3,RPL5,RPS2,S100A8,SLC39A10,SPRED1,TELO2,TNFRSF10B,TNNI3,VRK3 |
| GO:1901564 | organonitrogen compound metabolic process | 78 | 5281 | 0.0422 | ENSP00000044462,ENSP00000210060,ENSP00000216455,ENSP00000222247,ENSP00000223273,ENSP00000225719,ENSP00000229239,ENSP00000233242,ENSP00000238616,ENSP00000250003,ENSP00000252602,ENSP00000253099,ENSP00000256578,ENSP00000259056,ENSP00000260600,ENSP00000261427,ENSP00000262584,ENSP00000265981,ENSP00000270625,ENSP00000274026,ENSP00000274306,ENSP00000287490,ENSP00000287820,ENSP00000299084,ENSP00000300289,ENSP00000305976,ENSP00000306461,ENSP00000309334,ENSP00000310226,ENSP00000312631,ENSP00000316335,ENSP00000323696,ENSP00000325074,ENSP00000330572,ENSP00000339795,ENSP00000341885,ENSP00000344260,ENSP00000346015,ENSP00000348308,ENSP00000349016,ENSP00000353910,ENSP00000356399,ENSP00000357722,ENSP00000358795,ENSP00000359345,ENSP00000361014,ENSP00000361540,ENSP00000361824,ENSP00000363852,ENSP00000364145,ENSP00000364212,ENSP00000367038,ENSP00000368927,ENSP00000369519,ENSP00000377836,ENSP00000378163,ENSP00000378974,ENSP00000379506,ENSP00000379888,ENSP00000380178,ENSP00000385080,ENSP00000385958,ENSP00000389176,ENSP00000394085,ENSP00000400467,ENSP00000401802,ENSP00000403172,ENSP00000419117,ENSP00000420095,ENSP00000428340,ENSP00000430432,ENSP00000435777,ENSP00000443824,ENSP00000451261,ENSP00000464813,ENSP00000469880,ENSP00000473631,ENSP00000483552 | ADCY3,AMPD2,ANGPT1,APOB,C1GALT1,CCNA2,CDA,CDC20,CFH,COX6A2,CPD,CRTAP,DHPS,DNAJC3,EIF1AX,FAH,FARS2,FURIN,FUT8,GALNT15,GALNT5,GAPDH,GPC3,GZMA,HDAC2,INSM1,JMJD6,MAPK8,MAPK8IP2,MKNK1,MMP23B,MRPL34,MRPL4,MTAP,MYOD1,NEK9,NEURL1,PDIA3,PEX14,PFKFB1,PPARG,PPP2R2A,PSMA3,PSMA4,PSMC6,RAB1B,RNF141,RPL14,RPL15,RPL18A,RPL27,RPL27A,RPL34,RPL35A,RPL5,RPL6,RPL7,RPL8,RPL9,RPS11,RPS13,RPS14,RPS2,RPS8,RRBP1,S100A8,SIK2,SLC44A1,SPRED1,SPRR1B,SPTAN1,UBE2G1,UBE2J1,UBE2K,VRK3,YAE1D1,ZMYND8,ZNRF3 |
| GO:0044238 | primary metabolic process | 118 | 8808 | 0.0431 | ENSP00000044462,ENSP00000210060,ENSP00000211936,ENSP00000216455,ENSP00000221233,ENSP00000222247,ENSP00000223273,ENSP00000225719,ENSP00000229239,ENSP00000230859,ENSP00000233242,ENSP00000236273,ENSP00000237853,ENSP00000238616,ENSP00000240185,ENSP00000244769,ENSP00000250003,ENSP00000252602,ENSP00000253099,ENSP00000256578,ENSP00000258317,ENSP00000259056,ENSP00000260600,ENSP00000261427,ENSP00000262584,ENSP00000265564,ENSP00000265981,ENSP00000270625,ENSP00000273317,ENSP00000274026,ENSP00000274306,ENSP00000287490,ENSP00000287820,ENSP00000291547,ENSP00000293860,ENSP00000294304,ENSP00000296953,ENSP00000299084,ENSP00000300289,ENSP00000300291,ENSP00000303427,ENSP00000305976,ENSP00000306461,ENSP00000309334,ENSP00000310226,ENSP00000312631,ENSP00000316335,ENSP00000323696,ENSP00000324463,ENSP00000325074,ENSP00000327025,ENSP00000330572,ENSP00000339795,ENSP00000341885,ENSP00000344260,ENSP00000345917,ENSP00000346015,ENSP00000348308,ENSP00000349016,ENSP00000353910,ENSP00000354518,ENSP00000356331,ENSP00000356399,ENSP00000357722,ENSP00000358795,ENSP00000359345,ENSP00000361014,ENSP00000361433,ENSP00000361540,ENSP00000361824,ENSP00000363852,ENSP00000364145,ENSP00000364212,ENSP00000366843,ENSP00000367038,ENSP00000368927,ENSP00000369519,ENSP00000373715,ENSP00000376534,ENSP00000377836,ENSP00000378163,ENSP00000378974,ENSP00000379506,ENSP00000379888,ENSP00000380178,ENSP00000384000,ENSP00000384371,ENSP00000384792,ENSP00000385080,ENSP00000385958,ENSP00000389176,ENSP00000393795,ENSP00000394085,ENSP00000395007,ENSP00000400467,ENSP00000401802,ENSP00000403172,ENSP00000413929,ENSP00000418563,ENSP00000419117,ENSP00000419692,ENSP00000420095,ENSP00000423067,ENSP00000425487,ENSP00000428340,ENSP00000430432,ENSP00000435777,ENSP00000440864,ENSP00000443824,ENSP00000443926,ENSP00000451261,ENSP00000452123,ENSP00000464813,ENSP00000469880,ENSP00000473631,ENSP00000480571,ENSP00000483552,ENSP00000484288 | ADCY3,AMPD2,ANGPT1,APOB,ATXN1,ATXN2,BAZ2B,BCL6,C1GALT1,CCNA2,CDA,CDC20,CELF2,CFH,COX6A2,CPD,CREBRF,CRTAP,CYP3A7,DCP2,DHPS,DNAJC3,EIF1AX,ELL2,EXOSC2,EXOSC5,EXOSC7,FAH,FARS2,FURIN,FUT8,GALNT15,GALNT5,GAPDH,GPC3,GZMA,HDAC2,INPP4B,INSM1,JMJD6,LIG4,LIMD1,LRP5,LYAR,MAPK8,MAPK8IP2,MKNK1,MMP23B,MRPL34,MRPL4,MTAP,MYOD1,NEK9,NEURL1,NPL,NR4A1,NR5A2,NUDT21,PAPD7,PDIA3,PDS5A,PEX14,PFKFB1,PKNOX1,POLR3K,PPARG,PPP2R2A,PSMA3,PSMA4,PSMC6,RAB1B,RCOR3,RNF141,RORC,RPL14,RPL15,RPL18A,RPL27,RPL27A,RPL34,RPL35A,RPL5,RPL6,RPL7,RPL8,RPL9,RPS11,RPS13,RPS14,RPS2,RPS8,RRBP1,RXRA,S100A8,SIK2,SLC44A1,SPRED1,SPRR1B,SPTAN1,SRSF5,SYF2,TARDBP,U2SURP,UBE2G1,UBE2J1,UBE2K,VRK3,WDR36,WDR62,YAE1D1,YLPM1,ZMYND8,ZNF184,ZNF512B,ZNF681,ZNF746,ZNF830,ZNRF3 |
| GO:0006139 | nucleobase-containing compound metabolic process | 69 | 4551 | 0.0461 | ENSP00000211936,ENSP00000221233,ENSP00000222247,ENSP00000229239,ENSP00000230859,ENSP00000236273,ENSP00000237853,ENSP00000240185,ENSP00000244769,ENSP00000250003,ENSP00000256578,ENSP00000260600,ENSP00000262584,ENSP00000265564,ENSP00000270625,ENSP00000273317,ENSP00000287490,ENSP00000287820,ENSP00000291547,ENSP00000293860,ENSP00000296953,ENSP00000300291,ENSP00000303427,ENSP00000309334,ENSP00000312631,ENSP00000316335,ENSP00000324463,ENSP00000325074,ENSP00000327025,ENSP00000339795,ENSP00000341885,ENSP00000345917,ENSP00000346015,ENSP00000353910,ENSP00000354518,ENSP00000356331,ENSP00000359345,ENSP00000361433,ENSP00000364145,ENSP00000364212,ENSP00000366843,ENSP00000369519,ENSP00000373715,ENSP00000376534,ENSP00000378163,ENSP00000379506,ENSP00000379888,ENSP00000384000,ENSP00000384371,ENSP00000384792,ENSP00000385958,ENSP00000393795,ENSP00000394085,ENSP00000395007,ENSP00000400467,ENSP00000403172,ENSP00000413929,ENSP00000418563,ENSP00000419117,ENSP00000419692,ENSP00000420095,ENSP00000423067,ENSP00000430432,ENSP00000435777,ENSP00000440864,ENSP00000443926,ENSP00000452123,ENSP00000464813,ENSP00000484288 | ADCY3,AMPD2,ATXN1,ATXN2,BAZ2B,BCL6,CDA,CELF2,COX6A2,CREBRF,DCP2,ELL2,EXOSC2,EXOSC5,EXOSC7,FARS2,FUT8,GAPDH,HDAC2,INSM1,JMJD6,LIG4,LIMD1,LYAR,MTAP,MYOD1,NR4A1,NR5A2,NUDT21,PAPD7,PDS5A,PFKFB1,PKNOX1,POLR3K,PPARG,PPP2R2A,RCOR3,RORC,RPL14,RPL15,RPL18A,RPL27,RPL27A,RPL34,RPL35A,RPL5,RPL6,RPL7,RPL8,RPL9,RPS11,RPS13,RPS14,RPS2,RPS8,RXRA,SRSF5,SYF2,TARDBP,U2SURP,WDR36,WDR62,YLPM1,ZMYND8,ZNF184,ZNF512B,ZNF681,ZNF746,ZNF830 |
| GO:0032516 | positive regulation of phosphoprotein phosphatase activity | 3 | 18 | 0.0467 | ENSP00000346151,ENSP00000386766,ENSP00000469880 | MAGI2,SLC39A10,VRK3 |
| GO:0045936 | negative regulation of phosphate metabolic process | 15 | 562 | 0.0482 | ENSP00000240185,ENSP00000280154,ENSP00000285670,ENSP00000286234,ENSP00000294304,ENSP00000299084,ENSP00000312631,ENSP00000341680,ENSP00000347742,ENSP00000356042,ENSP00000364145,ENSP00000364212,ENSP00000428340,ENSP00000469880,ENSP00000473631 | ANGPT1,CDA,DEPTOR,DNAJC3,DTNBP1,EZR,INSM1,LRP5,PDCD4,PDZD3,PFKFB1,SAMSN1,SPRED1,TARDBP,VRK3 |
| GO:0034976 | response to endoplasmic reticulum stress | 9 | 240 | 0.0486 | ENSP00000240185,ENSP00000261427,ENSP00000276431,ENSP00000296953,ENSP00000300289,ENSP00000401802,ENSP00000414398,ENSP00000451261,ENSP00000473631 | CREBRF,DNAJB11,DNAJC3,PDIA3,PSMC6,TARDBP,TNFRSF10B,UBE2J1,UBE2K |
| GO:0044249 | cellular biosynthetic process | 69 | 4567 | 0.0486 | ENSP00000210060,ENSP00000211936,ENSP00000222247,ENSP00000223273,ENSP00000229239,ENSP00000233242,ENSP00000237853,ENSP00000240185,ENSP00000244769,ENSP00000250003,ENSP00000252602,ENSP00000253099,ENSP00000256578,ENSP00000259056,ENSP00000260600,ENSP00000262584,ENSP00000270625,ENSP00000273317,ENSP00000287820,ENSP00000291547,ENSP00000293860,ENSP00000296953,ENSP00000300291,ENSP00000309334,ENSP00000312631,ENSP00000316335,ENSP00000324463,ENSP00000327025,ENSP00000339795,ENSP00000341885,ENSP00000344260,ENSP00000345917,ENSP00000346015,ENSP00000353910,ENSP00000354518,ENSP00000356331,ENSP00000359345,ENSP00000363852,ENSP00000364145,ENSP00000364212,ENSP00000367038,ENSP00000368927,ENSP00000369519,ENSP00000376534,ENSP00000378163,ENSP00000379506,ENSP00000379888,ENSP00000384000,ENSP00000384371,ENSP00000385958,ENSP00000389176,ENSP00000393795,ENSP00000394085,ENSP00000395007,ENSP00000400467,ENSP00000403172,ENSP00000413929,ENSP00000419117,ENSP00000419692,ENSP00000420095,ENSP00000425487,ENSP00000428340,ENSP00000430432,ENSP00000435777,ENSP00000440864,ENSP00000451261,ENSP00000464813,ENSP00000483552,ENSP00000484288 | ADCY3,AMPD2,ANGPT1,APOB,ATXN1,BAZ2B,BCL6,C1GALT1,CDA,CREBRF,DHPS,EIF1AX,ELL2,FARS2,FURIN,FUT8,GALNT15,GALNT5,GAPDH,HDAC2,INPP4B,INSM1,JMJD6,LIG4,LIMD1,LYAR,MRPL34,MRPL4,MTAP,MYOD1,NR4A1,NR5A2,NUDT21,PFKFB1,PKNOX1,POLR3K,PPARG,RCOR3,RORC,RPL14,RPL15,RPL18A,RPL27,RPL27A,RPL34,RPL35A,RPL5,RPL6,RPL7,RPL8,RPL9,RPS11,RPS13,RPS14,RPS2,RPS8,RRBP1,RXRA,SLC44A1,TARDBP,UBE2J1,YAE1D1,YLPM1,ZMYND8,ZNF184,ZNF512B,ZNF681,ZNF746,ZNF830 |
| GO:0051649 | establishment of localization in cell | 31 | 1616 | 0.0486 | ENSP00000215730,ENSP00000222247,ENSP00000244769,ENSP00000262584,ENSP00000270625,ENSP00000309334,ENSP00000310226,ENSP00000339795,ENSP00000341680,ENSP00000341885,ENSP00000346015,ENSP00000348965,ENSP00000349016,ENSP00000356042,ENSP00000357552,ENSP00000359345,ENSP00000361824,ENSP00000362146,ENSP00000365606,ENSP00000378163,ENSP00000379506,ENSP00000379888,ENSP00000384371,ENSP00000385958,ENSP00000400467,ENSP00000403172,ENSP00000419117,ENSP00000435777,ENSP00000452123,ENSP00000464813,ENSP00000479322 | ATXN1,BCL6,CDCA8,CLIP1,DTNBP1,DYNC1H1,EZR,GRIPAP1,KPNA5,PEX14,RAB1B,RPL14,RPL15,RPL18A,RPL27,RPL27A,RPL34,RPL35A,RPL5,RPL6,RPL7,RPL8,RPL9,RPS11,RPS13,RPS14,RPS2,RPS8,SNAP29,SPTAN1,SRSF5 |
| GO:1901576 | organic substance biosynthetic process | 70 | 4656 | 0.0492 | ENSP00000210060,ENSP00000211936,ENSP00000222247,ENSP00000223273,ENSP00000229239,ENSP00000233242,ENSP00000237853,ENSP00000240185,ENSP00000244769,ENSP00000250003,ENSP00000252602,ENSP00000253099,ENSP00000256578,ENSP00000259056,ENSP00000260600,ENSP00000262584,ENSP00000270625,ENSP00000273317,ENSP00000287820,ENSP00000291547,ENSP00000293860,ENSP00000296953,ENSP00000300291,ENSP00000309334,ENSP00000312631,ENSP00000316335,ENSP00000324463,ENSP00000327025,ENSP00000339795,ENSP00000341885,ENSP00000344260,ENSP00000345917,ENSP00000346015,ENSP00000353910,ENSP00000354518,ENSP00000356331,ENSP00000359345,ENSP00000363852,ENSP00000364145,ENSP00000364212,ENSP00000367038,ENSP00000368927,ENSP00000369519,ENSP00000376534,ENSP00000377836,ENSP00000378163,ENSP00000379506,ENSP00000379888,ENSP00000384000,ENSP00000384371,ENSP00000385958,ENSP00000389176,ENSP00000393795,ENSP00000394085,ENSP00000395007,ENSP00000400467,ENSP00000403172,ENSP00000413929,ENSP00000419117,ENSP00000419692,ENSP00000420095,ENSP00000425487,ENSP00000428340,ENSP00000430432,ENSP00000435777,ENSP00000440864,ENSP00000451261,ENSP00000464813,ENSP00000483552,ENSP00000484288 | ADCY3,AMPD2,ANGPT1,APOB,ATXN1,BAZ2B,BCL6,C1GALT1,CDA,CREBRF,DHPS,EIF1AX,ELL2,FARS2,FURIN,FUT8,GALNT15,GALNT5,GAPDH,GPC3,HDAC2,INPP4B,INSM1,JMJD6,LIG4,LIMD1,LYAR,MRPL34,MRPL4,MTAP,MYOD1,NR4A1,NR5A2,NUDT21,PFKFB1,PKNOX1,POLR3K,PPARG,RCOR3,RORC,RPL14,RPL15,RPL18A,RPL27,RPL27A,RPL34,RPL35A,RPL5,RPL6,RPL7,RPL8,RPL9,RPS11,RPS13,RPS14,RPS2,RPS8,RRBP1,RXRA,SLC44A1,TARDBP,UBE2J1,YAE1D1,YLPM1,ZMYND8,ZNF184,ZNF512B,ZNF681,ZNF746,ZNF830 |

Table S6_VCT_DEGs_Go_Cellular Component

| #term ID | term description | observed gene count | background gene count | false discovery rate | matching proteins in your network (IDs) | matching proteins in your network (labels) |
| --- | --- | --- | --- | --- | --- | --- |
| GO:0005829 | cytosol | 94 | 4958 | 9.98E-07 | ENSP00000044462,ENSP00000210060,ENSP00000215730,ENSP00000216455,ENSP00000216487,ENSP00000217652,ENSP00000220507,ENSP00000221233,ENSP00000222247,ENSP00000229239,ENSP00000233242,ENSP00000238616,ENSP00000244769,ENSP00000250003,ENSP00000253099,ENSP00000256578,ENSP00000258317,ENSP00000260600,ENSP00000261427,ENSP00000262319,ENSP00000262584,ENSP00000265564,ENSP00000270625,ENSP00000273317,ENSP00000274026,ENSP00000280154,ENSP00000285670,ENSP00000287820,ENSP00000293860,ENSP00000299084,ENSP00000301030,ENSP00000303427,ENSP00000306461,ENSP00000309334,ENSP00000310226,ENSP00000320176,ENSP00000324463,ENSP00000325074,ENSP00000337946,ENSP00000338788,ENSP00000339795,ENSP00000341680,ENSP00000341838,ENSP00000341885,ENSP00000346015,ENSP00000347742,ENSP00000348965,ENSP00000353910,ENSP00000356042,ENSP00000357552,ENSP00000357722,ENSP00000359345,ENSP00000361014,ENSP00000361433,ENSP00000361540,ENSP00000361824,ENSP00000362146,ENSP00000364145,ENSP00000364212,ENSP00000365606,ENSP00000366843,ENSP00000368927,ENSP00000369519,ENSP00000373715,ENSP00000373734,ENSP00000378163,ENSP00000378338,ENSP00000378974,ENSP00000379506,ENSP00000379888,ENSP00000380178,ENSP00000380929,ENSP00000384792,ENSP00000385080,ENSP00000385958,ENSP00000387122,ENSP00000391524,ENSP00000392660,ENSP00000394085,ENSP00000400467,ENSP00000401802,ENSP00000403172,ENSP00000418287,ENSP00000419117,ENSP00000424765,ENSP00000425487,ENSP00000429900,ENSP00000435777,ENSP00000440864,ENSP00000452123,ENSP00000464813,ENSP00000473631,ENSP00000479322,ENSP00000482472 | ADCY3,AMOTL2,AMPD2,ANKRD11,APOB,ARHGAP28,ASAP1,ATXN1,ATXN2,CCNA2,CDA,CDC20,CDCA8,CIAO1,CLEC16A,CLIP1,DCP2,DENND5B,DENND6B,DHPS,DNAJC3,DTNBP1,DYNC1H1,EIF1AX,EXOSC2,EXOSC5,EXOSC7,EZR,FAH,FUT8,GAPDH,GIT1,GRIPAP1,HCLS1,INPP4B,JMJD6,KPNA5,LIMD1,LPP,MAPK8,MKNK1,MRPL4,MTAP,MYL12A,MYOD1,NEK9,NPL,NR4A1,PACRG,PDCD4,PDS5A,PDZD3,PFKFB1,POLR3K,PPARG,PPP2R2A,PSMA3,PSMA4,PSMC6,RAB1B,RHOV,RIN3,RPL14,RPL15,RPL18A,RPL27,RPL27A,RPL34,RPL35A,RPL5,RPL6,RPL7,RPL8,RPL9,RPS11,RPS13,RPS14,RPS2,RPS8,S100A8,SAMSN1,SNAP29,SPRED1,SPRR1B,SPTAN1,SRSF5,TCP10,TELO2,TNNI3,UBE2G1,UBE2K,WDR62,YLPM1,ZC3H15 |
| GO:0005840 | ribosome | 15 | 201 | 3.27E-06 | ENSP00000222247,ENSP00000252602,ENSP00000253099,ENSP00000262584,ENSP00000270625,ENSP00000309334,ENSP00000341885,ENSP00000359859,ENSP00000367038,ENSP00000379888,ENSP00000385958,ENSP00000400467,ENSP00000403172,ENSP00000435777,ENSP00000464813 | MRPL34,MRPL4,MTG2,RPL15,RPL18A,RPL27,RPL6,RPL8,RPL9,RPS11,RPS13,RPS14,RPS2,RPS8,RRBP1 |
| GO:1990904 | ribonucleoprotein complex | 28 | 770 | 6.06E-06 | ENSP00000044462,ENSP00000222247,ENSP00000229239,ENSP00000236273,ENSP00000252602,ENSP00000253099,ENSP00000262584,ENSP00000270625,ENSP00000273317,ENSP00000309334,ENSP00000339795,ENSP00000341885,ENSP00000354518,ENSP00000359345,ENSP00000359859,ENSP00000366843,ENSP00000367038,ENSP00000373715,ENSP00000379888,ENSP00000384792,ENSP00000385958,ENSP00000394085,ENSP00000400467,ENSP00000403172,ENSP00000423067,ENSP00000435777,ENSP00000464813,ENSP00000484288 | ATXN2,DCP2,GAPDH,JMJD6,LIG4,LIMD1,MRPL34,MRPL4,MTG2,PSMA4,RPL15,RPL18A,RPL27,RPL5,RPL6,RPL7,RPL8,RPL9,RPS11,RPS13,RPS14,RPS2,RPS8,RRBP1,SYF2,WDR36,WDR62,ZNF830 |
| GO:0044445 | cytosolic part | 14 | 195 | 7.98E-06 | ENSP00000222247,ENSP00000253099,ENSP00000260600,ENSP00000262584,ENSP00000270625,ENSP00000341680,ENSP00000341885,ENSP00000364145,ENSP00000379888,ENSP00000385958,ENSP00000401802,ENSP00000403172,ENSP00000418287,ENSP00000435777 | ADCY3,CIAO1,DTNBP1,MRPL4,PFKFB1,PSMC6,RPL18A,RPL6,RPL8,RPS11,RPS13,RPS14,RPS2,RPS8 |
| GO:0005737 | cytoplasm | 158 | 11238 | 2.01E-05 | ENSP00000044462,ENSP00000210060,ENSP00000215730,ENSP00000216455,ENSP00000216487,ENSP00000217652,ENSP00000220507,ENSP00000221233,ENSP00000222247,ENSP00000229239,ENSP00000230859,ENSP00000233242,ENSP00000236192,ENSP00000238616,ENSP00000240185,ENSP00000244769,ENSP00000246554,ENSP00000250003,ENSP00000252602,ENSP00000253099,ENSP00000254442,ENSP00000256578,ENSP00000258317,ENSP00000259056,ENSP00000260600,ENSP00000261427,ENSP00000262319,ENSP00000262584,ENSP00000265564,ENSP00000267406,ENSP00000270625,ENSP00000273317,ENSP00000274026,ENSP00000280154,ENSP00000285670,ENSP00000286234,ENSP00000287490,ENSP00000287820,ENSP00000290219,ENSP00000291547,ENSP00000293860,ENSP00000294304,ENSP00000296953,ENSP00000299084,ENSP00000300289,ENSP00000300291,ENSP00000301030,ENSP00000303427,ENSP00000304642,ENSP00000305976,ENSP00000306185,ENSP00000306461,ENSP00000309334,ENSP00000310226,ENSP00000310557,ENSP00000316335,ENSP00000320176,ENSP00000323074,ENSP00000323696,ENSP00000324463,ENSP00000325074,ENSP00000327716,ENSP00000330572,ENSP00000337946,ENSP00000338788,ENSP00000339795,ENSP00000341680,ENSP00000341838,ENSP00000341885,ENSP00000344260,ENSP00000345917,ENSP00000346015,ENSP00000346151,ENSP00000346931,ENSP00000347742,ENSP00000348308,ENSP00000348965,ENSP00000349016,ENSP00000349456,ENSP00000350348,ENSP00000353910,ENSP00000356042,ENSP00000356331,ENSP00000357552,ENSP00000357722,ENSP00000358795,ENSP00000359345,ENSP00000359621,ENSP00000359859,ENSP00000360214,ENSP00000361014,ENSP00000361433,ENSP00000361540,ENSP00000361824,ENSP00000362146,ENSP00000363298,ENSP00000363852,ENSP00000364145,ENSP00000364212,ENSP00000365477,ENSP00000365606,ENSP00000366843,ENSP00000367038,ENSP00000368927,ENSP00000369519,ENSP00000369860,ENSP00000371018,ENSP00000371472,ENSP00000373715,ENSP00000373734,ENSP00000377836,ENSP00000378163,ENSP00000378338,ENSP00000378340,ENSP00000378974,ENSP00000379506,ENSP00000379888,ENSP00000380178,ENSP00000380185,ENSP00000380929,ENSP00000384371,ENSP00000384792,ENSP00000385080,ENSP00000385958,ENSP00000386184,ENSP00000387122,ENSP00000389176,ENSP00000391524,ENSP00000392660,ENSP00000394085,ENSP00000395007,ENSP00000400467,ENSP00000401802,ENSP00000403172,ENSP00000411528,ENSP00000414398,ENSP00000418287,ENSP00000419117,ENSP00000420095,ENSP00000424765,ENSP00000425487,ENSP00000429900,ENSP00000430432,ENSP00000435777,ENSP00000440864,ENSP00000443926,ENSP00000451261,ENSP00000452123,ENSP00000464813,ENSP00000469880,ENSP00000473631,ENSP00000475216,ENSP00000479322,ENSP00000479542,ENSP00000480571,ENSP00000482472,ENSP00000483552,ENSP00000484288 | ADCY3,AMOTL2,AMPD2,ANKRD11,ANTXR2,APOB,ARHGAP28,ASAP1,ATXN1,ATXN2,BCL6,BSPRY,C1GALT1,C6orf120,CBLN3,CCDC93,CCNA2,CDA,CDC20,CDCA8,CELF2,CIAO1,CLCC1,CLEC16A,CLIP1,COL4A6,COX6A2,COX6B1,CREBRF,CRTAP,CYP3A7,DCP2,DENND5B,DENND6B,DEPTOR,DHPS,DNAJB11,DNAJC3,DTNBP1,DYNC1H1,EIF1AX,EXOSC2,EXOSC5,EXOSC7,EZR,FAH,FAM134B,FARS2,FURIN,FUT8,GALNT15,GALNT5,GAPDH,GIT1,GJC1,GPC3,GRIPAP1,GRM7,HCLS1,HDAC2,IFNGR2,INPP4B,JMJD6,KCNE3,KIZ,KPNA5,KPRP,LIG4,LIMD1,LPP,LRP5,LYAR,MAGI2,MAPK8,MAPK8IP2,MCOLN3,MKNK1,MMP23B,MOSPD2,MRPL34,MRPL4,MTAP,MTG2,MYL12A,MYOD1,NEK9,NEURL1,NIN,NPL,NR4A1,NR5A2,NUDT21,OPALIN,PACRG,PAPD7,PDCD4,PDIA3,PDS5A,PDZD3,PEX14,PFKFB1,PKNOX1,PLEKHG6,POLR3K,PPARG,PPP2R2A,PSMA3,PSMA4,PSMC6,RAB1B,RAI1,RHOV,RIN3,RPL14,RPL15,RPL18A,RPL27,RPL27A,RPL34,RPL35A,RPL5,RPL6,RPL7,RPL8,RPL9,RPS11,RPS13,RPS14,RPS2,RPS8,RRBP1,S100A8,SAMSN1,SIK2,SIRPB1,SLC44A1,SNAP29,SPRED1,SPRR1B,SPTAN1,SRSF5,TARDBP,TCP10,TELO2,TNNI3,UBE2G1,UBE2J1,UBE2K,VAMP4,VRK3,WDR62,WDR7,WRB,YLPM1,ZBED8,ZC3H15,ZMYND8,ZNF746 |
| GO:0044444 | cytoplasmic part | 139 | 9377 | 2.01E-05 | ENSP00000044462,ENSP00000210060,ENSP00000215730,ENSP00000216455,ENSP00000216487,ENSP00000217652,ENSP00000220507,ENSP00000221233,ENSP00000222247,ENSP00000229239,ENSP00000230859,ENSP00000233242,ENSP00000236192,ENSP00000238616,ENSP00000244769,ENSP00000246554,ENSP00000250003,ENSP00000252602,ENSP00000253099,ENSP00000254442,ENSP00000256578,ENSP00000258317,ENSP00000259056,ENSP00000260600,ENSP00000261427,ENSP00000262319,ENSP00000262584,ENSP00000265564,ENSP00000267406,ENSP00000270625,ENSP00000273317,ENSP00000274026,ENSP00000280154,ENSP00000285670,ENSP00000287490,ENSP00000287820,ENSP00000290219,ENSP00000293860,ENSP00000294304,ENSP00000299084,ENSP00000300289,ENSP00000301030,ENSP00000303427,ENSP00000304642,ENSP00000306185,ENSP00000306461,ENSP00000309334,ENSP00000310226,ENSP00000316335,ENSP00000320176,ENSP00000323074,ENSP00000323696,ENSP00000324463,ENSP00000325074,ENSP00000327716,ENSP00000337946,ENSP00000338788,ENSP00000339795,ENSP00000341680,ENSP00000341838,ENSP00000341885,ENSP00000344260,ENSP00000346015,ENSP00000346151,ENSP00000346931,ENSP00000347742,ENSP00000348308,ENSP00000348965,ENSP00000349016,ENSP00000349456,ENSP00000350348,ENSP00000353910,ENSP00000356042,ENSP00000357552,ENSP00000357722,ENSP00000358795,ENSP00000359345,ENSP00000359621,ENSP00000359859,ENSP00000360214,ENSP00000361014,ENSP00000361433,ENSP00000361540,ENSP00000361824,ENSP00000362146,ENSP00000363298,ENSP00000363852,ENSP00000364145,ENSP00000364212,ENSP00000365477,ENSP00000365606,ENSP00000366843,ENSP00000367038,ENSP00000368927,ENSP00000369519,ENSP00000369860,ENSP00000371018,ENSP00000371472,ENSP00000373715,ENSP00000373734,ENSP00000377836,ENSP00000378163,ENSP00000378338,ENSP00000378340,ENSP00000378974,ENSP00000379506,ENSP00000379888,ENSP00000380178,ENSP00000380929,ENSP00000384371,ENSP00000384792,ENSP00000385080,ENSP00000385958,ENSP00000387122,ENSP00000389176,ENSP00000391524,ENSP00000392660,ENSP00000394085,ENSP00000400467,ENSP00000401802,ENSP00000403172,ENSP00000411528,ENSP00000414398,ENSP00000418287,ENSP00000419117,ENSP00000424765,ENSP00000425487,ENSP00000429900,ENSP00000435777,ENSP00000440864,ENSP00000451261,ENSP00000452123,ENSP00000464813,ENSP00000473631,ENSP00000479322,ENSP00000480571,ENSP00000482472,ENSP00000483552,ENSP00000484288 | ADCY3,AMOTL2,AMPD2,ANKRD11,ANTXR2,APOB,ARHGAP28,ASAP1,ATXN1,ATXN2,BCL6,BSPRY,C1GALT1,C6orf120,CBLN3,CCDC93,CCNA2,CDA,CDC20,CDCA8,CIAO1,CLCC1,CLEC16A,CLIP1,COL4A6,COX6A2,COX6B1,CRTAP,CYP3A7,DCP2,DENND5B,DENND6B,DHPS,DNAJB11,DNAJC3,DTNBP1,DYNC1H1,EIF1AX,EXOSC2,EXOSC5,EXOSC7,EZR,FAH,FAM134B,FARS2,FURIN,FUT8,GALNT15,GALNT5,GAPDH,GIT1,GJC1,GPC3,GRIPAP1,GRM7,HCLS1,IFNGR2,INPP4B,JMJD6,KPNA5,LIG4,LIMD1,LPP,LRP5,MAGI2,MAPK8,MCOLN3,MKNK1,MMP23B,MOSPD2,MRPL34,MRPL4,MTAP,MTG2,MYL12A,MYOD1,NEK9,NEURL1,NIN,NPL,NR4A1,OPALIN,PACRG,PAPD7,PDCD4,PDIA3,PDS5A,PDZD3,PEX14,PFKFB1,POLR3K,PPARG,PPP2R2A,PSMA3,PSMA4,PSMC6,RAB1B,RAI1,RHOV,RIN3,RPL14,RPL15,RPL18A,RPL27,RPL27A,RPL34,RPL35A,RPL5,RPL6,RPL7,RPL8,RPL9,RPS11,RPS13,RPS14,RPS2,RPS8,RRBP1,S100A8,SAMSN1,SIRPB1,SLC44A1,SNAP29,SPRED1,SPRR1B,SPTAN1,SRSF5,TCP10,TELO2,TNNI3,UBE2G1,UBE2J1,UBE2K,VAMP4,WDR62,WDR7,WRB,YLPM1,ZC3H15 |
| GO:0022626 | cytosolic ribosome | 9 | 78 | 2.93E-05 | ENSP00000222247,ENSP00000253099,ENSP00000262584,ENSP00000270625,ENSP00000341885,ENSP00000379888,ENSP00000385958,ENSP00000403172,ENSP00000435777 | MRPL4,RPL18A,RPL6,RPL8,RPS11,RPS13,RPS14,RPS2,RPS8 |
| GO:0032991 | protein-containing complex | 82 | 4792 | 0.00025 | ENSP00000044462,ENSP00000215730,ENSP00000216455,ENSP00000217652,ENSP00000221233,ENSP00000222247,ENSP00000229239,ENSP00000233242,ENSP00000236192,ENSP00000236273,ENSP00000237853,ENSP00000250003,ENSP00000252602,ENSP00000253099,ENSP00000260600,ENSP00000262319,ENSP00000262584,ENSP00000265074,ENSP00000265564,ENSP00000270625,ENSP00000273317,ENSP00000274026,ENSP00000287490,ENSP00000287820,ENSP00000291547,ENSP00000293860,ENSP00000294304,ENSP00000300291,ENSP00000309334,ENSP00000310557,ENSP00000312631,ENSP00000320176,ENSP00000323696,ENSP00000325074,ENSP00000330572,ENSP00000334448,ENSP00000339795,ENSP00000341680,ENSP00000341838,ENSP00000341885,ENSP00000346151,ENSP00000347742,ENSP00000348965,ENSP00000349016,ENSP00000349456,ENSP00000350348,ENSP00000354518,ENSP00000356042,ENSP00000356331,ENSP00000357552,ENSP00000359345,ENSP00000359859,ENSP00000361433,ENSP00000361540,ENSP00000362146,ENSP00000364145,ENSP00000366843,ENSP00000367038,ENSP00000371472,ENSP00000373715,ENSP00000378340,ENSP00000379888,ENSP00000384792,ENSP00000385958,ENSP00000394085,ENSP00000400467,ENSP00000401802,ENSP00000403172,ENSP00000409544,ENSP00000411528,ENSP00000413929,ENSP00000418287,ENSP00000419692,ENSP00000423067,ENSP00000429900,ENSP00000430432,ENSP00000435777,ENSP00000464813,ENSP00000473631,ENSP00000475814,ENSP00000479322,ENSP00000484288 | ADCY3,APOB,ASAP1,ATXN2,CCNA2,CDC20,CDCA8,CIAO1,CLCC1,CLDN4,CLIP1,COL4A6,COX6A2,CRTAP,DCP2,DNAJC3,DTNBP1,DYNC1H1,ELL2,EXOSC2,EXOSC5,EXOSC7,EZR,GAPDH,GJC1,GNG2,GRM7,HCLS1,HDAC2,HIST2H2AA3,INSM1,JMJD6,KCNE3,KPNA5,LIG4,LIMD1,LRP5,MAGI2,MAPK8IP2,MRPL34,MRPL4,MTG2,MYL12A,MYOD1,NIN,NPR3,NR5A2,NUDT21,PDZD3,PEX14,PFKFB1,PKNOX1,POLR3K,PPARG,PPP2R2A,PSMA3,PSMA4,PSMC6,RCOR3,RPL15,RPL18A,RPL27,RPL5,RPL6,RPL7,RPL8,RPL9,RPS11,RPS13,RPS14,RPS2,RPS8,RRBP1,RXRA,SNAP29,SYF2,TELO2,TNNI3,VAMP4,WDR36,WDR62,ZNF830 |
| GO:0044391 | ribosomal subunit | 10 | 152 | 0.00059 | ENSP00000222247,ENSP00000252602,ENSP00000253099,ENSP00000262584,ENSP00000270625,ENSP00000341885,ENSP00000379888,ENSP00000385958,ENSP00000403172,ENSP00000435777 | MRPL34,MRPL4,RPL18A,RPL6,RPL8,RPS11,RPS13,RPS14,RPS2,RPS8 |
| GO:0005622 | intracellular | 181 | 14286 | 0.00072 | ENSP00000044462,ENSP00000210060,ENSP00000211936,ENSP00000215730,ENSP00000216455,ENSP00000216487,ENSP00000217652,ENSP00000220507,ENSP00000221233,ENSP00000222247,ENSP00000229239,ENSP00000230859,ENSP00000233242,ENSP00000236192,ENSP00000236273,ENSP00000237853,ENSP00000238616,ENSP00000240185,ENSP00000244769,ENSP00000246554,ENSP00000250003,ENSP00000252602,ENSP00000253099,ENSP00000254442,ENSP00000256578,ENSP00000258317,ENSP00000259056,ENSP00000260600,ENSP00000261427,ENSP00000262319,ENSP00000262584,ENSP00000263773,ENSP00000265074,ENSP00000265564,ENSP00000267406,ENSP00000270625,ENSP00000273317,ENSP00000274026,ENSP00000274306,ENSP00000276431,ENSP00000280154,ENSP00000285670,ENSP00000286234,ENSP00000287490,ENSP00000287820,ENSP00000290219,ENSP00000291547,ENSP00000293860,ENSP00000294304,ENSP00000296953,ENSP00000298307,ENSP00000299084,ENSP00000300289,ENSP00000300291,ENSP00000301030,ENSP00000303427,ENSP00000304642,ENSP00000305976,ENSP00000306185,ENSP00000306461,ENSP00000306822,ENSP00000309334,ENSP00000310226,ENSP00000310557,ENSP00000312631,ENSP00000316335,ENSP00000320176,ENSP00000323074,ENSP00000323696,ENSP00000324463,ENSP00000325074,ENSP00000327025,ENSP00000327716,ENSP00000330572,ENSP00000334448,ENSP00000337946,ENSP00000338788,ENSP00000339795,ENSP00000341680,ENSP00000341838,ENSP00000341885,ENSP00000344260,ENSP00000345917,ENSP00000346015,ENSP00000346151,ENSP00000346931,ENSP00000347742,ENSP00000348308,ENSP00000348965,ENSP00000349016,ENSP00000349456,ENSP00000350348,ENSP00000353910,ENSP00000354518,ENSP00000356042,ENSP00000356331,ENSP00000357552,ENSP00000357722,ENSP00000358795,ENSP00000359345,ENSP00000359621,ENSP00000359859,ENSP00000360214,ENSP00000361014,ENSP00000361433,ENSP00000361540,ENSP00000361824,ENSP00000362146,ENSP00000363298,ENSP00000363852,ENSP00000364145,ENSP00000364212,ENSP00000365477,ENSP00000365606,ENSP00000366843,ENSP00000367038,ENSP00000368927,ENSP00000369519,ENSP00000369860,ENSP00000371018,ENSP00000371472,ENSP00000373715,ENSP00000373734,ENSP00000376534,ENSP00000377836,ENSP00000378163,ENSP00000378338,ENSP00000378340,ENSP00000378974,ENSP00000379506,ENSP00000379888,ENSP00000380178,ENSP00000380185,ENSP00000380929,ENSP00000384000,ENSP00000384371,ENSP00000384792,ENSP00000385080,ENSP00000385958,ENSP00000386184,ENSP00000387122,ENSP00000389176,ENSP00000391524,ENSP00000392660,ENSP00000393795,ENSP00000394085,ENSP00000395007,ENSP00000400467,ENSP00000401802,ENSP00000403172,ENSP00000411528,ENSP00000413929,ENSP00000414398,ENSP00000418287,ENSP00000418563,ENSP00000419117,ENSP00000419692,ENSP00000420095,ENSP00000420211,ENSP00000423067,ENSP00000424765,ENSP00000425487,ENSP00000428340,ENSP00000429900,ENSP00000430432,ENSP00000435777,ENSP00000440864,ENSP00000443926,ENSP00000451261,ENSP00000452123,ENSP00000464813,ENSP00000469880,ENSP00000473631,ENSP00000475216,ENSP00000475814,ENSP00000479322,ENSP00000479542,ENSP00000480571,ENSP00000482472,ENSP00000483552,ENSP00000484288 | ADCY3,AMOTL2,AMPD2,ANGPT1,ANKRD11,ANTXR2,APOB,ARHGAP28,ASAP1,ATXN1,ATXN2,AVEN,BAZ2B,BCL6,BSPRY,C1GALT1,C6orf120,CBLN3,CCDC93,CCNA2,CDA,CDC20,CDCA8,CELF2,CIAO1,CLCC1,CLEC16A,CLIP1,COL4A6,COX6A2,COX6B1,CREBRF,CRTAP,CYP3A7,DCP2,DENND5B,DENND6B,DEPTOR,DHPS,DNAJB11,DNAJC3,DTNBP1,DYNC1H1,EIF1AX,ELL2,EXOSC2,EXOSC5,EXOSC7,EZR,FAH,FAM134B,FARS2,FNBP4,FURIN,FUT8,GALNT15,GALNT5,GAPDH,GIT1,GJC1,GNG2,GPC3,GRIPAP1,GRM7,GZMA,HCLS1,HDAC2,HIST2H2AA3,IFNGR2,INPP4B,INSM1,JMJD6,KCNE3,KIZ,KLHDC2,KPNA5,KPRP,LIG4,LIMD1,LPP,LRP5,LYAR,MAGI2,MAPK8,MAPK8IP2,MCOLN3,MKNK1,MMP23B,MOSPD2,MRPL34,MRPL4,MTAP,MTG2,MYL12A,MYOD1,NEK9,NEURL1,NIN,NPL,NPR3,NR4A1,NR5A2,NUDT21,OPALIN,PACRG,PAPD7,PDCD4,PDIA3,PDS5A,PDZD3,PEX14,PFKFB1,PKNOX1,PLEKHG6,POLR3K,PPARG,PPP2R2A,PSMA3,PSMA4,PSMC6,RAB1B,RAI1,RCOR3,RGL2,RHOV,RIN3,RORC,RPL14,RPL15,RPL18A,RPL27,RPL27A,RPL34,RPL35A,RPL5,RPL6,RPL7,RPL8,RPL9,RPS11,RPS13,RPS14,RPS2,RPS8,RRBP1,RXRA,S100A8,SAMSN1,SIK2,SIRPB1,SLC44A1,SNAP29,SPRED1,SPRR1B,SPTAN1,SRSF5,SYF2,TARDBP,TCP10,TELO2,TNFRSF10B,TNNI3,U2SURP,UBE2G1,UBE2J1,UBE2K,VAMP4,VRK3,WDR36,WDR62,WDR7,WRB,YLPM1,ZBED8,ZC3H15,ZMYND8,ZNF184,ZNF512B,ZNF681,ZNF746,ZNF830 |
| GO:0044424 | intracellular part | 176 | 13996 | 0.0037 | ENSP00000044462,ENSP00000210060,ENSP00000211936,ENSP00000215730,ENSP00000216455,ENSP00000216487,ENSP00000217652,ENSP00000220507,ENSP00000221233,ENSP00000222247,ENSP00000229239,ENSP00000230859,ENSP00000233242,ENSP00000236192,ENSP00000236273,ENSP00000237853,ENSP00000238616,ENSP00000240185,ENSP00000244769,ENSP00000246554,ENSP00000250003,ENSP00000252602,ENSP00000253099,ENSP00000254442,ENSP00000256578,ENSP00000258317,ENSP00000259056,ENSP00000260600,ENSP00000261427,ENSP00000262319,ENSP00000262584,ENSP00000263773,ENSP00000265564,ENSP00000267406,ENSP00000270625,ENSP00000273317,ENSP00000274026,ENSP00000274306,ENSP00000280154,ENSP00000285670,ENSP00000286234,ENSP00000287490,ENSP00000287820,ENSP00000290219,ENSP00000291547,ENSP00000293860,ENSP00000294304,ENSP00000296953,ENSP00000298307,ENSP00000299084,ENSP00000300289,ENSP00000300291,ENSP00000301030,ENSP00000303427,ENSP00000304642,ENSP00000305976,ENSP00000306185,ENSP00000306461,ENSP00000309334,ENSP00000310226,ENSP00000310557,ENSP00000312631,ENSP00000316335,ENSP00000320176,ENSP00000323074,ENSP00000323696,ENSP00000324463,ENSP00000325074,ENSP00000327025,ENSP00000327716,ENSP00000330572,ENSP00000334448,ENSP00000337946,ENSP00000338788,ENSP00000339795,ENSP00000341680,ENSP00000341838,ENSP00000341885,ENSP00000344260,ENSP00000345917,ENSP00000346015,ENSP00000346151,ENSP00000346931,ENSP00000347742,ENSP00000348308,ENSP00000348965,ENSP00000349016,ENSP00000349456,ENSP00000350348,ENSP00000353910,ENSP00000354518,ENSP00000356042,ENSP00000356331,ENSP00000357552,ENSP00000357722,ENSP00000358795,ENSP00000359345,ENSP00000359621,ENSP00000359859,ENSP00000360214,ENSP00000361014,ENSP00000361433,ENSP00000361540,ENSP00000361824,ENSP00000362146,ENSP00000363298,ENSP00000363852,ENSP00000364145,ENSP00000364212,ENSP00000365477,ENSP00000365606,ENSP00000366843,ENSP00000367038,ENSP00000368927,ENSP00000369519,ENSP00000369860,ENSP00000371018,ENSP00000371472,ENSP00000373715,ENSP00000373734,ENSP00000376534,ENSP00000377836,ENSP00000378163,ENSP00000378338,ENSP00000378340,ENSP00000378974,ENSP00000379506,ENSP00000379888,ENSP00000380178,ENSP00000380185,ENSP00000380929,ENSP00000384000,ENSP00000384371,ENSP00000384792,ENSP00000385080,ENSP00000385958,ENSP00000386184,ENSP00000387122,ENSP00000389176,ENSP00000391524,ENSP00000392660,ENSP00000393795,ENSP00000394085,ENSP00000395007,ENSP00000400467,ENSP00000401802,ENSP00000403172,ENSP00000411528,ENSP00000413929,ENSP00000414398,ENSP00000418287,ENSP00000418563,ENSP00000419117,ENSP00000419692,ENSP00000420095,ENSP00000423067,ENSP00000424765,ENSP00000425487,ENSP00000429900,ENSP00000430432,ENSP00000435777,ENSP00000440864,ENSP00000443926,ENSP00000451261,ENSP00000452123,ENSP00000464813,ENSP00000469880,ENSP00000473631,ENSP00000475216,ENSP00000475814,ENSP00000479322,ENSP00000479542,ENSP00000480571,ENSP00000482472,ENSP00000483552,ENSP00000484288 | ADCY3,AMOTL2,AMPD2,ANKRD11,ANTXR2,APOB,ARHGAP28,ASAP1,ATXN1,ATXN2,BAZ2B,BCL6,BSPRY,C1GALT1,C6orf120,CBLN3,CCDC93,CCNA2,CDA,CDC20,CDCA8,CELF2,CIAO1,CLCC1,CLEC16A,CLIP1,COL4A6,COX6A2,COX6B1,CREBRF,CRTAP,CYP3A7,DCP2,DENND5B,DENND6B,DEPTOR,DHPS,DNAJB11,DNAJC3,DTNBP1,DYNC1H1,EIF1AX,ELL2,EXOSC2,EXOSC5,EXOSC7,EZR,FAH,FAM134B,FARS2,FNBP4,FURIN,FUT8,GALNT15,GALNT5,GAPDH,GIT1,GJC1,GNG2,GPC3,GRIPAP1,GRM7,GZMA,HCLS1,HDAC2,HIST2H2AA3,IFNGR2,INPP4B,INSM1,JMJD6,KCNE3,KIZ,KLHDC2,KPNA5,KPRP,LIG4,LIMD1,LPP,LRP5,LYAR,MAGI2,MAPK8,MAPK8IP2,MCOLN3,MKNK1,MMP23B,MOSPD2,MRPL34,MRPL4,MTAP,MTG2,MYL12A,MYOD1,NEK9,NEURL1,NIN,NPL,NR4A1,NR5A2,NUDT21,OPALIN,PACRG,PAPD7,PDCD4,PDIA3,PDS5A,PDZD3,PEX14,PFKFB1,PKNOX1,PLEKHG6,POLR3K,PPARG,PPP2R2A,PSMA3,PSMA4,PSMC6,RAB1B,RAI1,RCOR3,RHOV,RIN3,RORC,RPL14,RPL15,RPL18A,RPL27,RPL27A,RPL34,RPL35A,RPL5,RPL6,RPL7,RPL8,RPL9,RPS11,RPS13,RPS14,RPS2,RPS8,RRBP1,RXRA,S100A8,SAMSN1,SIK2,SIRPB1,SLC44A1,SNAP29,SPRED1,SPRR1B,SPTAN1,SRSF5,SYF2,TARDBP,TCP10,TELO2,TNNI3,U2SURP,UBE2G1,UBE2J1,UBE2K,VAMP4,VRK3,WDR36,WDR62,WDR7,WRB,YLPM1,ZBED8,ZC3H15,ZMYND8,ZNF184,ZNF512B,ZNF681,ZNF746,ZNF830 |
| GO:0022627 | cytosolic small ribosomal subunit | 5 | 38 | 0.0041 | ENSP00000270625,ENSP00000341885,ENSP00000379888,ENSP00000385958,ENSP00000435777 | RPS11,RPS13,RPS14,RPS2,RPS8 |
| GO:0070013 | intracellular organelle lumen | 80 | 5162 | 0.0084 | ENSP00000044462,ENSP00000215730,ENSP00000216455,ENSP00000221233,ENSP00000230859,ENSP00000233242,ENSP00000236273,ENSP00000237853,ENSP00000240185,ENSP00000244769,ENSP00000246554,ENSP00000250003,ENSP00000252602,ENSP00000253099,ENSP00000262319,ENSP00000263773,ENSP00000265564,ENSP00000270625,ENSP00000274026,ENSP00000287820,ENSP00000293860,ENSP00000296953,ENSP00000298307,ENSP00000300289,ENSP00000300291,ENSP00000301030,ENSP00000303427,ENSP00000304642,ENSP00000312631,ENSP00000316335,ENSP00000323074,ENSP00000323696,ENSP00000324463,ENSP00000325074,ENSP00000327025,ENSP00000338788,ENSP00000341885,ENSP00000345917,ENSP00000346931,ENSP00000348965,ENSP00000349016,ENSP00000354518,ENSP00000356042,ENSP00000356331,ENSP00000357552,ENSP00000357722,ENSP00000359345,ENSP00000359859,ENSP00000361014,ENSP00000361433,ENSP00000361540,ENSP00000361824,ENSP00000362146,ENSP00000363852,ENSP00000364212,ENSP00000365606,ENSP00000371472,ENSP00000373715,ENSP00000377836,ENSP00000378340,ENSP00000378974,ENSP00000379888,ENSP00000384371,ENSP00000385958,ENSP00000386184,ENSP00000394085,ENSP00000401802,ENSP00000414398,ENSP00000418563,ENSP00000419692,ENSP00000423067,ENSP00000430432,ENSP00000435777,ENSP00000440864,ENSP00000452123,ENSP00000469880,ENSP00000473631,ENSP00000475814,ENSP00000483552,ENSP00000484288 | ANKRD11,APOB,ATXN1,BCL6,C6orf120,CCNA2,CDA,CDC20,CDCA8,COL4A6,COX6B1,CREBRF,CRTAP,DCP2,DNAJB11,DNAJC3,DYNC1H1,ELL2,EXOSC2,EXOSC5,EXOSC7,EZR,FAM134B,FARS2,FNBP4,FURIN,GPC3,GRIPAP1,HDAC2,HIST2H2AA3,INSM1,JMJD6,KLHDC2,KPNA5,LIG4,LYAR,MAPK8,MKNK1,MRPL34,MRPL4,MTG2,MYOD1,NIN,NR4A1,NR5A2,NUDT21,PAPD7,PDIA3,PDS5A,PEX14,POLR3K,PPARG,PPP2R2A,PSMA3,PSMA4,PSMC6,RAI1,RORC,RPL5,RPS11,RPS13,RPS14,RPS2,RPS8,RXRA,S100A8,SLC44A1,SNAP29,SPTAN1,SRSF5,SYF2,TARDBP,TELO2,U2SURP,VRK3,WDR36,YLPM1,ZBED8,ZC3H15,ZNF830 |
| GO:0000176 | nuclear exosome (RNase complex) | 3 | 12 | 0.0164 | ENSP00000221233,ENSP00000265564,ENSP00000361433 | EXOSC2,EXOSC5,EXOSC7 |
| GO:0000177 | cytoplasmic exosome (RNase complex) | 3 | 13 | 0.0188 | ENSP00000221233,ENSP00000265564,ENSP00000361433 | EXOSC2,EXOSC5,EXOSC7 |
| GO:0043227 | membrane-bounded organelle | 145 | 11244 | 0.0217 | ENSP00000044462,ENSP00000211936,ENSP00000215730,ENSP00000216455,ENSP00000216487,ENSP00000220507,ENSP00000221233,ENSP00000229239,ENSP00000230859,ENSP00000233242,ENSP00000236192,ENSP00000236273,ENSP00000237853,ENSP00000238616,ENSP00000240185,ENSP00000244769,ENSP00000246554,ENSP00000250003,ENSP00000252602,ENSP00000253099,ENSP00000254442,ENSP00000259056,ENSP00000260600,ENSP00000261427,ENSP00000262319,ENSP00000263773,ENSP00000265564,ENSP00000265715,ENSP00000267406,ENSP00000270625,ENSP00000273317,ENSP00000274026,ENSP00000274306,ENSP00000280154,ENSP00000285670,ENSP00000287490,ENSP00000287820,ENSP00000290219,ENSP00000291547,ENSP00000293860,ENSP00000294304,ENSP00000296953,ENSP00000298307,ENSP00000299084,ENSP00000300289,ENSP00000300291,ENSP00000301030,ENSP00000303427,ENSP00000304642,ENSP00000305976,ENSP00000306185,ENSP00000309334,ENSP00000310226,ENSP00000310557,ENSP00000312631,ENSP00000316335,ENSP00000320176,ENSP00000323074,ENSP00000323696,ENSP00000324463,ENSP00000325074,ENSP00000327025,ENSP00000327716,ENSP00000337946,ENSP00000338788,ENSP00000341680,ENSP00000341885,ENSP00000344260,ENSP00000345917,ENSP00000346015,ENSP00000346151,ENSP00000346931,ENSP00000348308,ENSP00000348965,ENSP00000349016,ENSP00000349456,ENSP00000353910,ENSP00000354518,ENSP00000356042,ENSP00000356331,ENSP00000357552,ENSP00000357722,ENSP00000359345,ENSP00000359621,ENSP00000359859,ENSP00000360214,ENSP00000361014,ENSP00000361433,ENSP00000361540,ENSP00000361824,ENSP00000362146,ENSP00000363852,ENSP00000364212,ENSP00000365477,ENSP00000365606,ENSP00000366843,ENSP00000367038,ENSP00000369519,ENSP00000369860,ENSP00000371018,ENSP00000371472,ENSP00000373715,ENSP00000376534,ENSP00000377836,ENSP00000378163,ENSP00000378340,ENSP00000378974,ENSP00000379888,ENSP00000384000,ENSP00000384371,ENSP00000384792,ENSP00000385958,ENSP00000386184,ENSP00000387122,ENSP00000389176,ENSP00000391524,ENSP00000393795,ENSP00000394085,ENSP00000395007,ENSP00000401802,ENSP00000403172,ENSP00000411528,ENSP00000413929,ENSP00000414398,ENSP00000418563,ENSP00000419692,ENSP00000420095,ENSP00000423067,ENSP00000424765,ENSP00000430432,ENSP00000435777,ENSP00000440864,ENSP00000443926,ENSP00000451261,ENSP00000452123,ENSP00000464813,ENSP00000469880,ENSP00000473631,ENSP00000475814,ENSP00000479322,ENSP00000480571,ENSP00000480715,ENSP00000482472,ENSP00000483552,ENSP00000484288 | ADCY3,AMOTL2,ANKRD11,ANTXR2,APOB,ATXN1,ATXN2,BAZ2B,BCL6,C1GALT1,C6orf120,CBLN3,CCDC93,CCNA2,CDA,CDC20,CDCA8,CELF2,CLCC1,CLEC16A,CLIP1,COL4A6,COX6A2,COX6B1,CREBRF,CRTAP,CYP3A7,DCP2,DENND6B,DNAJB11,DNAJC3,DTNBP1,DYNC1H1,ELL2,EXOSC2,EXOSC5,EXOSC7,EZR,FAM134B,FARS2,FNBP4,FURIN,FUT8,GALNT15,GALNT5,GAPDH,GJC1,GPC3,GRIPAP1,GZMA,HCLS1,HDAC2,HIST2H2AA3,IFNGR2,INSM1,IQCE,JMJD6,KCNE3,KLHDC2,KPNA5,LIG4,LIMD1,LPP,LRP5,LYAR,MAGI2,MAPK8,MCOLN3,MKNK1,MMP23B,MOSPD2,MRPL34,MRPL4,MTAP,MTG2,MYOD1,NEK9,NIN,NR4A1,NR5A2,NUDT21,OPALIN,PACRG,PAPD7,PDCD4,PDIA3,PDS5A,PEX14,PKNOX1,POLR3K,PPARG,PPP2R2A,PSMA3,PSMA4,PSMC6,RAB1B,RAI1,RCOR3,RHOV,RIN3,RORC,RPL15,RPL27,RPL27A,RPL34,RPL5,RPL6,RPS11,RPS13,RPS14,RPS2,RPS8,RRBP1,RXRA,S100A8,SAMSN1,SIK2,SIRPB1,SLC26A4,SLC44A1,SNAP29,SPRED1,SPTAN1,SRSF5,SYF2,TARDBP,TELO2,U2SURP,UBE2J1,UBE2K,VAMP4,VRK3,WDR36,WDR62,WDR7,WRB,YLPM1,ZBED8,ZC3H15,ZMYND8,ZNF184,ZNF512B,ZNF681,ZNF746,ZNF830 |
| GO:0043226 | organelle | 156 | 12432 | 0.0335 | ENSP00000044462,ENSP00000211936,ENSP00000215730,ENSP00000216455,ENSP00000216487,ENSP00000217652,ENSP00000220507,ENSP00000221233,ENSP00000222247,ENSP00000229239,ENSP00000230859,ENSP00000233242,ENSP00000236192,ENSP00000236273,ENSP00000237853,ENSP00000238616,ENSP00000240185,ENSP00000244769,ENSP00000246554,ENSP00000250003,ENSP00000252602,ENSP00000253099,ENSP00000254442,ENSP00000259056,ENSP00000260600,ENSP00000261427,ENSP00000262319,ENSP00000262584,ENSP00000263773,ENSP00000265564,ENSP00000265715,ENSP00000267406,ENSP00000270625,ENSP00000273317,ENSP00000274026,ENSP00000274306,ENSP00000280154,ENSP00000285670,ENSP00000287490,ENSP00000287820,ENSP00000290219,ENSP00000291547,ENSP00000293860,ENSP00000294304,ENSP00000296953,ENSP00000298307,ENSP00000299084,ENSP00000300289,ENSP00000300291,ENSP00000301030,ENSP00000303427,ENSP00000304642,ENSP00000305976,ENSP00000306185,ENSP00000309334,ENSP00000310226,ENSP00000310557,ENSP00000312631,ENSP00000316335,ENSP00000320176,ENSP00000323074,ENSP00000323696,ENSP00000324463,ENSP00000325074,ENSP00000327025,ENSP00000327716,ENSP00000330572,ENSP00000337946,ENSP00000338788,ENSP00000341680,ENSP00000341838,ENSP00000341885,ENSP00000344260,ENSP00000345917,ENSP00000346015,ENSP00000346151,ENSP00000346931,ENSP00000348308,ENSP00000348965,ENSP00000349016,ENSP00000349456,ENSP00000353910,ENSP00000354518,ENSP00000356042,ENSP00000356331,ENSP00000357552,ENSP00000357722,ENSP00000358795,ENSP00000359345,ENSP00000359621,ENSP00000359859,ENSP00000360214,ENSP00000361014,ENSP00000361433,ENSP00000361540,ENSP00000361824,ENSP00000362146,ENSP00000363852,ENSP00000364212,ENSP00000365477,ENSP00000365606,ENSP00000366843,ENSP00000367038,ENSP00000369519,ENSP00000369860,ENSP00000371018,ENSP00000371472,ENSP00000373715,ENSP00000376534,ENSP00000377836,ENSP00000378163,ENSP00000378340,ENSP00000378974,ENSP00000379888,ENSP00000380185,ENSP00000384000,ENSP00000384371,ENSP00000384792,ENSP00000385958,ENSP00000386184,ENSP00000387122,ENSP00000389176,ENSP00000391524,ENSP00000393795,ENSP00000394085,ENSP00000395007,ENSP00000400467,ENSP00000401802,ENSP00000403172,ENSP00000411528,ENSP00000413929,ENSP00000414398,ENSP00000418287,ENSP00000418563,ENSP00000419692,ENSP00000420095,ENSP00000423067,ENSP00000424765,ENSP00000429900,ENSP00000430432,ENSP00000435777,ENSP00000440864,ENSP00000443926,ENSP00000451261,ENSP00000452123,ENSP00000464813,ENSP00000469880,ENSP00000473631,ENSP00000475814,ENSP00000479322,ENSP00000479542,ENSP00000480571,ENSP00000480715,ENSP00000482472,ENSP00000483552,ENSP00000484288 | ADCY3,AMOTL2,ANKRD11,ANTXR2,APOB,ASAP1,ATXN1,ATXN2,BAZ2B,BCL6,C1GALT1,C6orf120,CBLN3,CCDC93,CCNA2,CDA,CDC20,CDCA8,CELF2,CIAO1,CLCC1,CLEC16A,CLIP1,COL4A6,COX6A2,COX6B1,CREBRF,CRTAP,CYP3A7,DCP2,DENND6B,DNAJB11,DNAJC3,DTNBP1,DYNC1H1,ELL2,EXOSC2,EXOSC5,EXOSC7,EZR,FAM134B,FARS2,FNBP4,FURIN,FUT8,GALNT15,GALNT5,GAPDH,GJC1,GPC3,GRIPAP1,GZMA,HCLS1,HDAC2,HIST2H2AA3,IFNGR2,INSM1,IQCE,JMJD6,KCNE3,KIZ,KLHDC2,KPNA5,LIG4,LIMD1,LPP,LRP5,LYAR,MAGI2,MAPK8,MAPK8IP2,MCOLN3,MKNK1,MMP23B,MOSPD2,MRPL34,MRPL4,MTAP,MTG2,MYL12A,MYOD1,NEK9,NEURL1,NIN,NR4A1,NR5A2,NUDT21,OPALIN,PACRG,PAPD7,PDCD4,PDIA3,PDS5A,PEX14,PKNOX1,PLEKHG6,POLR3K,PPARG,PPP2R2A,PSMA3,PSMA4,PSMC6,RAB1B,RAI1,RCOR3,RHOV,RIN3,RORC,RPL15,RPL18A,RPL27,RPL27A,RPL34,RPL5,RPL6,RPL8,RPL9,RPS11,RPS13,RPS14,RPS2,RPS8,RRBP1,RXRA,S100A8,SAMSN1,SIK2,SIRPB1,SLC26A4,SLC44A1,SNAP29,SPRED1,SPTAN1,SRSF5,SYF2,TARDBP,TELO2,TNNI3,U2SURP,UBE2J1,UBE2K,VAMP4,VRK3,WDR36,WDR62,WDR7,WRB,YLPM1,ZBED8,ZC3H15,ZMYND8,ZNF184,ZNF512B,ZNF681,ZNF746,ZNF830 |
| GO:0005654 | nucleoplasm | 55 | 3446 | 0.041 | ENSP00000044462,ENSP00000215730,ENSP00000216455,ENSP00000221233,ENSP00000230859,ENSP00000236273,ENSP00000237853,ENSP00000240185,ENSP00000244769,ENSP00000250003,ENSP00000262319,ENSP00000263773,ENSP00000265564,ENSP00000270625,ENSP00000274026,ENSP00000287820,ENSP00000293860,ENSP00000296953,ENSP00000298307,ENSP00000300291,ENSP00000301030,ENSP00000303427,ENSP00000304642,ENSP00000312631,ENSP00000323074,ENSP00000324463,ENSP00000325074,ENSP00000327025,ENSP00000341885,ENSP00000354518,ENSP00000356331,ENSP00000357552,ENSP00000359345,ENSP00000361014,ENSP00000361433,ENSP00000361540,ENSP00000362146,ENSP00000363852,ENSP00000365606,ENSP00000373715,ENSP00000378974,ENSP00000379888,ENSP00000384371,ENSP00000385958,ENSP00000386184,ENSP00000394085,ENSP00000401802,ENSP00000418563,ENSP00000419692,ENSP00000423067,ENSP00000430432,ENSP00000435777,ENSP00000440864,ENSP00000452123,ENSP00000484288 | ANKRD11,ATXN1,BCL6,CCNA2,CDC20,CDCA8,CREBRF,DCP2,ELL2,EXOSC2,EXOSC5,EXOSC7,FAM134B,FNBP4,GRIPAP1,HDAC2,INSM1,JMJD6,KLHDC2,KPNA5,LIG4,MAPK8,MKNK1,MYOD1,NR4A1,NR5A2,NUDT21,PAPD7,PDS5A,POLR3K,PPARG,PPP2R2A,PSMA3,PSMA4,PSMC6,RAI1,RORC,RPL5,RPS11,RPS13,RPS14,RPS2,RPS8,RXRA,SLC44A1,SNAP29,SRSF5,SYF2,TARDBP,TELO2,U2SURP,WDR36,YLPM1,ZBED8,ZNF830 |
| GO:0031981 | nuclear lumen | 62 | 4030 | 0.0436 | ENSP00000044462,ENSP00000215730,ENSP00000216455,ENSP00000221233,ENSP00000230859,ENSP00000236273,ENSP00000237853,ENSP00000240185,ENSP00000244769,ENSP00000250003,ENSP00000262319,ENSP00000263773,ENSP00000265564,ENSP00000270625,ENSP00000274026,ENSP00000287820,ENSP00000293860,ENSP00000296953,ENSP00000298307,ENSP00000300291,ENSP00000301030,ENSP00000303427,ENSP00000304642,ENSP00000312631,ENSP00000323074,ENSP00000324463,ENSP00000325074,ENSP00000327025,ENSP00000338788,ENSP00000341885,ENSP00000345917,ENSP00000349016,ENSP00000354518,ENSP00000356042,ENSP00000356331,ENSP00000357552,ENSP00000359345,ENSP00000361014,ENSP00000361433,ENSP00000361540,ENSP00000362146,ENSP00000363852,ENSP00000365606,ENSP00000371472,ENSP00000373715,ENSP00000378974,ENSP00000379888,ENSP00000384371,ENSP00000385958,ENSP00000386184,ENSP00000394085,ENSP00000401802,ENSP00000418563,ENSP00000419692,ENSP00000423067,ENSP00000430432,ENSP00000435777,ENSP00000440864,ENSP00000452123,ENSP00000469880,ENSP00000475814,ENSP00000484288 | ANKRD11,ATXN1,BCL6,CCNA2,CDC20,CDCA8,CREBRF,DCP2,ELL2,EXOSC2,EXOSC5,EXOSC7,EZR,FAM134B,FNBP4,GRIPAP1,HDAC2,HIST2H2AA3,INSM1,JMJD6,KLHDC2,KPNA5,LIG4,LYAR,MAPK8,MKNK1,MYOD1,NIN,NR4A1,NR5A2,NUDT21,PAPD7,PDS5A,PEX14,POLR3K,PPARG,PPP2R2A,PSMA3,PSMA4,PSMC6,RAI1,RORC,RPL5,RPS11,RPS13,RPS14,RPS2,RPS8,RXRA,SLC44A1,SNAP29,SRSF5,SYF2,TARDBP,TELO2,U2SURP,VRK3,WDR36,YLPM1,ZBED8,ZC3H15,ZNF830 |

Table S7_VCT_DEGs_Go_Molecular Function

| #term ID | term description | observed gene count | background gene count | false discovery rate | matching proteins in your network (IDs) | matching proteins in your network (labels) |
| --- | --- | --- | --- | --- | --- | --- |
| GO:0003735 | structural constituent of ribosome | 18 | 146 | 1.09E-10 | ENSP00000222247,ENSP00000252602,ENSP00000253099,ENSP00000262584,ENSP00000270625,ENSP00000309334,ENSP00000339795,ENSP00000346015,ENSP00000359345,ENSP00000378163,ENSP00000379506,ENSP00000379888,ENSP00000385958,ENSP00000400467,ENSP00000403172,ENSP00000419117,ENSP00000435777,ENSP00000464813 | MRPL34,MRPL4,RPL14,RPL15,RPL18A,RPL27,RPL27A,RPL34,RPL35A,RPL5,RPL6,RPL7,RPL8,RPL9,RPS11,RPS13,RPS14,RPS8 |
| GO:0005198 | structural molecule activity | 25 | 679 | 4.70E-05 | ENSP00000222247,ENSP00000252602,ENSP00000253099,ENSP00000262319,ENSP00000262584,ENSP00000270625,ENSP00000306461,ENSP00000309334,ENSP00000330572,ENSP00000339795,ENSP00000346015,ENSP00000346151,ENSP00000359345,ENSP00000361824,ENSP00000378163,ENSP00000378340,ENSP00000379506,ENSP00000379888,ENSP00000385958,ENSP00000400467,ENSP00000403172,ENSP00000409544,ENSP00000419117,ENSP00000435777,ENSP00000464813 | CLDN4,COL4A6,MAGI2,MAPK8IP2,MRPL34,MRPL4,RPL14,RPL15,RPL18A,RPL27,RPL27A,RPL34,RPL35A,RPL5,RPL6,RPL7,RPL8,RPL9,RPS11,RPS13,RPS14,RPS8,SPRR1B,SPTAN1,TELO2 |
| GO:0003723 | RNA binding | 23 | 850 | 0.013 | ENSP00000221233,ENSP00000240185,ENSP00000244769,ENSP00000262584,ENSP00000265564,ENSP00000270625,ENSP00000280154,ENSP00000300291,ENSP00000316335,ENSP00000339795,ENSP00000341885,ENSP00000359345,ENSP00000361433,ENSP00000368927,ENSP00000373715,ENSP00000378163,ENSP00000384792,ENSP00000385958,ENSP00000394085,ENSP00000400467,ENSP00000419117,ENSP00000435777,ENSP00000443926 | ATXN1,CELF2,DCP2,EIF1AX,EXOSC2,EXOSC5,EXOSC7,FARS2,JMJD6,NUDT21,PDCD4,RPL34,RPL35A,RPL5,RPL7,RPL8,RPL9,RPS11,RPS13,RPS14,RPS2,TARDBP,WDR62 |
| GO:0019843 | rRNA binding | 6 | 60 | 0.013 | ENSP00000262584,ENSP00000270625,ENSP00000359345,ENSP00000385958,ENSP00000400467,ENSP00000435777 | RPL5,RPL8,RPL9,RPS11,RPS13,RPS14 |
| GO:0004879 | nuclear receptor activity | 5 | 50 | 0.0356 | ENSP00000287820,ENSP00000327025,ENSP00000356331,ENSP00000419692,ENSP00000440864 | NR4A1,NR5A2,PPARG,RORC,RXRA |
| GO:0016896 | exoribonuclease activity, producing 5'-phosphomonoesters | 4 | 29 | 0.0356 | ENSP00000221233,ENSP00000265564,ENSP00000361433,ENSP00000373715 | DCP2,EXOSC2,EXOSC5,EXOSC7 |
| GO:0019899 | enzyme binding | 41 | 2197 | 0.0356 | ENSP00000216455,ENSP00000216487,ENSP00000233242,ENSP00000238616,ENSP00000250003,ENSP00000261427,ENSP00000262319,ENSP00000274026,ENSP00000287820,ENSP00000299084,ENSP00000300291,ENSP00000312631,ENSP00000320176,ENSP00000325074,ENSP00000330572,ENSP00000337946,ENSP00000341838,ENSP00000341885,ENSP00000346151,ENSP00000347742,ENSP00000356042,ENSP00000359345,ENSP00000361540,ENSP00000361824,ENSP00000364145,ENSP00000373734,ENSP00000378974,ENSP00000380178,ENSP00000380185,ENSP00000391524,ENSP00000395007,ENSP00000419692,ENSP00000420211,ENSP00000428340,ENSP00000430432,ENSP00000451261,ENSP00000452123,ENSP00000469880,ENSP00000473631,ENSP00000479542,ENSP00000483552 | ANGPT1,APOB,CCNA2,CDC20,DENND5B,DENND6B,DNAJC3,EZR,FURIN,HCLS1,HDAC2,INSM1,KIZ,MAGI2,MAPK8,MAPK8IP2,MYOD1,NEK9,NUDT21,PACRG,PDZD3,PFKFB1,PLEKHG6,PPARG,PPP2R2A,PSMA3,RGL2,RIN3,RPL5,RPS2,RXRA,SPRED1,SPTAN1,SRSF5,TELO2,TNNI3,UBE2G1,UBE2J1,UBE2K,VRK3,ZNF746 |
| GO:0003707 | steroid hormone receptor activity | 5 | 59 | 0.0361 | ENSP00000287820,ENSP00000327025,ENSP00000356331,ENSP00000419692,ENSP00000440864 | NR4A1,NR5A2,PPARG,RORC,RXRA |

Table S8_VCT_DEGs_Pathways

| #term ID | term description | observed gene count | background gene count | false discovery rate | matching proteins in your network (IDs) | matching proteins in your network (labels) |
| --- | --- | --- | --- | --- | --- | --- |
| HSA-192823 | Viral mRNA Translation | 18 | 86 | 3.66E-14 | ENSP00000222247,ENSP00000262584,ENSP00000270625,ENSP00000309334,ENSP00000339795,ENSP00000341885,ENSP00000346015,ENSP00000359345,ENSP00000378163,ENSP00000379506,ENSP00000379888,ENSP00000385958,ENSP00000400467,ENSP00000403172,ENSP00000419117,ENSP00000435777,ENSP00000464813,ENSP00000473631 | DNAJC3,RPL14,RPL15,RPL18A,RPL27,RPL27A,RPL34,RPL35A,RPL5,RPL6,RPL7,RPL8,RPL9,RPS11,RPS13,RPS14,RPS2,RPS8 |
| HSA-72689 | Formation of a pool of free 40S subunits | 18 | 98 | 1.39E-13 | ENSP00000222247,ENSP00000262584,ENSP00000270625,ENSP00000309334,ENSP00000339795,ENSP00000341885,ENSP00000346015,ENSP00000359345,ENSP00000368927,ENSP00000378163,ENSP00000379506,ENSP00000379888,ENSP00000385958,ENSP00000400467,ENSP00000403172,ENSP00000419117,ENSP00000435777,ENSP00000464813 | EIF1AX,RPL14,RPL15,RPL18A,RPL27,RPL27A,RPL34,RPL35A,RPL5,RPL6,RPL7,RPL8,RPL9,RPS11,RPS13,RPS14,RPS2,RPS8 |
| HSA-156902 | Peptide chain elongation | 17 | 86 | 2.14E-13 | ENSP00000222247,ENSP00000262584,ENSP00000270625,ENSP00000309334,ENSP00000339795,ENSP00000341885,ENSP00000346015,ENSP00000359345,ENSP00000378163,ENSP00000379506,ENSP00000379888,ENSP00000385958,ENSP00000400467,ENSP00000403172,ENSP00000419117,ENSP00000435777,ENSP00000464813 | RPL14,RPL15,RPL18A,RPL27,RPL27A,RPL34,RPL35A,RPL5,RPL6,RPL7,RPL8,RPL9,RPS11,RPS13,RPS14,RPS2,RPS8 |
| HSA-156827 | L13a-mediated translational silencing of Ceruloplasmin expression | 18 | 107 | 2.72E-13 | ENSP00000222247,ENSP00000262584,ENSP00000270625,ENSP00000309334,ENSP00000339795,ENSP00000341885,ENSP00000346015,ENSP00000359345,ENSP00000368927,ENSP00000378163,ENSP00000379506,ENSP00000379888,ENSP00000385958,ENSP00000400467,ENSP00000403172,ENSP00000419117,ENSP00000435777,ENSP00000464813 | EIF1AX,RPL14,RPL15,RPL18A,RPL27,RPL27A,RPL34,RPL35A,RPL5,RPL6,RPL7,RPL8,RPL9,RPS11,RPS13,RPS14,RPS2,RPS8 |
| HSA-2408557 | Selenocysteine synthesis | 17 | 90 | 2.72E-13 | ENSP00000222247,ENSP00000262584,ENSP00000270625,ENSP00000309334,ENSP00000339795,ENSP00000341885,ENSP00000346015,ENSP00000359345,ENSP00000378163,ENSP00000379506,ENSP00000379888,ENSP00000385958,ENSP00000400467,ENSP00000403172,ENSP00000419117,ENSP00000435777,ENSP00000464813 | RPL14,RPL15,RPL18A,RPL27,RPL27A,RPL34,RPL35A,RPL5,RPL6,RPL7,RPL8,RPL9,RPS11,RPS13,RPS14,RPS2,RPS8 |
| HSA-6791226 | Major pathway of rRNA processing in the nucleolus and cytosol | 21 | 179 | 2.72E-13 | ENSP00000221233,ENSP00000222247,ENSP00000262584,ENSP00000265564,ENSP00000270625,ENSP00000309334,ENSP00000339795,ENSP00000341885,ENSP00000346015,ENSP00000359345,ENSP00000361433,ENSP00000378163,ENSP00000379506,ENSP00000379888,ENSP00000385958,ENSP00000400467,ENSP00000403172,ENSP00000419117,ENSP00000423067,ENSP00000435777,ENSP00000464813 | EXOSC2,EXOSC5,EXOSC7,RPL14,RPL15,RPL18A,RPL27,RPL27A,RPL34,RPL35A,RPL5,RPL6,RPL7,RPL8,RPL9,RPS11,RPS13,RPS14,RPS2,RPS8,WDR36 |
| HSA-72706 | GTP hydrolysis and joining of the 60S ribosomal subunit | 18 | 108 | 2.72E-13 | ENSP00000222247,ENSP00000262584,ENSP00000270625,ENSP00000309334,ENSP00000339795,ENSP00000341885,ENSP00000346015,ENSP00000359345,ENSP00000368927,ENSP00000378163,ENSP00000379506,ENSP00000379888,ENSP00000385958,ENSP00000400467,ENSP00000403172,ENSP00000419117,ENSP00000435777,ENSP00000464813 | EIF1AX,RPL14,RPL15,RPL18A,RPL27,RPL27A,RPL34,RPL35A,RPL5,RPL6,RPL7,RPL8,RPL9,RPS11,RPS13,RPS14,RPS2,RPS8 |
| HSA-72764 | Eukaryotic Translation Termination | 17 | 90 | 2.72E-13 | ENSP00000222247,ENSP00000262584,ENSP00000270625,ENSP00000309334,ENSP00000339795,ENSP00000341885,ENSP00000346015,ENSP00000359345,ENSP00000378163,ENSP00000379506,ENSP00000379888,ENSP00000385958,ENSP00000400467,ENSP00000403172,ENSP00000419117,ENSP00000435777,ENSP00000464813 | RPL14,RPL15,RPL18A,RPL27,RPL27A,RPL34,RPL35A,RPL5,RPL6,RPL7,RPL8,RPL9,RPS11,RPS13,RPS14,RPS2,RPS8 |
| HSA-975956 | Nonsense Mediated Decay (NMD) independent of the Exon Junction Complex (EJC) | 17 | 92 | 2.72E-13 | ENSP00000222247,ENSP00000262584,ENSP00000270625,ENSP00000309334,ENSP00000339795,ENSP00000341885,ENSP00000346015,ENSP00000359345,ENSP00000378163,ENSP00000379506,ENSP00000379888,ENSP00000385958,ENSP00000400467,ENSP00000403172,ENSP00000419117,ENSP00000435777,ENSP00000464813 | RPL14,RPL15,RPL18A,RPL27,RPL27A,RPL34,RPL35A,RPL5,RPL6,RPL7,RPL8,RPL9,RPS11,RPS13,RPS14,RPS2,RPS8 |
| HSA-975957 | Nonsense Mediated Decay (NMD) enhanced by the Exon Junction Complex (EJC) | 18 | 112 | 2.72E-13 | ENSP00000222247,ENSP00000262584,ENSP00000270625,ENSP00000309334,ENSP00000325074,ENSP00000339795,ENSP00000341885,ENSP00000346015,ENSP00000359345,ENSP00000378163,ENSP00000379506,ENSP00000379888,ENSP00000385958,ENSP00000400467,ENSP00000403172,ENSP00000419117,ENSP00000435777,ENSP00000464813 | PPP2R2A,RPL14,RPL15,RPL18A,RPL27,RPL27A,RPL34,RPL35A,RPL5,RPL6,RPL7,RPL8,RPL9,RPS11,RPS13,RPS14,RPS2,RPS8 |
| HSA-9010553 | Regulation of expression of SLITs and ROBOs | 20 | 164 | 4.77E-13 | ENSP00000044462,ENSP00000216455,ENSP00000222247,ENSP00000262584,ENSP00000270625,ENSP00000309334,ENSP00000339795,ENSP00000341885,ENSP00000346015,ENSP00000359345,ENSP00000378163,ENSP00000379506,ENSP00000379888,ENSP00000385958,ENSP00000400467,ENSP00000401802,ENSP00000403172,ENSP00000419117,ENSP00000435777,ENSP00000464813 | PSMA3,PSMA4,PSMC6,RPL14,RPL15,RPL18A,RPL27,RPL27A,RPL34,RPL35A,RPL5,RPL6,RPL7,RPL8,RPL9,RPS11,RPS13,RPS14,RPS2,RPS8 |
| HSA-168254 | Influenza Infection | 19 | 148 | 8.65E-13 | ENSP00000222247,ENSP00000262584,ENSP00000270625,ENSP00000309334,ENSP00000339795,ENSP00000341885,ENSP00000346015,ENSP00000357552,ENSP00000359345,ENSP00000378163,ENSP00000379506,ENSP00000379888,ENSP00000385958,ENSP00000400467,ENSP00000403172,ENSP00000419117,ENSP00000435777,ENSP00000464813,ENSP00000473631 | DNAJC3,KPNA5,RPL14,RPL15,RPL18A,RPL27,RPL27A,RPL34,RPL35A,RPL5,RPL6,RPL7,RPL8,RPL9,RPS11,RPS13,RPS14,RPS2,RPS8 |
| HSA-1799339 | SRP-dependent cotranslational protein targeting to membrane | 17 | 109 | 1.08E-12 | ENSP00000222247,ENSP00000262584,ENSP00000270625,ENSP00000309334,ENSP00000339795,ENSP00000341885,ENSP00000346015,ENSP00000359345,ENSP00000378163,ENSP00000379506,ENSP00000379888,ENSP00000385958,ENSP00000400467,ENSP00000403172,ENSP00000419117,ENSP00000435777,ENSP00000464813 | RPL14,RPL15,RPL18A,RPL27,RPL27A,RPL34,RPL35A,RPL5,RPL6,RPL7,RPL8,RPL9,RPS11,RPS13,RPS14,RPS2,RPS8 |
| HSA-5663205 | Infectious disease | 25 | 363 | 2.21E-11 | ENSP00000044462,ENSP00000216455,ENSP00000222247,ENSP00000262584,ENSP00000270625,ENSP00000309334,ENSP00000339795,ENSP00000341885,ENSP00000346015,ENSP00000347742,ENSP00000357552,ENSP00000359345,ENSP00000378163,ENSP00000379506,ENSP00000379888,ENSP00000385958,ENSP00000400467,ENSP00000401802,ENSP00000403172,ENSP00000419117,ENSP00000435777,ENSP00000464813,ENSP00000473631,ENSP00000483552,ENSP00000484288 | DNAJC3,FURIN,KPNA5,LIG4,PDZD3,PSMA3,PSMA4,PSMC6,RPL14,RPL15,RPL18A,RPL27,RPL27A,RPL34,RPL35A,RPL5,RPL6,RPL7,RPL8,RPL9,RPS11,RPS13,RPS14,RPS2,RPS8 |
| HSA-71291 | Metabolism of amino acids and derivatives | 23 | 354 | 5.66E-10 | ENSP00000044462,ENSP00000216455,ENSP00000222247,ENSP00000262584,ENSP00000270625,ENSP00000309334,ENSP00000339795,ENSP00000341885,ENSP00000346015,ENSP00000359345,ENSP00000363852,ENSP00000369519,ENSP00000378163,ENSP00000379506,ENSP00000379888,ENSP00000385080,ENSP00000385958,ENSP00000400467,ENSP00000401802,ENSP00000403172,ENSP00000419117,ENSP00000435777,ENSP00000464813 | FAH,MTAP,PSMA3,PSMA4,PSMC6,RPL14,RPL15,RPL18A,RPL27,RPL27A,RPL34,RPL35A,RPL5,RPL6,RPL7,RPL8,RPL9,RPS11,RPS13,RPS14,RPS2,RPS8,SLC44A1 |
| HSA-72766 | Translation | 21 | 288 | 5.76E-10 | ENSP00000222247,ENSP00000252602,ENSP00000253099,ENSP00000262584,ENSP00000270625,ENSP00000309334,ENSP00000316335,ENSP00000339795,ENSP00000341885,ENSP00000346015,ENSP00000359345,ENSP00000368927,ENSP00000378163,ENSP00000379506,ENSP00000379888,ENSP00000385958,ENSP00000400467,ENSP00000403172,ENSP00000419117,ENSP00000435777,ENSP00000464813 | EIF1AX,FARS2,MRPL34,MRPL4,RPL14,RPL15,RPL18A,RPL27,RPL27A,RPL34,RPL35A,RPL5,RPL6,RPL7,RPL8,RPL9,RPS11,RPS13,RPS14,RPS2,RPS8 |
| HSA-392499 | Metabolism of proteins | 55 | 1948 | 9.71E-10 | ENSP00000044462,ENSP00000210060,ENSP00000216455,ENSP00000221233,ENSP00000222247,ENSP00000233242,ENSP00000252602,ENSP00000253099,ENSP00000258317,ENSP00000259056,ENSP00000261427,ENSP00000262584,ENSP00000265564,ENSP00000270625,ENSP00000274026,ENSP00000287820,ENSP00000296953,ENSP00000300289,ENSP00000309334,ENSP00000310226,ENSP00000316335,ENSP00000334448,ENSP00000339795,ENSP00000341885,ENSP00000344260,ENSP00000346015,ENSP00000348965,ENSP00000349016,ENSP00000353910,ENSP00000359345,ENSP00000361433,ENSP00000361540,ENSP00000361824,ENSP00000362146,ENSP00000368927,ENSP00000373715,ENSP00000377836,ENSP00000378163,ENSP00000379506,ENSP00000379888,ENSP00000380178,ENSP00000385958,ENSP00000389176,ENSP00000400467,ENSP00000401802,ENSP00000403172,ENSP00000414398,ENSP00000419117,ENSP00000419692,ENSP00000430432,ENSP00000435777,ENSP00000464813,ENSP00000473631,ENSP00000475814,ENSP00000483552 | APOB,C1GALT1,CCNA2,CDC20,CDCA8,CREBRF,DCP2,DHPS,DNAJB11,DNAJC3,DYNC1H1,EIF1AX,EXOSC2,EXOSC5,EXOSC7,FARS2,FURIN,FUT8,GALNT15,GALNT5,GNG2,GPC3,HDAC2,HIST2H2AA3,MRPL34,MRPL4,NPL,PDIA3,PEX14,PPARG,PSMA3,PSMA4,PSMC6,RAB1B,RPL14,RPL15,RPL18A,RPL27,RPL27A,RPL34,RPL35A,RPL5,RPL6,RPL7,RPL8,RPL9,RPS11,RPS13,RPS14,RPS2,RPS8,RXRA,SPTAN1,UBE2G1,UBE2K |
| HSA-8953854 | Metabolism of RNA | 30 | 652 | 1.38E-09 | ENSP00000044462,ENSP00000216455,ENSP00000221233,ENSP00000222247,ENSP00000236273,ENSP00000262584,ENSP00000265564,ENSP00000270625,ENSP00000300291,ENSP00000309334,ENSP00000325074,ENSP00000339795,ENSP00000341885,ENSP00000346015,ENSP00000359345,ENSP00000361433,ENSP00000373715,ENSP00000378163,ENSP00000379506,ENSP00000379888,ENSP00000385958,ENSP00000400467,ENSP00000401802,ENSP00000403172,ENSP00000418563,ENSP00000419117,ENSP00000423067,ENSP00000435777,ENSP00000452123,ENSP00000464813 | DCP2,EXOSC2,EXOSC5,EXOSC7,NUDT21,PPP2R2A,PSMA3,PSMA4,PSMC6,RPL14,RPL15,RPL18A,RPL27,RPL27A,RPL34,RPL35A,RPL5,RPL6,RPL7,RPL8,RPL9,RPS11,RPS13,RPS14,RPS2,RPS8,SRSF5,SYF2,U2SURP,WDR36 |
| HSA-422475 | Axon guidance | 25 | 541 | 5.68E-08 | ENSP00000044462,ENSP00000216455,ENSP00000217652,ENSP00000222247,ENSP00000262584,ENSP00000270625,ENSP00000309334,ENSP00000339795,ENSP00000341885,ENSP00000346015,ENSP00000356042,ENSP00000359345,ENSP00000361824,ENSP00000378163,ENSP00000378338,ENSP00000378974,ENSP00000379506,ENSP00000379888,ENSP00000385958,ENSP00000400467,ENSP00000401802,ENSP00000403172,ENSP00000419117,ENSP00000435777,ENSP00000464813 | EZR,GIT1,MAPK8,MYL12A,PSMA3,PSMA4,PSMC6,RPL14,RPL15,RPL18A,RPL27,RPL27A,RPL34,RPL35A,RPL5,RPL6,RPL7,RPL8,RPL9,RPS11,RPS13,RPS14,RPS2,RPS8,SPTAN1 |
| HSA-1266738 | Developmental Biology | 34 | 1023 | 2.17E-07 | ENSP00000044462,ENSP00000216455,ENSP00000217652,ENSP00000222247,ENSP00000250003,ENSP00000262584,ENSP00000270625,ENSP00000287820,ENSP00000291547,ENSP00000301455,ENSP00000306461,ENSP00000309334,ENSP00000312631,ENSP00000339795,ENSP00000341885,ENSP00000346015,ENSP00000356042,ENSP00000359345,ENSP00000361824,ENSP00000378163,ENSP00000378338,ENSP00000378974,ENSP00000379506,ENSP00000379888,ENSP00000385958,ENSP00000400467,ENSP00000401802,ENSP00000403172,ENSP00000419117,ENSP00000419692,ENSP00000435777,ENSP00000464813,ENSP00000475814,ENSP00000483552 | ANGPTL4,EZR,FURIN,GIT1,HIST2H2AA3,INSM1,MAPK8,MYL12A,MYOD1,PKNOX1,PPARG,PSMA3,PSMA4,PSMC6,RPL14,RPL15,RPL18A,RPL27,RPL27A,RPL34,RPL35A,RPL5,RPL6,RPL7,RPL8,RPL9,RPS11,RPS13,RPS14,RPS2,RPS8,RXRA,SPRR1B,SPTAN1 |
| HSA-1643685 | Disease | 33 | 1018 | 6.40E-07 | ENSP00000044462,ENSP00000216455,ENSP00000222247,ENSP00000230859,ENSP00000262584,ENSP00000265715,ENSP00000270625,ENSP00000294304,ENSP00000299084,ENSP00000309334,ENSP00000339795,ENSP00000341885,ENSP00000346015,ENSP00000347742,ENSP00000357552,ENSP00000358795,ENSP00000359345,ENSP00000377836,ENSP00000378163,ENSP00000379506,ENSP00000379888,ENSP00000385958,ENSP00000400467,ENSP00000401802,ENSP00000403172,ENSP00000419117,ENSP00000430432,ENSP00000435777,ENSP00000440864,ENSP00000464813,ENSP00000473631,ENSP00000483552,ENSP00000484288 | DNAJC3,FURIN,GPC3,HDAC2,KPNA5,LIG4,LRP5,NEURL1,NR4A1,PAPD7,PDZD3,PSMA3,PSMA4,PSMC6,RPL14,RPL15,RPL18A,RPL27,RPL27A,RPL34,RPL35A,RPL5,RPL6,RPL7,RPL8,RPL9,RPS11,RPS13,RPS14,RPS2,RPS8,SLC26A4,SPRED1 |
| HSA-72695 | Formation of the ternary complex, and subsequently, the 43S complex | 6 | 49 | 0.00061 | ENSP00000270625,ENSP00000341885,ENSP00000368927,ENSP00000379888,ENSP00000385958,ENSP00000435777 | EIF1AX,RPS11,RPS13,RPS14,RPS2,RPS8 |
| HSA-450531 | Regulation of mRNA stability by proteins that bind AU-rich elements | 7 | 83 | 0.0011 | ENSP00000044462,ENSP00000216455,ENSP00000221233,ENSP00000265564,ENSP00000361433,ENSP00000373715,ENSP00000401802 | DCP2,EXOSC2,EXOSC5,EXOSC7,PSMA3,PSMA4,PSMC6 |
| HSA-72649 | Translation initiation complex formation | 6 | 55 | 0.0011 | ENSP00000270625,ENSP00000341885,ENSP00000368927,ENSP00000379888,ENSP00000385958,ENSP00000435777 | EIF1AX,RPS11,RPS13,RPS14,RPS2,RPS8 |
| HSA-72702 | Ribosomal scanning and start codon recognition | 6 | 55 | 0.0011 | ENSP00000270625,ENSP00000341885,ENSP00000368927,ENSP00000379888,ENSP00000385958,ENSP00000435777 | EIF1AX,RPS11,RPS13,RPS14,RPS2,RPS8 |
| HSA-450385 | Butyrate Response Factor 1 (BRF1) binds and destabilizes mRNA | 4 | 17 | 0.0013 | ENSP00000221233,ENSP00000265564,ENSP00000361433,ENSP00000373715 | DCP2,EXOSC2,EXOSC5,EXOSC7 |
| HSA-450513 | Tristetraprolin (TTP, ZFP36) binds and destabilizes mRNA | 4 | 17 | 0.0013 | ENSP00000221233,ENSP00000265564,ENSP00000361433,ENSP00000373715 | DCP2,EXOSC2,EXOSC5,EXOSC7 |
| HSA-450604 | KSRP (KHSRP) binds and destabilizes mRNA | 4 | 17 | 0.0013 | ENSP00000221233,ENSP00000265564,ENSP00000361433,ENSP00000373715 | DCP2,EXOSC2,EXOSC5,EXOSC7 |
| HSA-381119 | Unfolded Protein Response (UPR) | 7 | 94 | 0.002 | ENSP00000221233,ENSP00000265564,ENSP00000296953,ENSP00000361433,ENSP00000373715,ENSP00000414398,ENSP00000473631 | CREBRF,DCP2,DNAJB11,DNAJC3,EXOSC2,EXOSC5,EXOSC7 |
| HSA-380994 | ATF4 activates genes | 4 | 25 | 0.0044 | ENSP00000221233,ENSP00000265564,ENSP00000361433,ENSP00000373715 | DCP2,EXOSC2,EXOSC5,EXOSC7 |
| HSA-68882 | Mitotic Anaphase | 9 | 186 | 0.0046 | ENSP00000044462,ENSP00000216455,ENSP00000303427,ENSP00000325074,ENSP00000348965,ENSP00000361540,ENSP00000362146,ENSP00000401802,ENSP00000479322 | CDC20,CDCA8,CLIP1,DYNC1H1,PDS5A,PPP2R2A,PSMA3,PSMA4,PSMC6 |
| HSA-68886 | M Phase | 12 | 343 | 0.0077 | ENSP00000044462,ENSP00000216455,ENSP00000238616,ENSP00000303427,ENSP00000310226,ENSP00000325074,ENSP00000348965,ENSP00000361540,ENSP00000362146,ENSP00000401802,ENSP00000475814,ENSP00000479322 | CDC20,CDCA8,CLIP1,DYNC1H1,HIST2H2AA3,NEK9,PDS5A,PPP2R2A,PSMA3,PSMA4,PSMC6,RAB1B |
| HSA-1430728 | Metabolism | 38 | 2032 | 0.0107 | ENSP00000044462,ENSP00000216455,ENSP00000222247,ENSP00000229239,ENSP00000233242,ENSP00000246554,ENSP00000256578,ENSP00000260600,ENSP00000262584,ENSP00000270625,ENSP00000287820,ENSP00000301455,ENSP00000309334,ENSP00000334448,ENSP00000339795,ENSP00000341885,ENSP00000346015,ENSP00000359345,ENSP00000363852,ENSP00000364145,ENSP00000364212,ENSP00000369519,ENSP00000377836,ENSP00000378163,ENSP00000379506,ENSP00000379888,ENSP00000385080,ENSP00000385958,ENSP00000400467,ENSP00000401802,ENSP00000403172,ENSP00000418287,ENSP00000419117,ENSP00000419692,ENSP00000425487,ENSP00000435777,ENSP00000464813,ENSP00000480571 | ADCY3,AMPD2,ANGPTL4,APOB,CDA,CIAO1,COX6B1,CYP3A7,FAH,GAPDH,GNG2,GPC3,INPP4B,MTAP,PFKFB1,PPARG,PSMA3,PSMA4,PSMC6,RPL14,RPL15,RPL18A,RPL27,RPL27A,RPL34,RPL35A,RPL5,RPL6,RPL7,RPL8,RPL9,RPS11,RPS13,RPS14,RPS2,RPS8,RXRA,SLC44A1 |
| HSA-2467813 | Separation of Sister Chromatids | 8 | 178 | 0.0143 | ENSP00000044462,ENSP00000216455,ENSP00000303427,ENSP00000348965,ENSP00000361540,ENSP00000362146,ENSP00000401802,ENSP00000479322 | CDC20,CDCA8,CLIP1,DYNC1H1,PDS5A,PSMA3,PSMA4,PSMC6 |
| HSA-597592 | Post-translational protein modification | 28 | 1366 | 0.0151 | ENSP00000044462,ENSP00000210060,ENSP00000216455,ENSP00000233242,ENSP00000258317,ENSP00000259056,ENSP00000261427,ENSP00000274026,ENSP00000287820,ENSP00000300289,ENSP00000310226,ENSP00000341885,ENSP00000344260,ENSP00000348965,ENSP00000349016,ENSP00000353910,ENSP00000361540,ENSP00000361824,ENSP00000362146,ENSP00000377836,ENSP00000380178,ENSP00000389176,ENSP00000401802,ENSP00000419692,ENSP00000430432,ENSP00000473631,ENSP00000475814,ENSP00000483552 | APOB,C1GALT1,CCNA2,CDC20,CDCA8,DHPS,DNAJC3,DYNC1H1,FURIN,FUT8,GALNT15,GALNT5,GPC3,HDAC2,HIST2H2AA3,NPL,PDIA3,PEX14,PPARG,PSMA3,PSMA4,PSMC6,RAB1B,RPS2,RXRA,SPTAN1,UBE2G1,UBE2K |
| HSA-429958 | mRNA decay by 3' to 5' exoribonuclease | 3 | 16 | 0.0156 | ENSP00000221233,ENSP00000265564,ENSP00000361433 | EXOSC2,EXOSC5,EXOSC7 |
| HSA-174184 | Cdc20:Phospho-APC/C mediated degradation of Cyclin A | 5 | 68 | 0.0165 | ENSP00000044462,ENSP00000216455,ENSP00000274026,ENSP00000361540,ENSP00000401802 | CCNA2,CDC20,PSMA3,PSMA4,PSMC6 |
| HSA-383280 | Nuclear Receptor transcription pathway | 4 | 39 | 0.0165 | ENSP00000287820,ENSP00000327025,ENSP00000419692,ENSP00000440864 | NR4A1,PPARG,RORC,RXRA |
| HSA-176408 | Regulation of APC/C activators between G1/S and early anaphase | 5 | 76 | 0.0239 | ENSP00000044462,ENSP00000216455,ENSP00000274026,ENSP00000361540,ENSP00000401802 | CCNA2,CDC20,PSMA3,PSMA4,PSMC6 |
| HSA-69278 | Cell Cycle, Mitotic | 13 | 483 | 0.0354 | ENSP00000044462,ENSP00000216455,ENSP00000238616,ENSP00000274026,ENSP00000303427,ENSP00000310226,ENSP00000325074,ENSP00000348965,ENSP00000361540,ENSP00000362146,ENSP00000401802,ENSP00000475814,ENSP00000479322 | CCNA2,CDC20,CDCA8,CLIP1,DYNC1H1,HIST2H2AA3,NEK9,PDS5A,PPP2R2A,PSMA3,PSMA4,PSMC6,RAB1B |
| HSA-174113 | SCF-beta-TrCP mediated degradation of Emi1 | 4 | 52 | 0.0381 | ENSP00000044462,ENSP00000216455,ENSP00000361540,ENSP00000401802 | CDC20,PSMA3,PSMA4,PSMC6 |
| HSA-429914 | Deadenylation-dependent mRNA decay | 4 | 55 | 0.0453 | ENSP00000221233,ENSP00000265564,ENSP00000361433,ENSP00000373715 | DCP2,EXOSC2,EXOSC5,EXOSC7 |
